# A DFT and QTAIM insight into ethylene oxide adsorption on the surfaces of pure and metal-decorated inorganic fullerene-like nanoclusters

**DOI:** 10.1016/j.heliyon.2023.e19407

**Published:** 2023-08-25

**Authors:** Palash Dhali, Adita Afrin Oishi, Antu Das, Md Rakib Hossain, Farid Ahmed, Debashis Roy, Md Mehade Hasan

**Affiliations:** a Department of Physics, Jashore University of Science and Technology, Jashore, 7408, Bangladesh; b Department of Physics, Bangabandhu Sheikh Mujibur Rahman Science and Technology University, Gopalganj, 8100, Bangladesh; c Department of Physics, Jahangirnagar University, Savar, Dhaka, 1342, Bangladesh

**Keywords:** Ethylene oxide, Fullerene-like nanocluster, Metal decoration, DFT, Gas sensor

## Abstract

In this industrial era, the use of low-dimensional nanomaterials as gas sensors for environmental monitoring has received enormous interest. To develop an effective sensing method for ethylene oxide (EO), DFT computations are conducted using method ωB97X-D and B3LYP with 6-31G(d,p) basis set to evaluate the adsorption behavior of ethylene oxide gas on the surfaces of pristine, as well as Scandium and Titanium decorated B_12_N_12_, Al_12_N_12,_ and Al_12_P_12_ nanocages. Several properties like structural, physical, and electronic are studied methodically to better understand the sensing behavior. Scandium-decorated aluminum phosphate and boron nitride nanocages were shown to perform better in terms of adsorption properties. The short recovery time observed in this study is beneficial for the repetitive use of the gas sensor. The Natural Bond Orbital and molecular electrostatic potential analysis demonstrated a substantial quantity of charge transfer from adsorbate to adsorbents. The bandgap alternation after adsorption shows an influence of adsorption on electronic properties. The interactions of adsorbate and adsorbents are further studied using the ultraviolet–visible predicted spectrum, and quantum theory of atoms in molecules all of which yielded promising findings.

## 1 Introduction

Air pollution has been a severe problem in recent years as a result of excessive industrialization and urbanization. The growing emission of hazardous gases makes nanomaterial-based gas monitors more and more vital. Understanding the key variables that influence the interaction between these gas molecules and nanomaterials is currently a major challenge in the semiconductor industry [[1], [2], [3]]. Ethylene oxide (EO) is a hazardous gas that is symbolized as $C_{2}H_{4}O$ and also known as Oxirane or Epoxyethane. It is a frequently used material in the modern world for manufacturing solvents, antifreeze materials, textiles, industrial/clinical sterilants, and adhesives. Due to its irritating, combustible, poisonous, and toxic nature, upon inhalation of EO, severe nausea, vomiting, bronchitis, and neurological problems can occur. When breathed directly, eyes, skin, and respiratory systems can be irritated by EO vapors, and contact with the skin for an extended period might result in delayed burns and allergy symptoms. Long-time inhalation of EO can cause pancreatic cancer, stomach cancer, leukemia, brain cancer, etc [[4], [5], [6], [7]]. Some previous findings showed the risk of miscarriages among female workers as a result of EO exposure. There have been cases of neurological conditions and even death upon EO inhalation [8,9]. For environmental safety, it is therefore required to detect and eliminate this gas from the air.

Functional nanostructures have recently gained the scientific community's attention as a consequence of their exceptional physical and chemical characteristics [[10], [11], [12]]. A major part of recent advances in nanomaterials research is the identification of appropriate surfaces for the selective adsorption of certain molecules while the sensing capabilities of various surfaces have been explored [13,14]. Group III-V semiconductors are particularly well-suited for several technologies and these materials have several properties that also make them particularly well-suited for gas-sensing applications. These characteristics include their small size and synthesis ease, cheap cost, and repeatability [15,16]. Due to the exceptional stability and outstanding electronic, physical, and chemical properties, ${Al}_{12}N_{12}$, ${Al}_{12}P_{12}$, $B_{12}N_{12}$, and $B_{12}P_{12}$ these four are considered as most influential nanocages [17,18]. These fullerene-like nanostructures have been explored on several occasions for a variety of applications such as Hydrogen Storage [19], Drug Delivery [20,21], Lithium-ion storage [22], Single atom catalyst [23], gas sensing [24], etc. For instance, the nanocage $B_{12}N_{12}\left(BN\right)$ was synthesized by laser desorption time-of-flight mass spectrometry by Oku et al. [25]. Several recent investigations have demonstrated that $B_{12}N_{12}$ nanocluster has superior adsorption characteristics for a wide variety of gaseous molecules [24,[26], [27], [28]]. Moreover, ${Al}_{12}N_{12}$ (AlN) is a nanocluster with a unique combination of physical and electrical characteristics that may be fabricated in planar, tubular, or spherical forms. Each member of this group is well-known as a semiconductor material with a considerable bandgap and a variety of advantageous properties [29]. In addition, Al_12_P_12_(AlP) nanocluster is intriguing to researchers due to its good electrical structure, and several theoretical adsorption investigations have been undertaken on both AlN and AlP nanocages with favorable results [[30], [31], [32], [33], [34]]. Surface modification by doping is a prominent method for improving the sensing behavior and reactivity of fullerene-like nanostructures. Many investigations in the literature reported that the incorporation of metallic elements into fullerene-like structures has resulted in notable enhancements in their sensing capabilities [24,[34], [35], [36], [37], [38]]. For instance, multiple studies showed that doping Scandium(Sc) greatly enhances the sensing capabilities of low-dimensional materials [[39], [40], [41]], similarly, Titanium(Ti) doping [42,43] to several nanomaterials has shown to greatly improve their sensitivity toward tiny molecules. Considering all these recent scientific reports, we have been motivated to choose these nanoclusters and made their surface modifications for sensing EO gas.

The primary aim of our present research is to investigate the possible interactions between EO gas with pristine, as well as Sc and Ti-doped fullerene-like B_12_N_12_, Al_12_N_12_, and Al_12_P_12_ nanostructures, by utilizing density functional theory as a theoretical framework. To understand the interactions among the gas molecule and nanocages we include all conceivable relaxed configurations of considered systems and investigated them in terms of the adsorption energy, basis set superposition error-corrected energy, net charge transfer, QTAIM analysis, UV–vis spectra analysis, HOMO, and LUMO distributions.

## 2 Computational method

All relaxed configurations and essential characteristics calculations are conducted by utilizing the Gaussian 09 [44] software package to ascertain the geometries of adsorbed EO on the surfaces of pristine along with Sc and Ti-doped BN,AlN,andAlP nanoclusters. Density functional theory (DFT) calculations are conducted for geometry optimizations, charge transfer, molecular electrostatic potential (MEP), and density of states (DOSs) analysis on ωB97X-D/6-31G(d,p) and B3LYP/6-31G (d,p) level of theories. Becke's (B3) exchange functional was combined with Lee, Yang, and Parr's (LYP) correlation functional, resulting in the B3LYP level of theory [45,46] and ωB97X-D is a range-separated variant of Becke's 97 functional that includes extra dispersion correction [47,48]. Both ωB97X-D and B3LYP have already established themselves as accurate and reliable functionals for researching nanostructured materials at a reasonable cost. For assessing data close to experimental, these two are better qualified than other functionals [[48], [49], [50], [51], [52]]. However, as the system gets bigger, concerns with the B3LYP functional start to appear which include inaccuracy for heats of formation, underestimated reaction barrier heights, inaccurate representations of van der Waals interactions, and inconsistent energy ordering of isomers [53] but, B3LYP performs comparatively better when computing electronic properties and frontier molecular orbitals of a system [54]. Whereas, ωB97X-D functional produces results with adequate precision for thermochemistry, kinetics, and non-covalent interactions. Several studies showed that ωB97X-D density functional performs considerably better for covalent systems and kinetics than other empirical dispersion-corrected density functionals [47,55]. In this study, we employed the B3LYP functional with double zeta 6-31G(d,p) basis to get an overall decent description of the examined system, and the ωB97X-D with 6-31G(d,p) basis set for obtaining enough precision for thermochemistry, kinetics, and non-covalent interactions. After completing geometry optimization, the same level of theories have been used further to evaluate the frequency and excited state (TD-SCF) calculations on investigated bare, doped, and complex nanostructures; to investigate the variation in enthalpy (ΔH), variation in Gibbs free energy (ΔG), and variation in entropy (ΔS). Additionally, UV–Vis spectra graphs are plotted and exploration is done before and after adsorption. For the analysis of the quantum theory of atoms in molecules (QTAIM) [56]; AIMAll [57] program was used. To verify the investigated adsorbent system's stability; cohesive energy $E_{Cohesive}$ is measured by utilizing this equation (1) below [24,58]

(1) $$E_{Cohesive}=\frac{1}{m}\left(E_{Nanocage}-aE_{A}-bE_{B}-nE_{N}-pE_{P}-dE_{D}\right)$$

where $E_{Nanocage}$ is the energy of the nanocage; $E_{A}$, $E_{B}$, $E_{N}$, $E_{P}$ and $E_{D}$ are the total energy of isolated aluminum, boron, nitrogen, phosphorus, and doped atom respectively and a, b, n, p, and d are the number of aluminum, boron, nitrogen, phosphorus, and doped atom respectively in the nanocages whereas m is the total number of constituent atoms of the nanocage.

The adsorption energy ($E_{Ads.}$) of the complex structures are assessed by equation (2) [59,60]

(2) $$E_{Ads.}=E_{Complex}-E_{Nanocage}-E_{gas}$$

At Temperature = 298.15 K and Pressure = 1 atm, temperature-dependent thermodynamic parameters such as changes in enthalpy (ΔH), changes in Gibbs free energy (ΔG), and changes in entropy (ΔS) are examined by equations (3a), (3b), and (3c) as in the literature [58,59] to further acquire a better insight into the interactions of adsorbate with adsorbents and ensure that at the potential energy surface, all of the assessed configurations are matched to a minimum.

(3a) $$\Delta H=H_{Complex}-H_{Nanocage}-H_{gas}$$

(3b) $$\Delta G=G_{Complex}-G_{Nanocage}-G_{gas}$$

(3c) ΔS=(ΔH−ΔG)/T

where in equation (2), $E_{Complex}$, $E_{Nanocage}$ and $E_{gas}$ stand for the total energy of complex structures, nanocages, and gas molecules respectively; besides in equation (3a), $H_{Complex}$, $H_{Nanocage}$ and $H_{gas}$ are represented as the sum of electronic and thermal enthalpies of complex structures, nanocages, and gas molecules respectively. In equation (3b), $G_{Complex}$, $G_{Nanocage}$, $G_{gas}$ are defined as the sum of electronic and thermal free energies of complex, nanocage, and gas respectively here. Investigation of the electronic properties of all geometries is accomplished by the highest occupied molecular orbital (HOMO), lowest unoccupied molecular orbital (LUMO) level, and their associated energy analysis. The electron affinity(A) and ionization potential(I) are represented by the negatively valued energies of the HOMO ($E_{HOMO}$) and LUMO ($E_{LUMO}$), respectively i.e., I = -E_HOMO_ and A = -E_LUMO_ [50]. Using equations (4), (5) the reactivity of all structures such as chemical potential (μ), global hardness (ƞ), global softness (S), and global electrophilicity (ω) are investigated [24,61].

(4) $$\mu =-\frac{\left(I+A\right)}{2}$$

(5) $$\eta =\frac{\left(I-A\right)}{2}$$

(6) $$S=\frac{1}{2\eta }$$

(7) $$\omega =\frac{\mu^{2}}{2\eta }$$

To eliminate basis set superposition error(BSSE) and correct the system's electrical strength, equation (8) is used to assess the proper interaction between gas and adsorbents [21].

(8) $$E_{Ads,CP}=E_{Ads.}+E_{BSSE}$$

where, in equation (8), $E_{Ads,CP}$ represents the counterpoise corrected adsorption energy and $E_{BSSE}$ the basis set superposition error.

## 3 Results and discussion

### 3.1 Geometry analysis of adsorbents

Initially, all geometries have been optimized at the ground state. The doping process of BN, AlN, and AlP nanostructures is accomplished by placing a foreign atom (Sc, Ti) between two six-membered rings and one four-membered ring, hence removing a B(boron) atom from pristine BN cages and an Al(aluminum) atom from both pristine AlN and AlP cages. All investigated nanocages here are with C1 symmetry and 66 degrees of freedom and are composed of eight six-membered rings(hexagonal) and six four-membered rings (tetragonal) [62].

The B–N bond length in pristine BN nanocage ranges from 1.44 Å to 1.48 Å whereas the Al–N bond length in pristine AlN nanocage is 1.78 Å to 1.85 Å. The Al–P bond length in pristine AlP nanocage is about 2.27 Å to 2.32 Å and these observations are consistent with prior studies [35,63,64]. For decorated BN nanocage, Sc–N and Ti–N bond lengths are 1.92 Å to 1.99 Å. The Sc–N and Ti–N bond lengths in decorated AlN nanocages are 1.89 Å to 2.03 Å and Sc–P and Ti–P bond lengths in decorated AlP nanocages are 2.39 Å to 2.49 Å.

**Fig. 1:**
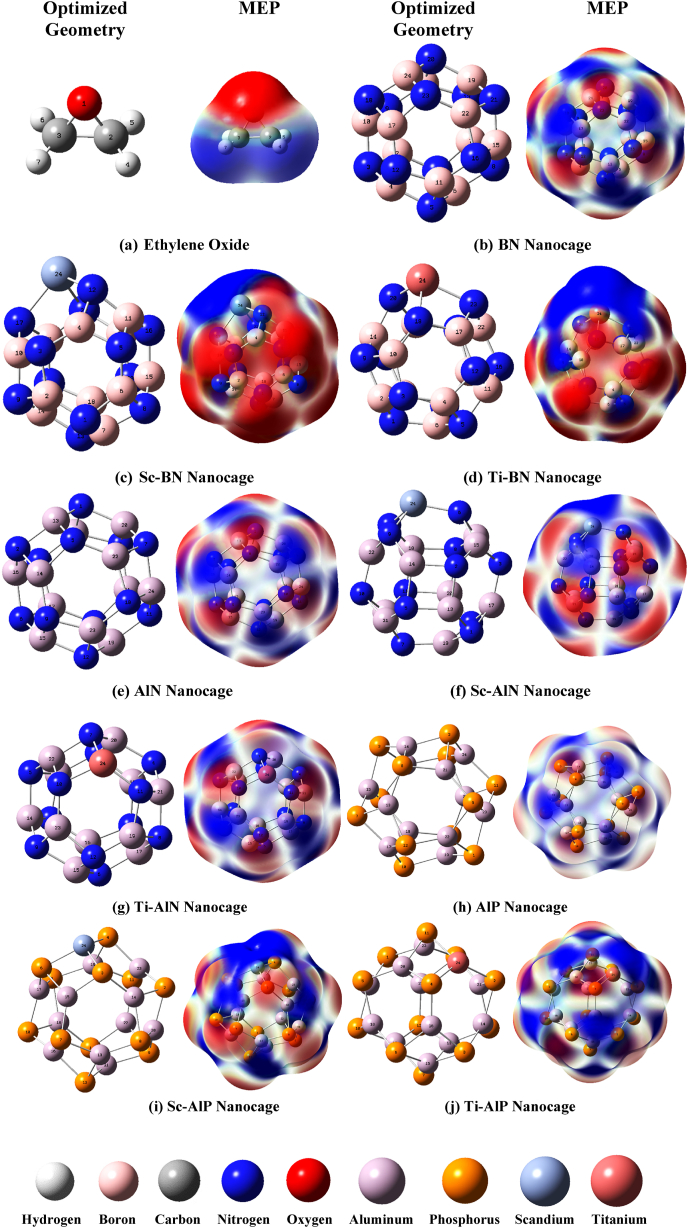
The Optimized Geometry and Molecular Electrostatic Potential (MEP) figures of (a) Ethylene Oxide, (b) BN (B_12_N_12_), (c) Sc-BN (ScB_11_N_12_), (d) Ti-BN (TiB_11_N_12_), (e) AlN (Al_12_N_12_), (f) Sc-AlN (ScAl_11_N_12_), (g) Ti–AlN (TiAl_11_N_12_), (h) AlP (Al_12_P_12_), (i) Sc-AlP (ScAl_11_P_12_), and (j) Ti–AlP (TiAl_11_P_12_) adsorbent nanocages. The color scheme (Red to blue) for the MEP surface ranges from −0.01 a.u. to +0.01 a.u., indicating the electron-rich and electron-deficient regions of the surface, respectively in ωB97X-D/6-31G(d,p) method. The MEP surfaces are generated with 0.0004 electron/bohr^3^ iso-value.

Nearly identical bond lengths for all nanocages are observed for both methods. In addition, bond angles and dihedral angles are evaluated in this literature. Since the bond and dihedral angles are nearly identical for both methods, only the bond and dihedral angles estimated using the B3LYP/6-31G(d,p) method are presented here(see Table S1). Our investigation shows that $B_{12}N_{12}$ and ${Al}_{12}N_{12}$ nanocages have smaller dihedral angles than ${Al}_{12}P_{12}$ nanocages. The bond and dihedral angles observed for pristine nanocages in this literature are nearly identical to those obtained in prior work by Ali Shokuhi Rad et al. [64]. Corresponding relaxed geometries of gas and nanocages in both methods are shown in Fig. 1 and Fig. S1. The stability, sensitivity, and reactivity of bare BN, AlN, and AlP nanocages are changed when they are decorated with foreign atoms, and FMO (frontier molecular orbital) analysis is used to determine this. Significant alterations in the energy levels of the highest occupied molecular orbitals (E_HOMO_) and the lowest unoccupied molecular orbitals (E_LUMO_) have been observed after the decoration of the pristine nanocages(See Table 3 and S4; Figs. S2 and S3). The maximum bandgap is observed for pure BN nanocage and the minimum is for Ti-decorated AlN. The band-gap values of pristine nanocages in this existing study are analogous to previous findings [65,66]. In DFT calculations, FMOs are acquainted as Khon-Sham orbitals, and according to Koopman's theorem, HOMO energy ($E_{HOMO}$) is linked with ionization potential (I), whilst LUMO energy ($E_{LUMO}$) is with electron affinity (A) [35]. To learn about the geometrical stability of nanocages, the cohesive energies are evaluated by using Equation (1). For ωB97X-D/6-31G(d,p) method obtained energies are −7.60 eV, −7.53 eV, −7.58 eV, −5.96 eV, 6.02 eV, 6.08 eV, 4.36 eV, 4.39 eV, and 4.44 eV for pure BN, Sc-BN, and Ti-BN, pure AlN, Sc-AlN, Ti–AlN, pure AlP, Sc-AlP, and Ti–AlP nanocages respectively. The cohesive energies of ωB97X-D/6-31G(d,p) are slightly altered by the implementation of the B3LYP/6-31G(d,p) methodology, in B3LYP the energies are −7.46 eV, −7.39 eV, −7.45 eV, −5.79 eV, −5.86 eV, −5.93 eV, −4.07 eV, −4.11 eV, −4.17 eV for pristine BN cage, Sc-BN cage, Ti-BN cage, pristine AlN cage, Sc-AlN cage, Ti–AlN cage, pristine AlP cage, Sc-AlP cage, Ti–AlP cage respectively. Negative and higher numbered cohesive energies imply good stability toward these nanocages [50]. In the presence of IR, the vibrational modes are analyzed to assess geometrical stability. Each nanocage studied here had a positive frequency mode, which predicted that the geometry of the adsorbents corresponds to a minimum(either local or global) on the potential energy surface of the system. The molecular electrostatic potential of adsorbents has also been analyzed to predict probable adsorption sites between adsorbate (EO) and adsorbents [35].

### 3.2 Adsorption energy and thermodynamic parameters

**Fig. 2:**
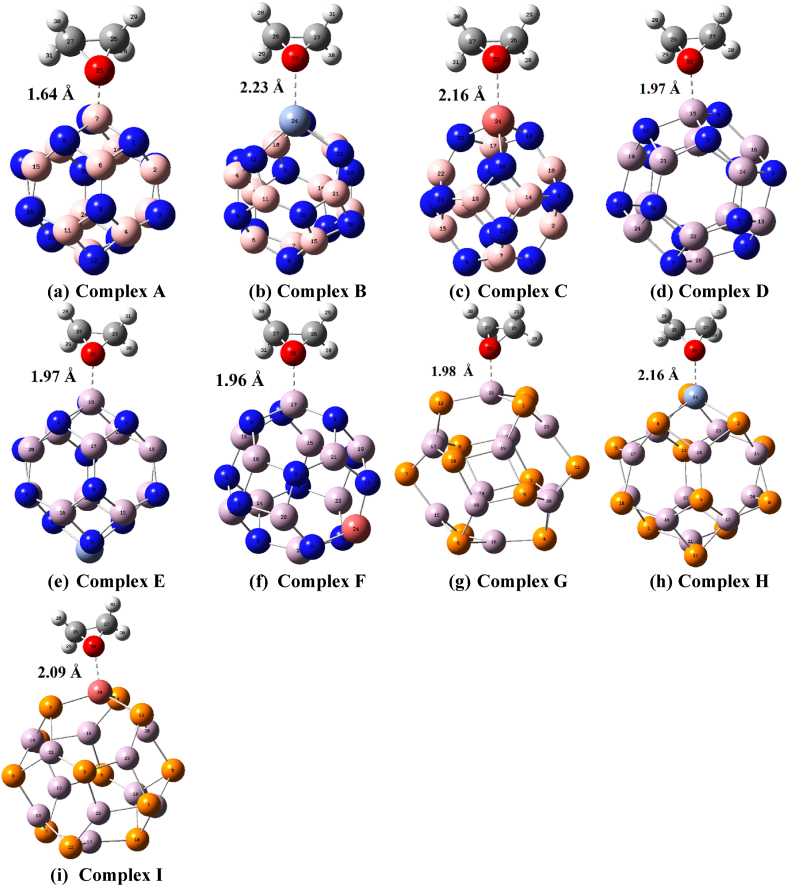
Optimized Geometry of EO adsorbed complexes i.e., (a) Complex A, (b) Complex B, (c) Complex C, (d) Complex D, (e) Complex E, (f) Complex F, (g) Complex G, (h) Complex H, and (i) Complex I. The dashed line denotes the shortest distance between the adsorbate and adsorbents in the ωB97X-D/6-31G(d,p) method.

With the help of thermodynamic and adsorption energy parameters, the adsorption behavior of EO on pure and decorated nanocages is investigated in detail. Based on the adsorption energies between EO and adsorbents, a geometrical orientation of adsorbate(EO) for each adsorbent (nanocage) has been chosen, and these complex nanostructures are mentioned as Complex A, Complex B, and Complex C when EO gas is engaged with pristine BN, Sc-BN, Ti-BN nanocages respectively and Complex D, Complex E, Complex F when EO molecule is engaged with pristine AlN, Sc-AlN, Ti–AlN nanocages respectively whereas Complex G, Complex H, Complex I are labeled when EO molecule is engaged with pristine AlP, Sc-AlP, Ti–AlP nanocages respectively, in both methodologies(see Fig. 2 and S4). Using equation (2) the adsorption energies between EO and pristine nanocages are observed to be around −94.31, −136.05, and −122.47 kJ/mol in ωB97X-D/6-31G(d,p) method. However, in the B3LYP/6-31G(d,p), the adsorption energies among adsorbate and bare nanocages are quite lower than ωB97X-D/6-31G(d,p) (see Table 1 and Table S2). This might be the result of the long-range forces being underestimated by B3LYP functionals. As expected, there would be a significant shift in the adsorption energies after decorating bare nanocages, the highest adsorption for EO molecule is observed to be around −153.58 kJ/mol in ωB97X-D/6-31G(d,p) and −136.43 kJ/mol in B3LYP/6-31G(d,p) method when Sc decorated AlP nanocage is used adsorbent.

**Table 1:** Adsorption Energy, $E_{Ads.}$ in KJ/mole; BSSE corrected adsorption energy, $E_{Ad.\;CP}$ KJ/mole; Dipole Moment, $\mu_{D}$ in Debye; Distance between adsorbate and adsorbent, d in angstrom (Å), minimum and maximum frequencies (*ν*_min_ & *ν*_max_) in cm^−1^, and amount of charge transfer from adsorbate to the adsorbent $Q_{NBO}$ (e unit) in ωB97X-D/6-31G(d,p) method.

Systems	d	$\mu_{D}$	$E_{Ads.}$	$E_{Ad.CP}$	$Q_{NBO}$	$\nu_{\min}$	$\nu_{\max}$
$C_{2}H_{4}O$	–	1.99	–	–	–	838.52	3222.34
Pristine BN	–	0	–	–	–	327.59	1481.53
Complex A	1.64	7.37	−94.31	−78.81	0.268	14.09	3297.59
Sc-BN	–	7.71	–	–	–	199.57	1479.96
Complex B	2.23	10.96	−144.93	−128.53	0.120	19.12	3284.52
Ti-BN	–	3.95	–	–	–	191.39	1482.13
Complex C	2.16	10.25	−137.27	−118.61	0.130	34.56	3282.87
Pristine AlN	–	0	–	–	–	159.62	970.68
Complex D	1.97	6.08	−136.05	−118.38	0.119	25.52	3284.55
Sc-AlN	–	3.67	–	–	–	147.41	973.58
Complex E	1.97	2.85	−133.7	−115.87	0.117	22.56	3287.34
Ti–AlN	–	0.50	–	–	–	144.82	974.71
Complex F	1.96	6.51	−137.72	−119.69	0.118	16.51	3287.59
Pristine AlP	–	0	–	–	–	93.10	573.51
Complex G	1.98	7.52	−122.47	−106.94	0.123	36.14	3289.23
Sc-AlP	–	3.45	–	–	–	79.36	574.73
Complex H	2.16	9.58	−153.58	−138.18	0.245	12.54	3287.94
Ti–AlP	–	0.48	–	–	–	73.74	573.12
Complex I	2.09	8.48	−125.59	−107.82	0.162	18.41	3288.31

This is classified as high chemisorption since the value is significantly more than −96 kJ/mol or −23 kcal/mol [67]. Additionally, the lowest adsorption of the EO molecule is - 94.31 kJ/mol and −70.21 kJ/mol in ωB97X-D/6-31G(d,p) and B3LYP/6-31G(d,p) respectively for pure BN nanocluster, which is referred to as physisorption due to the value being less than −1 eV or −96 kJ/mol. Negative adsorption energy is a characteristic observed in all complex geometries, and it confers an advantageous quality as it signifies a stable interaction, specifically attraction, between the adsorbate and adsorbents [35].

Furthermore, by utilizing the counterpoise methodology with the help of equation (8), the basis set superposition error is eliminated, and as a result of BSSE corrections, the maximum adsorption between EO and Sc-decorated AlP cage decreases to −138.18 kJ/mol and −118.79 kJ/mol whereas the minimum drops to −78.81 kJ/mol and −51.96 kJ/mol in ωB97X-D/6-31G(d,p) and B3LYP/6-31G(d,p) method respectively(see Table 1 and Table S2). Thermodynamic parameters such as the change in enthalpy(ΔH), entropy (ΔS), and Gibbs free energy (ΔG) are analyzed using equations (3a), (3b), and (3c) to get a thorough understanding of the nature of the reaction(see Table 2 and Table S3). ΔH values are observed from −87.48 kJ/mol to −146.87 kJ/mol in ωB97X-D/6-31G(d,p) method and −63.39 kJ/mol to −129.65 kJ/mol in B3LYP/6-31G(d,p) method. The negative sign of enthalpy change implies that the adsorption process of ethylene oxide on the surface of the proposed adsorbents is exothermic [68]. Besides, ΔG values range from −38.20 kJ/mol to −102.73 kJ/mol and −12.92 kJ/mol to −85.84 kJ/mol for ωB97X-D/6-31G(d,p) and B3LYP/6-31G(d,p) method respectively, negative ΔG values implies to a spontaneous reaction among EO and adsorbent nanocages. From the data tables, it appears that the values of ΔH are more negative than the ΔG values, indicating that the entropy should drop as a function of time [69]. On further evaluating ΔS, we discovered that the values of entropy change are negative. So, it was eventually established that entropy decreases as expected. Additionally, vibrational modes are examined to make predictions regarding the structural stability of complex nanostructures and all investigated complex nanostructures exhibit positive vibrational mode which is quite convenient.

**Table 2:** Thermodynamic parameters; Sum of electronic and thermal Enthalpies, H in Hartree(atomic unit); Sum of electronic and thermal Free Energies, G in Hartree(atomic unit); Enthalpy Change, ΔH in kJ/mole; Gibbs Free Energy Change, ΔG in kJ/mole and Entropy Change, ΔS in kJ/mole.kelvin unit in ωB97X-D/6-31G(d,p) method.

Systems	H	G	ΔH	ΔG	ΔS
$C_{2}H_{4}O$	−153.679858	−153.707984			
Pristine BN	−955.739311	−955.784898			
Complex A	−1109.452484	−1109.507430	−87.48	−38.20	−0.17
Sc-BN	−1691.593925	−1691.642472			
Complex B	−1845.326446	−1845.386482	−138.28	−94.59	−0.15
Ti-BN	−1780.289114	−1780.338493			
Complex C	−1934.018472	−1934.078355	−129.97	−83.70	−0.16
Pristine AlN	−3566.651900	−3566.716862			
Complex D	−3720.380831	−3720.455897	−128.85	−81.52	−0.16
Sc-AlN	−4084.929184	−4084.995299			
Complex E	−4238.657239	−4238.733242	−126.55	−78.66	−0.16
Ti–AlN	−4173.635162	−4173.702087			
Complex F	−4327.365095	−4327.442796	−131.48	−85.92	−0.15
Pristine AlP	−7005.790685	−7005.882826			
Complex G	−7159.514433	−7159.616041	−115.24	−66.24	−0.16
Sc-AlP	−7524.039502	−7524.133314			
Complex H	−7677.775295	−7677.880426	−146.87	−102.73	−0.15
Ti–AlP	−7612.739441	−7612.834126			
Complex I	−7766.464788	−7766.570659	−119.44	−74.96	−0.15

### 3.3 MEP, NBO, and charge transfer analysis

To understand the changes in characteristics that occur after doping and ethylene oxide adsorption, the molecular electrostatic potential (MEP) has been studied further. This research establishes a correlation between chemical reactivity and charge distribution. In MEP illustrations, the utilization of the blue color signifies the presence of electron-deficient regions, denoting areas with a positive charge conversely, the red color is employed to represent electron-rich regions, indicating areas with a negative charge. Due to homogeneous charge distribution in pristine nanocages boron, and aluminum atoms are usually colored blue, while nitrogen and phosphorus atoms are colored red(see Fig. 1 and Fig. S1). After decoration, the uniform charge distribution is disrupted since the new atom alters the charge symmetry and this disruption was seen as dipole moment variation which can also be visible from MEP. MEP maps depict that there is a noticeable amount of blue coloration surrounding the positively charged doped atom as well as boron, and aluminum atoms, and a significant amount of red coloration surrounding the oxygen-containing site of the ethylene oxide molecule, strongly implying that the oxygen-containing site of EO molecule can make strong attachment with boron, aluminum, or doped atom of nanocages in complex configurations.

**Fig. 3:**
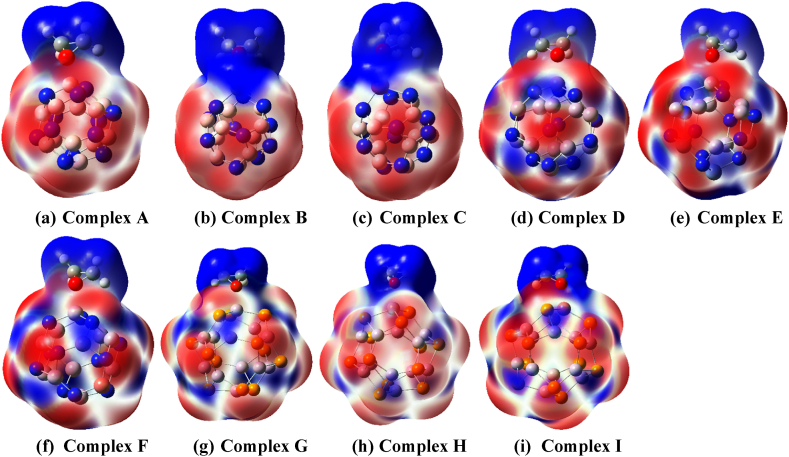
Molecular electrostatic potential (MEP) maps of EO adsorbed complexes' i.e., (a) Complex A, (b) Complex B, (c) Complex C, (d) Complex D, (e) Complex E, (f) Complex F, (g) Complex G, (h) Complex H, and (i) Complex I. The color scheme of the MEP surface (−0.01 a.u. to +0.01 a.u.) shows the electron-rich and electron-deficient areas, respectively in ωB97X-D/6-31G(d,p) method. The MEP surfaces are generated with 0.0004 electron/bohr^3^ iso-value.

The analysis conducted after EO adsorption and associated figures and data finally concluded that the oxygen-containing site of the EO molecule made a strong attachment with the boron, aluminum, and doped atom of nanocages in complex configurations (see Fig. 2, Fig. 3; Figs. S4 and S5).

The amount of charge transfer between adsorbate and adsorbent has been examined by evaluating natural bond orbital (NBO) charges and using NBO charge transfer analysis we observed that charge transfer from EO molecule to adsorbent nanocages in complex scenarios. For instance, in ωB97X-D/6-31G(d,p) method the neutral EO molecule has a near-zero NBO charge (about −0.002 e), which has been altered to +0.268 e in complex A, indicates a significant amount of charge transfer from the adsorbate to the adsorbent. However, the charge transfer simulation can be attained from MEP maps. The neutral EO molecule consists of both electron-rich and lacking sites but the blue color is predominant on ethylene oxide molecules upon EO adsorption which indicates that the EO area is electron-deficient or electropositive after adsorption. This visualization leads to the fact that charge transfer occurs toward adsorbent nanocages after adsorption. The electronegativity analysis (-μ), lends credence to the charge transfer argument as previously mentioned. The electronegativity values of EO molecules have been observed to be significantly lower than adsorbent nanocages in both methods which provides additional evidence in favor of the aforementioned assertion regarding charge transfer(see Table 3 and Table S4).

**Table 3:** HOMO energy, $E_{H}$ in eV; LUMO energy, $E_{L}$ in eV; Energy Gap, $E_{g}$ in eV; Change in energy gap, %Δ $E_{g}$; Fermi Level energy, $E_{F}$ in eV, work function, ɸ in eV and Changes in work function, %ɸ; Chemical Potential, μ; Global Hardness, η; Softness, S; and Global Electrophilicity, ω in ωB97X-D/6-31G(d,p) method.

Systems	$E_{H}$	$E_{L}$	$E_{g}$	%Δ $E_{g}$	$E_{F}$	ɸ	%Δɸ	μ	η	S	ω
$C_{2}H_{4}O$	−9.56	5.10	14.66	–	−2.23	2.23	–	−2.23	7.33	0.07	0.34
Pristine BN	−9.92	1.21	11.13	–	−4.36	4.36	–	−4.36	5.57	0.09	1.71
Complex A	−9.06	1.91	10.97	1.44	−3.58	3.58	17.89	−3.58	5.49	0.091	1.17
Sc-BN	−8.97	−0.64	8.33	25.16	−4.81	4.81	−10.32	−4.81	4.17	0.12	2.77
Complex B	−8.64	0.53	9.17	17.61	−4.06	4.06	6.88	−4.59	4.59	0.11	2.29
Ti-BN	−7.75	−0.31	7.44	33.15	−4.03	4.03	7.56	−4.03	3.72	0.13	2.18
Complex C	−7.20	0.48	7.68	30.99	−3.36	3.36	22.94	−3.36	3.84	0.13	1.47
Pristine AlN	−8.55	−0.64	7.91	–	−4.59	4.59	–	−4.60	3.96	0.126	2.65
Complex D	−8.08	−0.26	7.82	1.14	−4.17	4.17	9.15	−4.17	3.91	0.127	2.22
Sc-AlN	−8.28	−0.45	7.83	1.01	−4.37	4.37	4.79	−4.37	3.92	0.128	2.44
Complex E	−7.89	−0.11	8	−1.14	−3.89	3.89	15.25	−4	4	0.125	2
Ti–AlN	−6.51	−0.56	5.95	24.78	−3.54	3.54	22.88	−3.54	2.98	0.17	2.10
Complex F	−6.18	−0.25	5.93	25.03	−3.22	3.22	29.85	−3.22	2.97	0.168	1.75
Pristine AlP	−8.67	−1.89	6.78	–	−5.28	5.28	–	−5.28	3.39	0.15	4.11
Complex G	−8.25	−1.50	6.75	0.44	−4.88	4.88	7.58	−4.88	3.38	0.147	1.04
Sc-AlP	−8.48	−1.68	6.8	−0.29	−5.08	5.08	3.79	−5.08	3.4	0.147	3.79
Complex H	−8.19	−1.42	6.77	0.15	−4.81	4.81	8.90	−4.81	3.39	0.15	3.41
Ti–AlP	−8.49	−1.91	6.58	2.95	−5.2	5.2	1.52	−5.2	3.29	0.151	4.11
Complex I	−7.48	−1.46	6.02	11.21	−4.47	4.47	15.34	−4.47	3.01	0.17	3.32

### 3.4 Electronic properties

Electronic properties of the gas molecule, adsorbent nanocages, and complex nanostructures, are studied in terms of frontier molecular orbitals (FMO). The frontier molecular orbitals (FMOs) are investigated by analyzing the energies and density of state distribution of gas, adsorbents, and complexes; where associated energies are expressed in terms of the highest occupied molecular orbitals energy ($E_{H}$), lowest unoccupied molecular orbitals energy ($E_{L}$), fermi energy level ($E_{F}$) and energy gap or bandgap ($E_{g}$) (see Table 3 and Table S4).

**Fig. 4:**
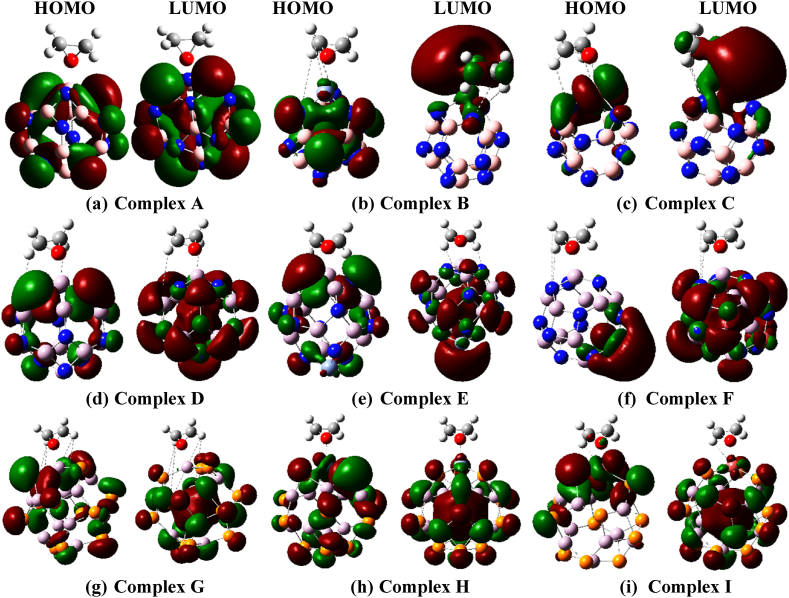
The HOMO and LUMO distribution in the ωB97X-D/6-31G(d,p) method for (a) Complex A, (b) Complex B, (c) Complex C, (d) Complex D, (e) Complex E, (f) Complex F, (g) Complex G, (h) Complex H, and (i) Complex I. The HOMO and LUMO figures are generated with the iso-value of 0.02 electron/bohr^3^.

Fig. 4 and Fig. S6 depict the distribution HOMO and LUMO levels of conjugated geometries, accordingly. Pristine BN nanocage shows a bandgap of 11.13 eV in ωB97X-D/6-31G(d,p) method which decreases up to 33% at metal decoration. Besides, using B3LYP/6-31G(d,p) method, the bandgap reduces up to 55% upon decoration in BN-related nanocages. The AlN nanocage exhibits a bandgap of 7.91 eV and 3.92 eV which reduces up to 25% and 51% following decoration whereas the AlP nanocage exhibits bandgap values of 6.78 eV and 3.38 eV which decreased up to 3% and 11% following decoration, in ωB97X-D/6-31G(d,p) and B3LYP/6-31G(d,p) methods respectively.

The bandgap is altered again by the EO adsorption on the surface of these nanocages. This bandgap alternation caused by doping and EO adsorption may be explained by generating new HOMO and LUMO energy levels where $E_{HOMO}$ decreases and $E_{LUMO}$ increases(or sometimes decreases) hence shrinking the energy gap between the valence and conduction bands. However, due to this phenomenon, the maximum bandgap value is observed for Complex A, whereas Complex F exhibits the smallest upon EO adsorption. All investigated nanostructures have also been studied by equation (9) which shows a relationship between conductivity (σ) and bandgap ($E_{g})$ [70,71].

(9) $$\sigma \propto \exp\left(-E_{g}/2KT\right)$$

where K stands for Boltzmann's constant and T stands for temperature. According to equation (9), a minor change in the Energy gap ($E_{g}$) may result in a substantial change in conductivity. Therefore, if the bandgap falls, conductivity rises, and in the majority of cases, conductivity increases following decoration and adsorption. In addition, the work function of a solid can be defined as the amount of energy required to expel an electron from that solid surface, and the relationship between fermi energy and work function can be stated below [35,72]

(10) $$ɸ=V_{el\left(+\propto \right)}-E_{F}$$

Here, $V_{el\left(+\propto \right)}$ indicates the electrostatic potential of the material surface which can be approximated to zero, resulting in the reduction of equation (10) to ɸ = -$E_{F}$ [73]. However, the drop in work function is found to lower the barrier potential and enhance the current density (j). Based on the findings of this current study, it is evident that the work function values of all systems undergo a decrease subsequent to the introduction of foreign atom decoration and EO adsorption in both methodologies which is quite favorable. The work function (ɸ) and current density (j) may express as follows using the Richardson-Dushman formula [35,74]:

(11) $$j=A\;T^{2}\;\exp\left(\frac{-ɸ}{KT}\right)$$

The Richardson-Dushman constant is denoted by A on the above equation with a unit of A/ $m^{2}$, where K stands for Boltzmann's constant and T for temperature in Kelvin. As a consequence of this equation (11), it can be concluded that a small change in work function may result in a significant shift in current density, and current density rises as the work function lowers.

**Fig. 5:**
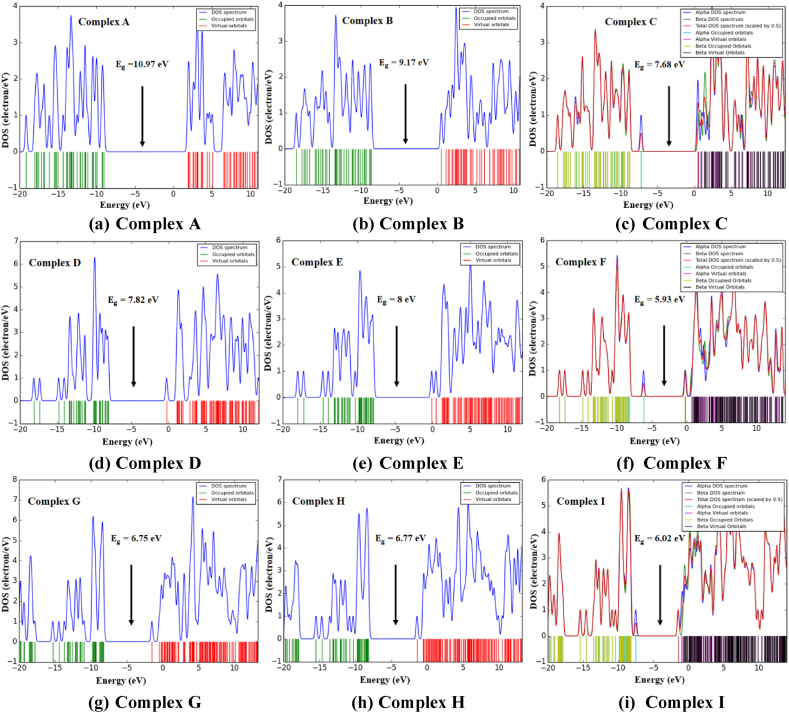
Density of State spectrum illustration of (a) Complex A, (b) Complex B, (c) Complex C, (d) Complex D, (e) Complex E, (f) Complex F, (g) Complex G, (h) Complex H, and (i) Complex I in ωB97X-D/6-31G(d,p) method.

Moreover, by analyzing the density of states (DOS) spectra, the interaction of the EO molecule with adsorbent nanocages as well as the electronic characteristics of conjugated nanostructures can be further validated (see Fig. 5 and Fig. S7). In the field of computational quantum mechanical modeling, DOS spectrums are an excellent tool for displaying energy peaks, bandgap, and fluctuations of the latter. Apart from that, due to ethylene oxide's physisorption on some adsorbents, substantial hybridization in complexes is not evident in DOS spectra, except only in the superposition of EO and nanocages.

### 3.5 Quantum molecular descriptor and recovery mechanism analysis

Global indices like chemical potential (μ), global hardness (η), global softness (S), and electrophilicity (ω) are being investigated by employing equation (4), (5), (6), and (7) to ascertain how decoration and EO adsorption influence the chemical and physical characteristics of nanostructures (see Table 3 and Table S4). The global indices of reactivity are crucial factors because they depict essential information about a molecule's or complex structure's reactivity, physical state, and stability. Ionization potential (I) refers to the amount of energy needed to remove an electron or charge from an isolated atom or molecule whereas electron affinity (A) denotes the inverse of this. Koopmann's theorem asserts that ionization potential(-$E_{H}$) and electron affinity(-$E_{L}$) are directly linked with HOMO energy $(E_{LUMO}$) and LUMO energy $(E_{LUMO}$), respectively [70,75], and values of both ionization potential and electron affinity alter owing to the decoration of pure nanocages with Sc and Ti which are further altered due to EO adsorption. However, a decrease in chemical potential is noticed after the adsorption process takes place. The alterations in global hardness and softness were observed subsequent to the processes of decoration and adsorption of EO. Identical incidents are observed using both ωB97X-D/6-31G(d,p) and B3LYP/6-31G(d,p) methodologies which are quite favorable for gas sensing technology. Both hardness and softness are crucial parameters for analyzing the structural stability of nanostructures, hence their changes have notable effects on gas sensing capability. Global electrophilicity (ω), a measure of a structure's reactivity and fragment's capacity to receive electrons [76], varies significantly after adsorption, implying that the reactivity of systems changes.

To evaluate the efficiency of a sensor, the determination of recovery time is a crucial factor. Investigating the recovery time helps to determine how long it will take for a complex system to release the gas molecule and return to its starting state. The recovery time can be calculated by using the formula below [77,78].

(12) τ=ν0−1exp⁡(−Eads.KT)

Where the recovery time is represented by ***τ***, $\nu_{0}$ is the attempt frequency ∼ $10^{18}\;s^{-1}$ [77,79], K is the Boltzmann constant which is about ∼ 2 *×* $10^{-3}$ kcal mol^−1^ K^−1^ (the recovery time is evaluated by taking BSSE corrected $E_{ads.}$ In Kcal/mol unit), T is the applied temperature. By using this above formula, the sensors show a large recovery period at low temperatures but at high temperatures, they exhibit a quick recovery mechanism. For example, by using equation (12) at 400 K temperature recovery period for Sc-decorated AlP and Sc-decorated BN, nanocages are about 0.84s and 0.047s in ωB97X-D/6-31G(d,p) method whereas at B3LYP/6-31G(d,p) method recovery periods are 0.003s and 0.0001s which is quite preferable. So, by controlling attempt frequencies and temperatures, it is possible to make a quick desorption.

### 3.6 Ultraviolet–visible spectroscopy analysis

**Fig. 6:**
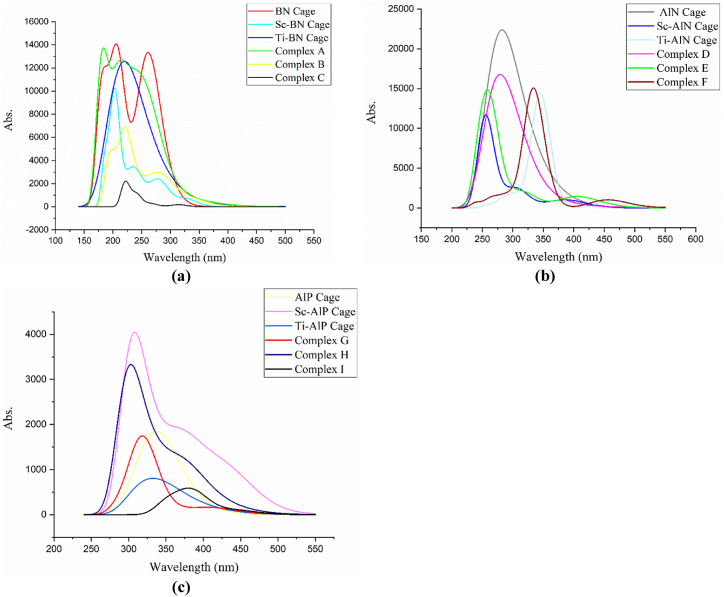
TD-SCF DFT calculated ultraviolet–visible spectroscopy graphs of (a) BN, Sc-BN, Ti-BN nanocages, and their associated complex structures altogether (b) AlN, Sc-AlN, Ti–AlN nanocages, and their associated complex structures (c) AlP, Sc-AlP, Ti–AlP nanocages, and their associated complex structures in ωB97X-D/6-31G(d,p) method.

The ultraviolet–visible spectroscopy analysis of adsorbents and complex structures is conducted by using the time-dependent self-consistent field (TD-SCF), ωB97X-D/6-31G(d,p) and B3LYP/6-31G (d,p) level of theories. Where n state is set at 30 for preventing excessive mixing of frozen cores and valence orbitals in the systems. There are several parameters to consider, including the maximum wavelength of systems ($\lambda_{\max}$), energy linked with maximum wavelength ($E_{\lambda }$) and absorbance (abs.) which are tabulated below (see Table 4 and Table S5). The ultraviolet–visible spectroscopies are plotted using wavelength vs absorbance (see Fig. 6 and Fig. S8).

**Table 4:** Maximum adsorption wavelength ($\lambda_{\max}$) in nm, the energy associated with maximum adsorption wavelength ($E_{\lambda }$) in eV and absorbance of pristine and complex systems. This table has been taken from UV–Vis predicted spectra in ωB97X-D/6-31G(d,p) method.

Systems	Wavelength	Abs.	$E_{\lambda }$
Pristine BN	205.35	14080.78	6.05
Complex A	182.6	13695.27	6.80
Sc-BN	202.36	10247.64	6.14
Complex B	221.24	6900.62	5.62
Ti-BN	220.74	12571.67	5.63
Complex C	221.74	2207.8	5.60
Pristine AlN	282.27	22378.71	4.40
Complex D	278.76	16774.3	4.46
Sc-AlN	254.81	11664.92	4.88
Complex E	259.01	14836.16	4.80
Ti–AlN	345.59	14373.63	3.59
Complex F	333.13	15053.93	3.73
Pristine AlP	337.35	1847.32	3.68
Complex G	318.19	1745.05	3.90
Sc-AlP	308.07	4045.5	4.03
Complex H	303.24	3330.09	4.10
Ti–AlP	330.55	804.82	3.76
Complex I	378.16	588.12	3.29

In TD-SCF, ωB97X-D/6-31G(d,p) methodology, pristine BN, and pristine AlN nanocages exhibited a spectroscopic peak of 205.35 nm and 282.27 nm which has been blue-shifted (resulting in a decrease in the wavelength value) due to Sc decoration and red-shifted(resulting in a rise in the wavelength value) due to Ti decoration whereas in AlP-related nanocages both Sc and Ti decoration causes a blue shift in wavelength. Furthermore, in complex scenarios, Complex A, Complex D, Complex F, Complex G, and Complex H are blue-shifted upon EO adsorption and red-shifted in other instances.

Apart from that, it also found that after EO adsorption on several nanocages, the abs. values altered significantly which indicates the changes in the chemical state of nanostructures after the adsorption of EO. In this way, UV spectra not only describe the nature of excited states but also validate that the occurrences of adsorption and the adsorbents stated above can detect ethylene oxide effectively. However, using TD-SCF, B3LYP/6-31G(d,p) method, the maximum wavelength of all pristine nanocages is blue-shifted after Sc decoration, whereas it is red-shifted after Ti decoration.

It is apparent that the spectroscopic peak undergoes a significant shift in wavelength in the majority of cases upon adsorption of EO. After determining the maximum wavelength, the excited energies ($E_{\lambda })$ are evaluated in further depth, using Planck's Formula [80].

(13) Eλ=hcλmax

Using equation (13), we observed that as the wavelength increases, the energy drops, and when the wavelength lowers, the energy increases since wavelengths and energy are inversely related. On the spectrum of complex nanostructures, the highest excitation energy is determined to be 6.80 eV for Complex A, while the lowest is found to be 3.29 eV for Complex I in ωB97X-D/6-31G(d,p) method(see Table 4). Whereas in B3LYP/6-31G(d,p) method the maximum excitation energy is observed for Complex B which is about 5.04 eV and the minimum is 2.51 eV for Complex I(see Table S5). A significant change in the maximum wavelength and absorbance of complex/adsorbent nanostructures manifests their alternation in conductivity and sensitivity, and this is strong proof of the generation of new energy states when nanocages are decorated or interact with EO molecules [81]. These alternations also demonstrate how doping and ethylene oxide adsorption affect the systems. The ultraviolet–visible spectroscopy describes the potential interactions that have happened in nanostructures after decorating and EO adsorption, and the results are also in great accord with our earlier assertion.

### 3.7 QTAIM investigation

To gain a detailed understanding of a complex system's intermolecular interactions, structural and microelectronic studies may not be adequate. As a consequence, we assessed charge density factors to characterize EO-adsorbed pure and decorated nanoclusters. This is accomplished through the utilization of the AIMALL program, which allowed us to compute QTAIM(Quantum Theory of Atoms in Molecules) calculations and investigate topological characteristics such as the electronic charge density ($\rho_{b}$), laplacian of electronic charge density ($\nabla^{2}\rho_{b}$), total electron energy density ($H_{b}$), and its two components, kinetic electron energy density ($G_{b}$) and potential electron energy density ($V_{b}$), and negative ratio of kinetic and potential electron energy density (−GbVb) for all explored complexes at the bond critical points (BCP), where electrostatic or covalent bonds are formed between two atoms by the transfer or sharing of electron concentrations [82]. When BCPs are present in a complex, it indicates that electron density has been transferred or shared between the atoms, resulting in the formation of covalent or electrostatic bonds within them. The nature of intermolecular contacts can be verified by assessing the values of $\nabla^{2}\rho_{b}$ and $H_{b}$ at BCP [50,83]. When the values of both $\nabla^{2}\rho_{b}$ and $H_{b}$ are negative at BCP, the interactions within adsorbate and adsorbents exhibited as strong covalent; when both $H_{b}$ and $\nabla^{2}\rho_{b}$ are positive the interactions can be referred to as weak electrostatic. Moreover when $\nabla^{2}\rho_{b}$ is greater than zero but $H_{b}$ is smaller than zero the partial covalent and partial electrostatic interaction takes place. It is apparent from Table 5 that in ωB97X-D/6-31G(d,p) both values of $\nabla^{2}\rho_{b}$ and $H_{b}$ is positive in most instances, indicating that the interactions are weak electrostatic except for Complex A(O25–B7), Complex C(O25–Ti24), and Complex E(O25–Al19), where partial covalent and partial electrostatic interactions are dominant. Besides in B3LYP/6-31G(d,p) method, all values of $\nabla^{2}\rho_{b}$ are positive, and nearly identical situation holds for $H_{b}$, means weak electrostatic interaction also occurred here except for Complex A(B19 – O25) which exhibits partial covalent and partial electrostatic interactions. The positive values of $H_{b}$ near the bond critical point correspond to closed-shell interactions, while the negative values correspond to shared-shell interactions [84].

**Table 5:** QTAIM Analysis at the bond critical point (BCP) The topological variables computed include electron densities ($\rho_{b}$) and their laplacian ($\nabla^{2}\rho_{b}$), the local potential electron energy density ($V_{b}$), kinetic electron density ($G_{b}$), the negative ratio of potential and kinetic electron energy density (−GbVb) and the total electron energy densities ($H_{b}$) in the atomic unit in ωB97X-D/6-31G(d,p) method.

Systems	Contact Gas - Adsorbent	$\rho_{b}$	$\nabla^{2}\rho_{b}$	$G_{b}$	$V_{b}$	$H_{b}$	−GbVb
Complex A	O25–B7	0.093418	0.332872	0.137665	−0.192112	−0.054447	0.716587
Complex B	O25-Sc24	0.047841	0.252631	0.057246	−0.051335	0.005911	1.115145
	H30–N17	0.009543	0.031787	0.006797	−0.005647	0.00115	1.203647
Complex C	O25–Ti24	0.055216	0.301209	0.053733	−0.062163	−0.00843	0.864388
	H31–N23	0.008777	0.028207	0.006017	−0.004982	0.001035	1.207747
	H28–N18	0.008777	0.028207	0.006017	−0.004982	0.001035	1.207747
Complex D	O25–Al15	0.048078	0.316066	0.073211	−0.067406	0.005805	1.086119
	H30–N6	0.012810	0.041066	0.009064	−0.007862	0.001202	1.152887
Complex E	O25–Al19	0.011922	0.039988	0.008660	−0.066889	−0.058229	0.129468
	H29–N7	0.047800	0.313587	0.072643	−0.007322	0.065321	9.921196
Complex F	O25–Al17	0.046561	0.323805	0.074708	−0.068443	0.006265	1.091536
	H31–N6	0.012406	0.039810	0.008790	−0.007627	0.001163	1.152484
Complex G	O25–Al22	0.046781	0.292608	0.068861	−0.064571	0.00429	1.066438
	H28–P9	0.007581	0.025434	0.005013	−0.003667	0.001346	1.367057
Complex H	O25-Sc24	0.055403	0.297534	0.068059	−0.061735	0.006324	1.102437
Complex I	O25–Ti24	0.061530	0.364856	0.083673	−0.076131	0.007542	1.099066
	H30–P4	0.007225	0.022899	0.004520	−0.003315	0.001205	1.363499

So, it may be appropriate to state that weak electrostatic interactions are prevailing for the vast majority of situations that have been explored so far, based on these results. Meanwhile, the negative ratio of kinetic and potential electron energy densities extensively elucidates the nature of the interactions, as −GbVb > 1 asserts the electrostatic nature of the interactions while −GbVb < 1 shows the nature of a covalent one [85]. From Table 5 and Table S6, in most of the BCPs, the values of the negative ratio of kinetic and potential electron energy densities are found to be positive and greater than 1 so we may thus conclude that the electrostatic nature between EO and nanocages is dominating in both methodologies for complex systems.

**Fig. 7:**
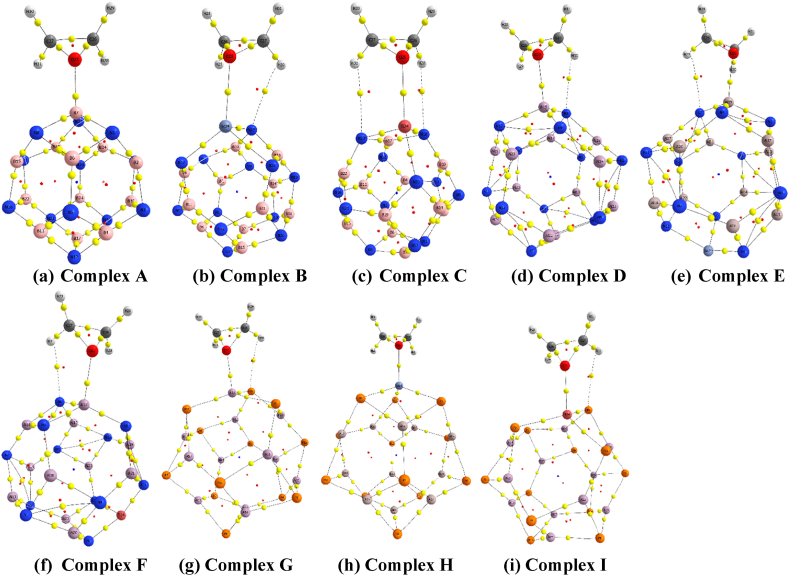
The molecular graphs of (a) Complex A, (b) Complex B, (c) Complex C, (d) Complex D, (e) Complex E, (f) Complex F, (g) Complex G, (h) Complex H, and (i) Complex I at their bond critical sites in the ωB97X-D/6-31G(d,p) method.

Fig. 7 and Fig. S9 illustrate the corresponding images which are showing BCP from QTAIM analysis in ωB97X-D/6-31G(d,p) and B3LYP/6-31G(d,p) methods respectively(the yellow points between bonds represent the BCP).

## 4 Conclusion

This study is carried out to investigate the adsorption characteristics and sensitivity of pristine as well as Sc and Ti-decorated BN, AlN, and AlP nanoclusters towards ethylene oxide (EO) gas by employing density functional theory at ωB97X-D/6-31G(d,p) and B3LYP/6-31G(d,p) level. Possible interactions between ethylene oxide (EO) and nanocages are analyzed in terms of several parameters. Depending on the electrical, physical, and structural characteristics of proposed nanocages, ethylene oxide molecule exhibits a variety of distinct interactions with them. All of the adsorbents exhibit comparatively favorable adsorption energy towards EO molecule, as well as a substantial variance in dipole moments across the board, but Sc-decorated AlP nanocage and Sc-decorated BN nanocages show outstanding adsorption towards EO. This phenomenon suggests that these two nanocages can adsorb ethylene oxide better than others. In addition, the adsorption process appears to be exothermic, and greater spontaneity in the interaction between gas and Sc-decorated BN and AlP nanocages is predicted by negative ΔG values. The charge transfer analysis suggests that in complex systems, ethylene oxide serves as a donor and nanocages as an acceptor of charges. Frontier molecular orbitals analysis reveals changes in electronic characteristics upon decoration and EO adsorption. DOS, UV-spectra, and Quantum molecular descriptors of systems provide information about the generation of new energy states, changes in chemical states, excitation states, conductivity, and sensitivity. QTAIM analysis demonstrates that weak electrostatic interactions are predominant in most systems. The recovery times for systems are in the microsecond range at high temperatures, predicting the reusability of the sensor devices. Finally, based on the findings of our theoretical estimations above, Sc-decorated BN and AlP nanocages are found to be the most promising candidate for EO adsorption and sensing.

## Funding

There was no funding assistance for this research from any other organization or source.

## Author contribution statement

Palash Dhali: Conceived and designed the experiments; Performed the experiments; Analyzed and interpreted the data; Wrote the paper.

Adita Afrin Oishi: Performed the experiments; Analyzed and interpreted the data; Contributed reagents, materials, analysis tools or data.

Antu Das: Md. Mehade Hasan: Analyzed and interpreted the data; Contributed reagents, materials, analysis tools or data.

Md. Mehade Hasan: Supervision.

Md. Rakib Hossain: Farid Ahmed: Debashis Roy: Contributed reagents, materials, analysis tools or data.

## Data availability statement

Data will be made available on request.

## Declaration of competing interest

The authors declare that they have no known competing financial interests or personal relationships that could have appeared to influence the work reported in this paper.
