# Supplementary material for: A DFT and QTAIM insight into ethylene oxide adsorption on the surfaces of pure and metal-decorated inorganic fullerene-like nanoclusters
Source: Heliyon. 2023 Aug 25;9(9):e19407. doi: 10.1016/j.heliyon.2023.e19407 (PMC10558507; doi:10.1016/j.heliyon.2023.e19407)
Supplement: Supplementary file 1 — Multimedia component 1 [file mmc1.docx]

**Supplementary Files**

**A DFT and QTAIM Insight into Ethylene Oxide Adsorption on the Surfaces of Pure and Metal-decorated Inorganic Fullerene-like Nanoclusters**

Palash Dhali^a^, Adita Afrin Oishi^a^ Antu Das^a^, Md. Rakib Hossain^b^, Farid Ahmed^c^, Debashis Roy^a^, Md. Mehade Hasan^a^

^a^Department of Physics, Jashore University of Science and Technology, Jashore - 7408, Bangladesh.

^b^Department of Physics, Bangabandhu Sheikh Mujibur Rahman Science and Technology University, Gopalganj-8100, Bangladesh.

^c^Department of Physics, Jahangirnagar University, Savar, Dhaka-1342, Bangladesh.

Corresponding Author: Md. Mehade Hasan, [mehade.36@gmail.com](mailto:mehade.36@gmail.com), [mehade@just.edu.bd](mailto:mehade@just.edu.bd)

| **Optimized Geometry** | **MEP** | **Optimized Geometry** | **MEP** |
| --- | --- | --- | --- |
| 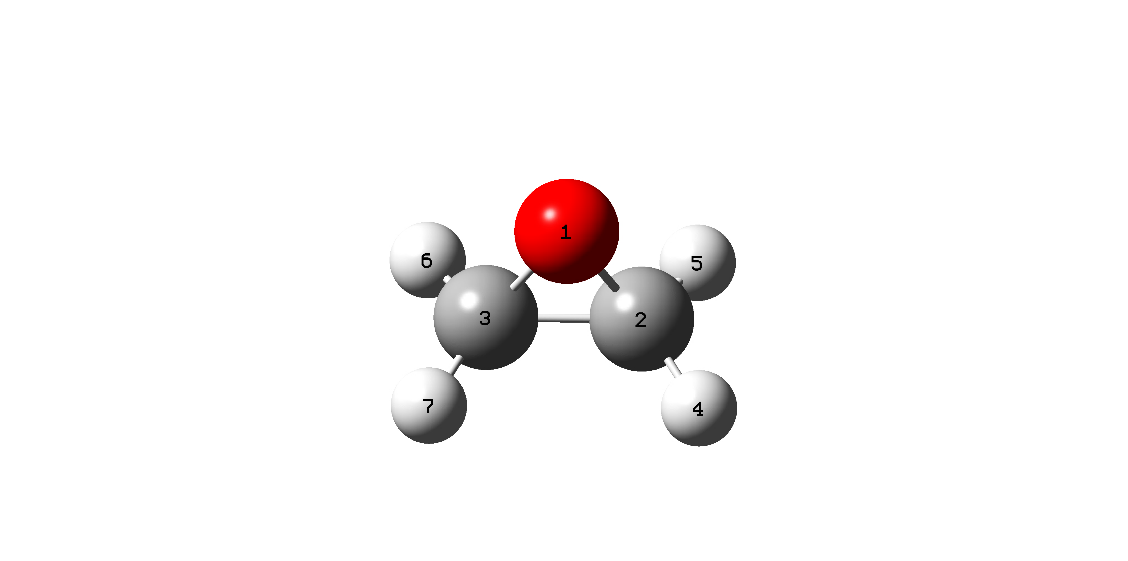 | 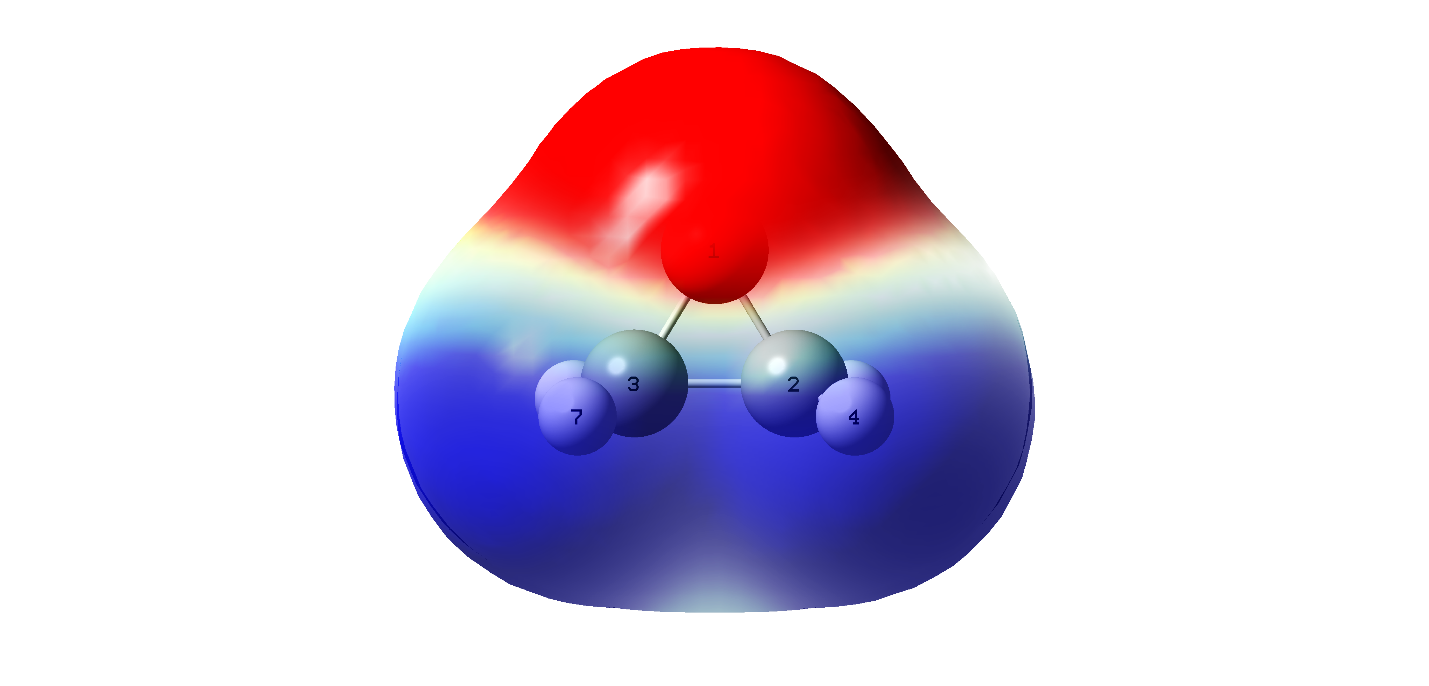 | 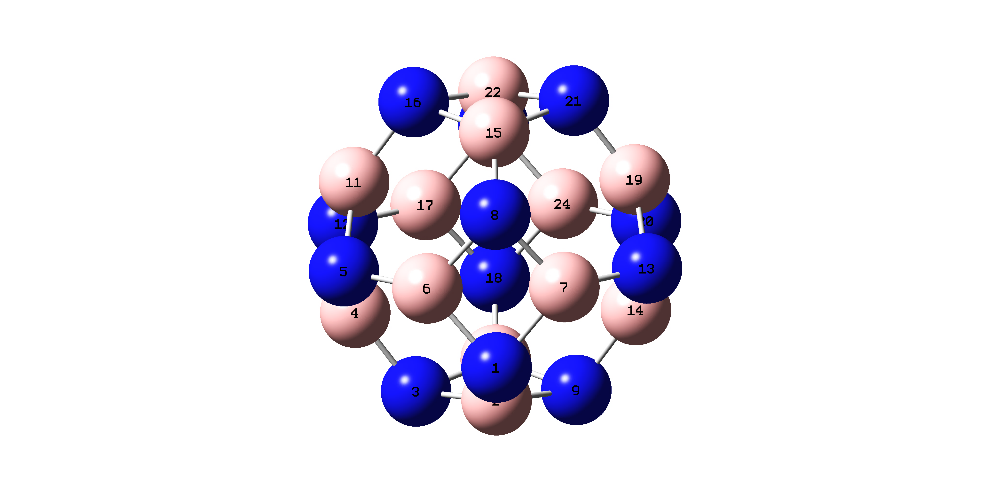 | 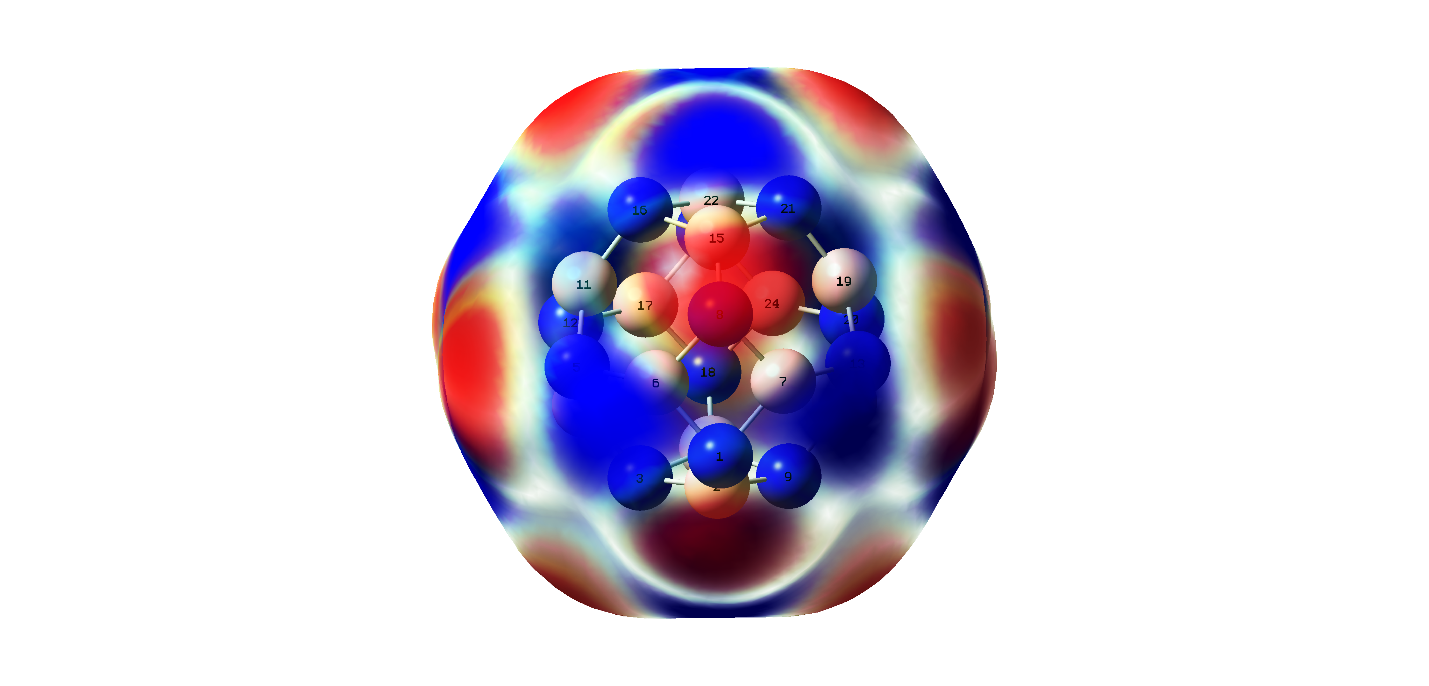 |
| 1. **Ethylene Oxide** | | 1. **BN Nanocage** | |
| 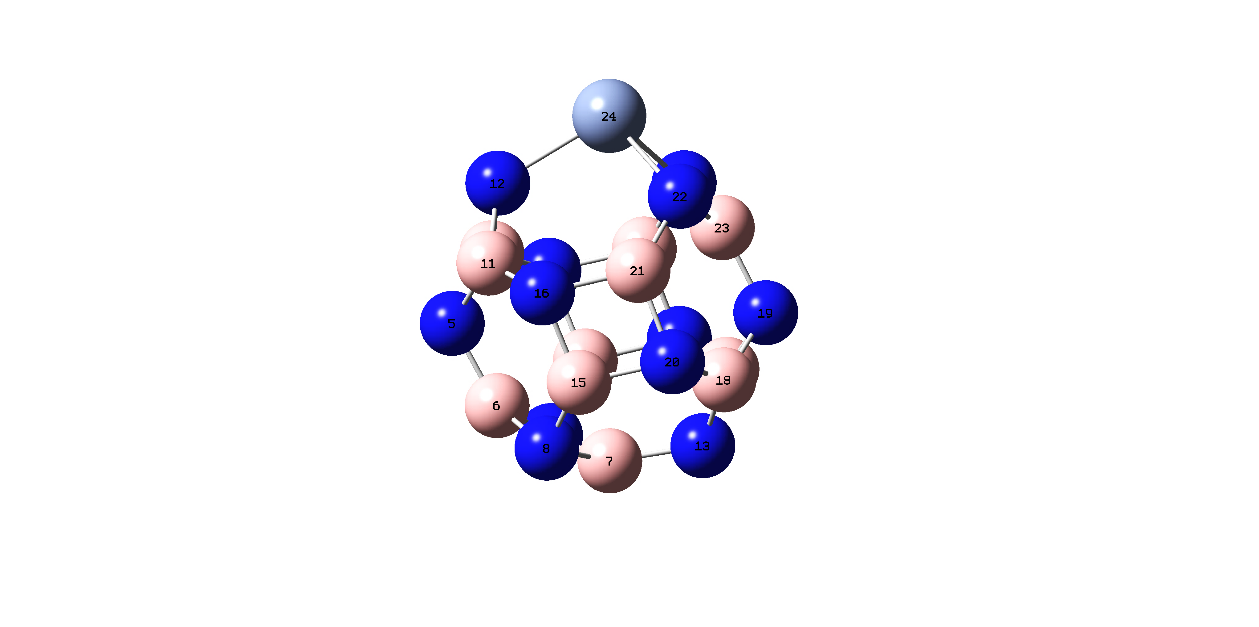 | 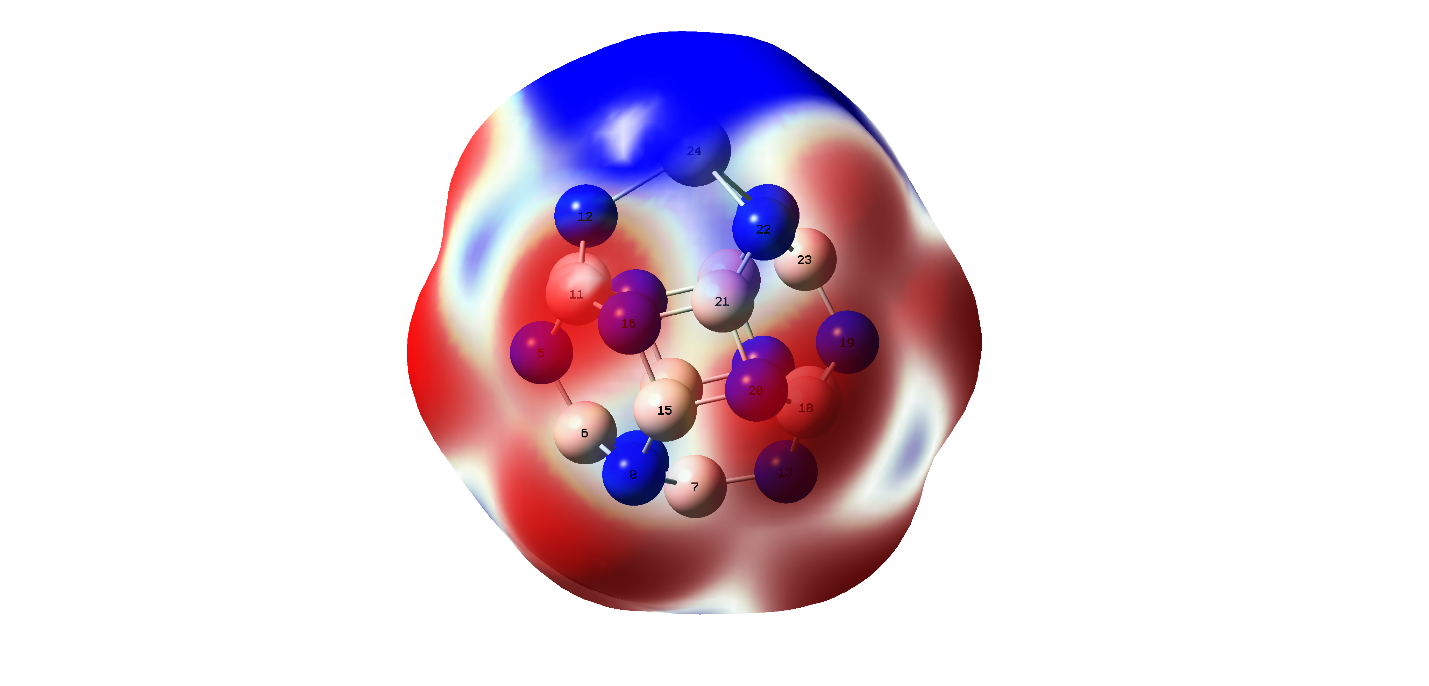 | 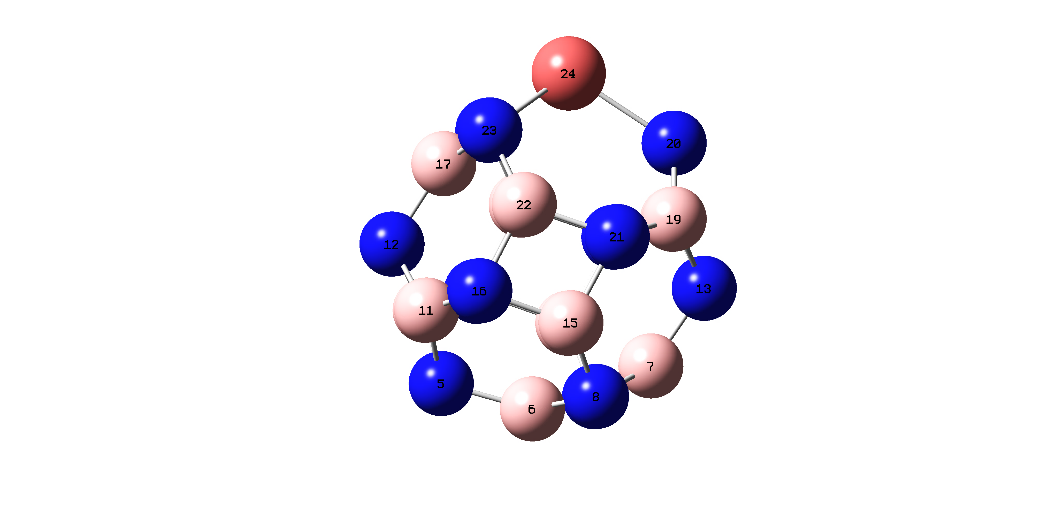 | 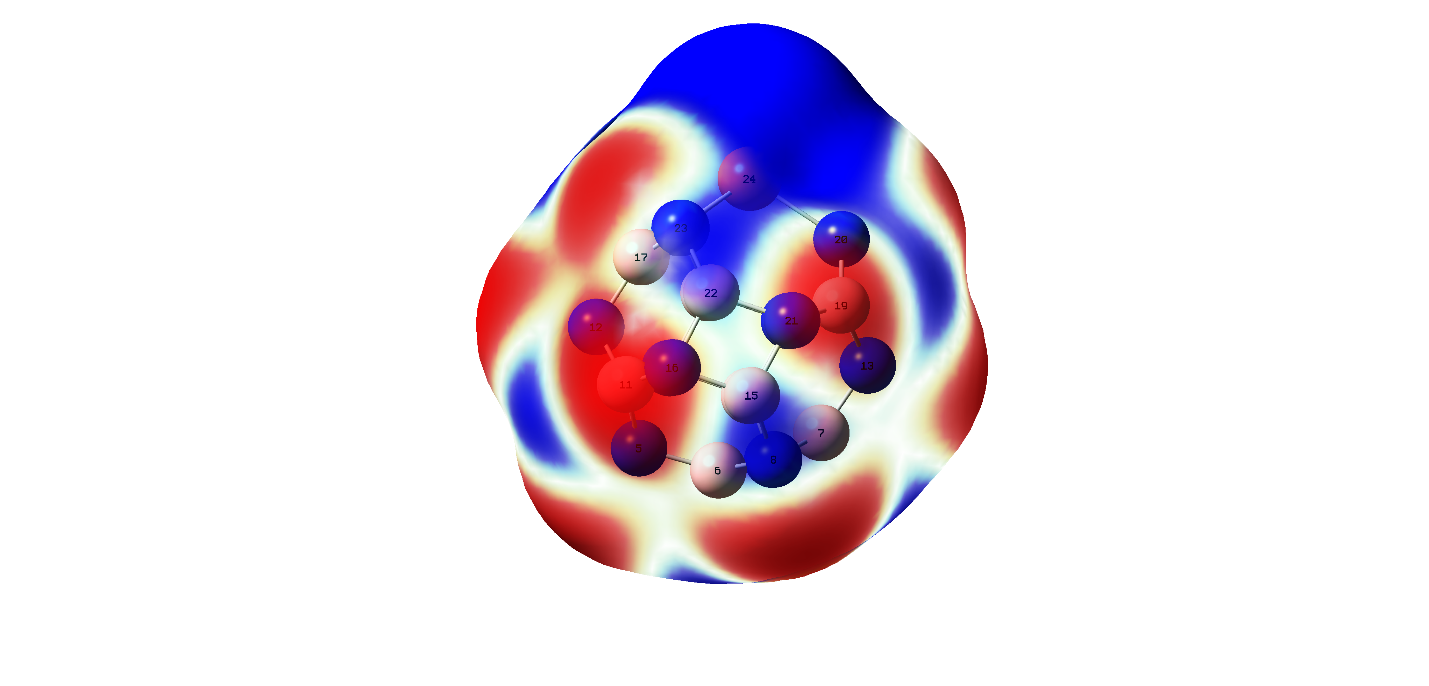 |
| 1. **Sc-BN Nanocage** | | 1. **Ti-BN Nanocage** | |
| 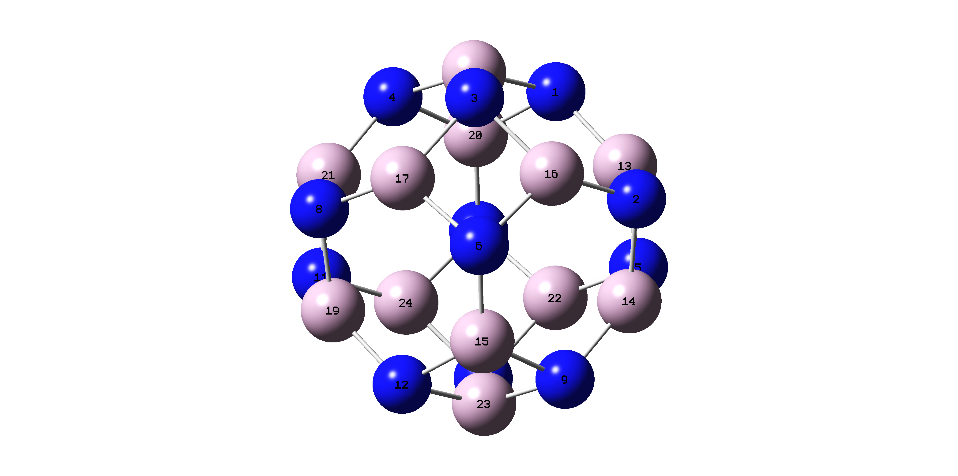 | 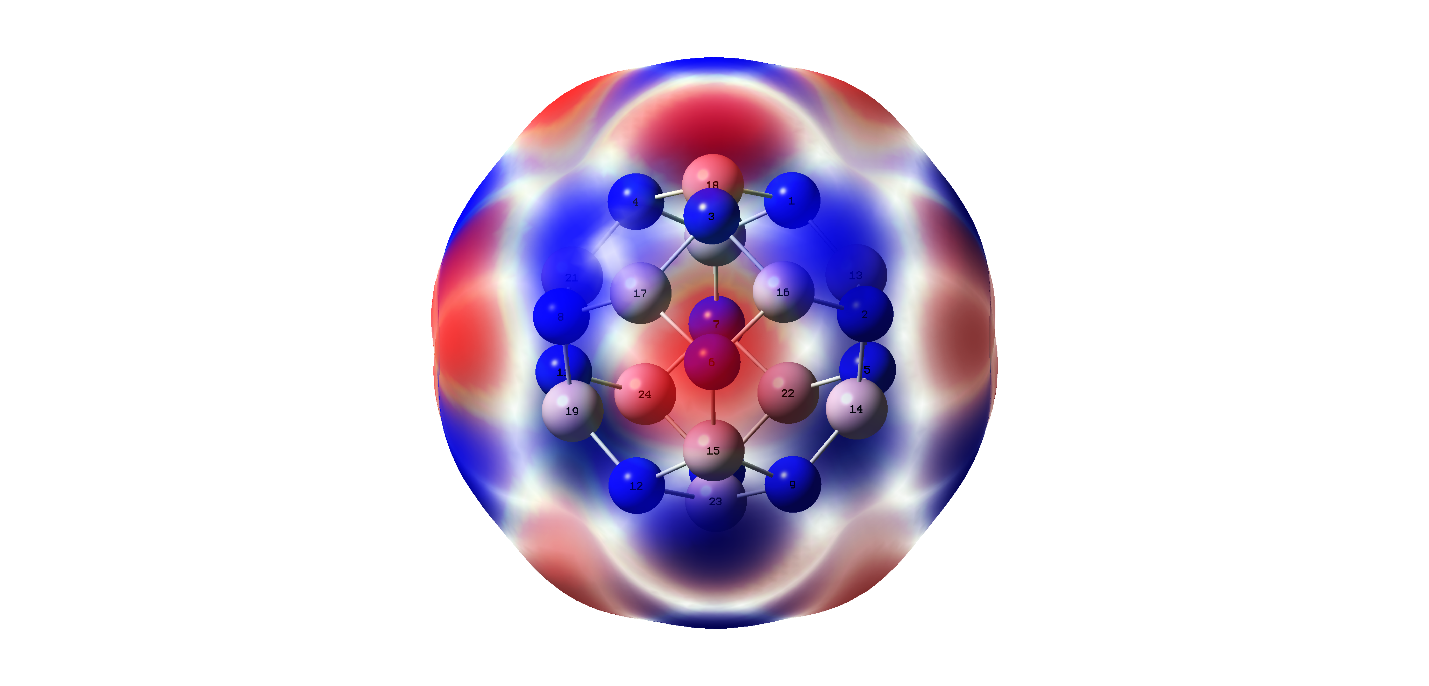 | 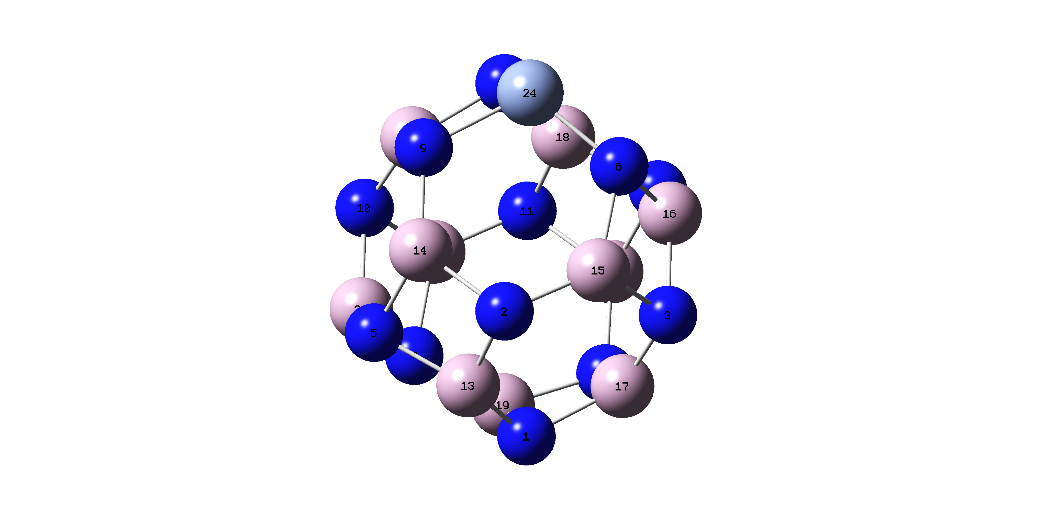 | 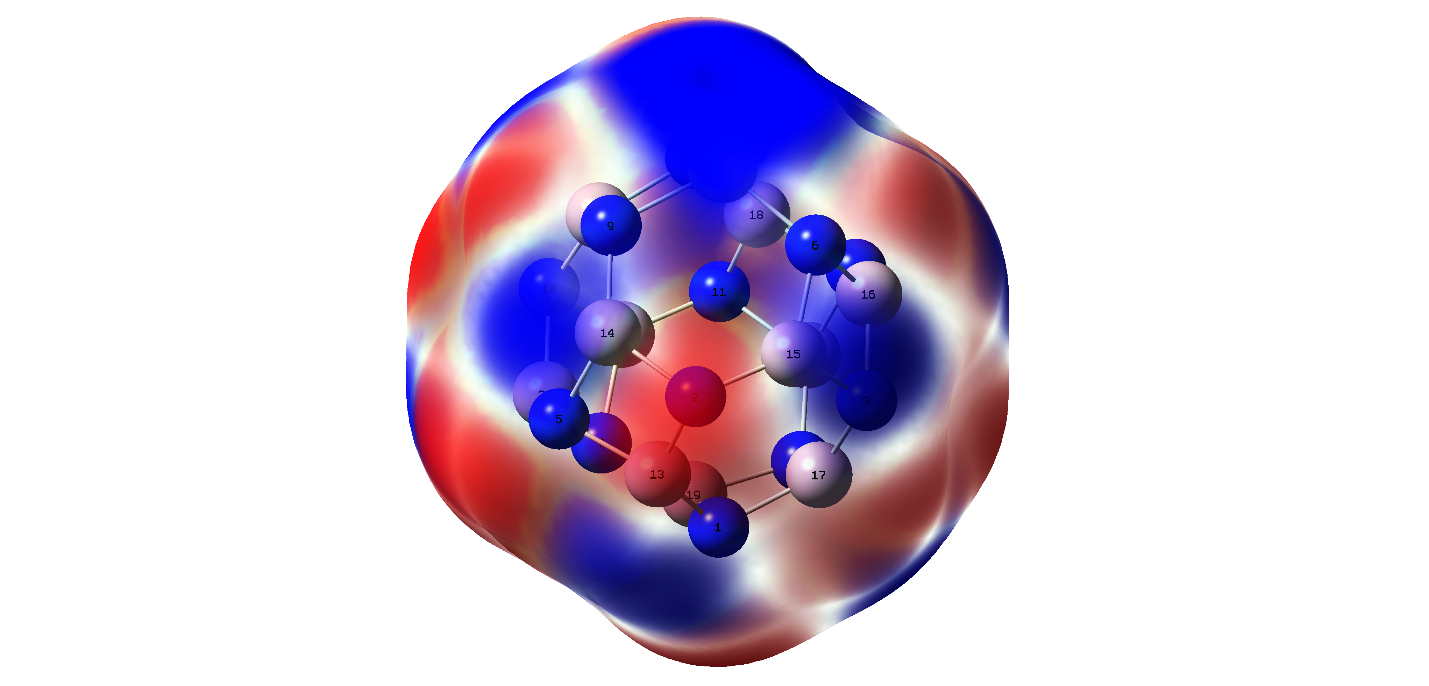 |
| 1. **AlN Nanocage** | | 1. **Sc-AlN Nanocage** | |
| 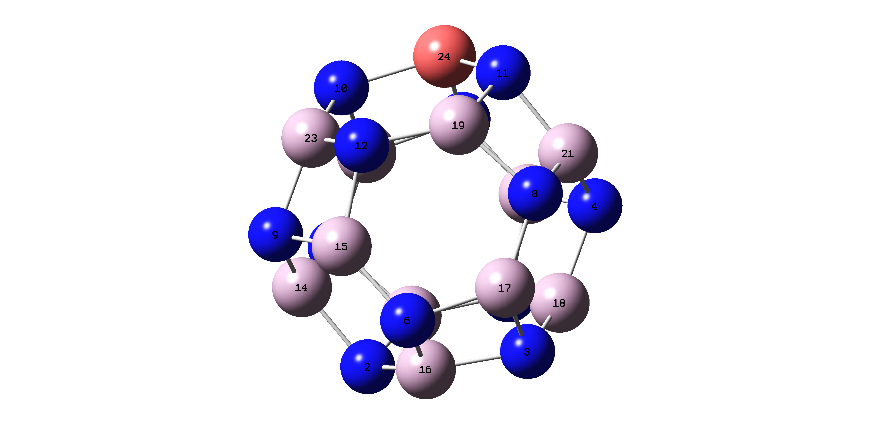 | 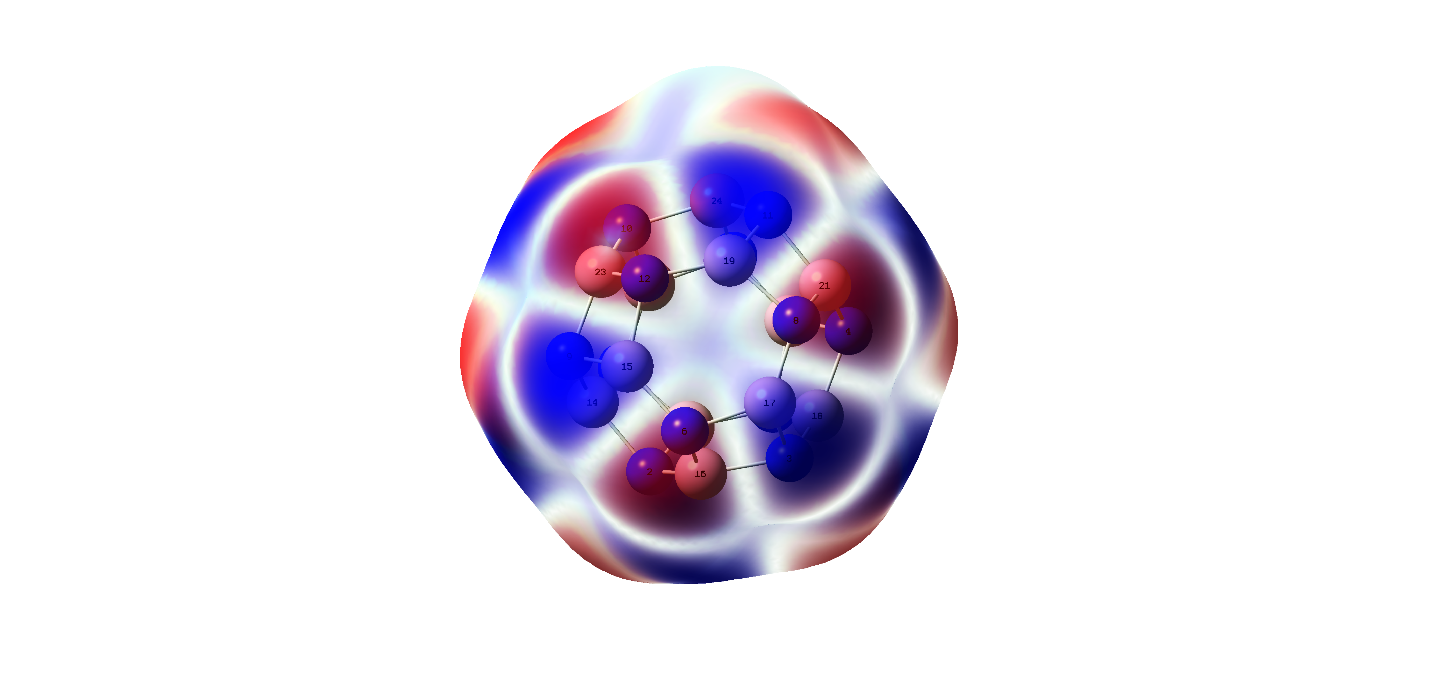 | 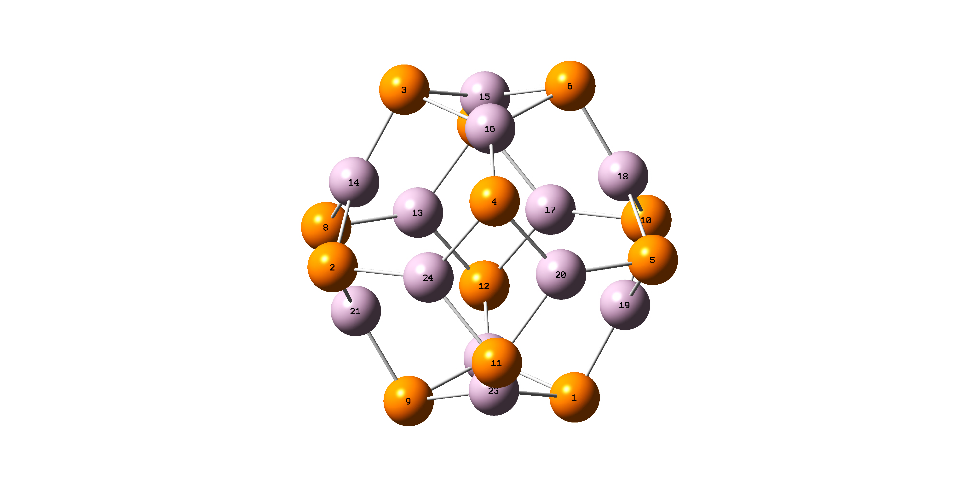 | 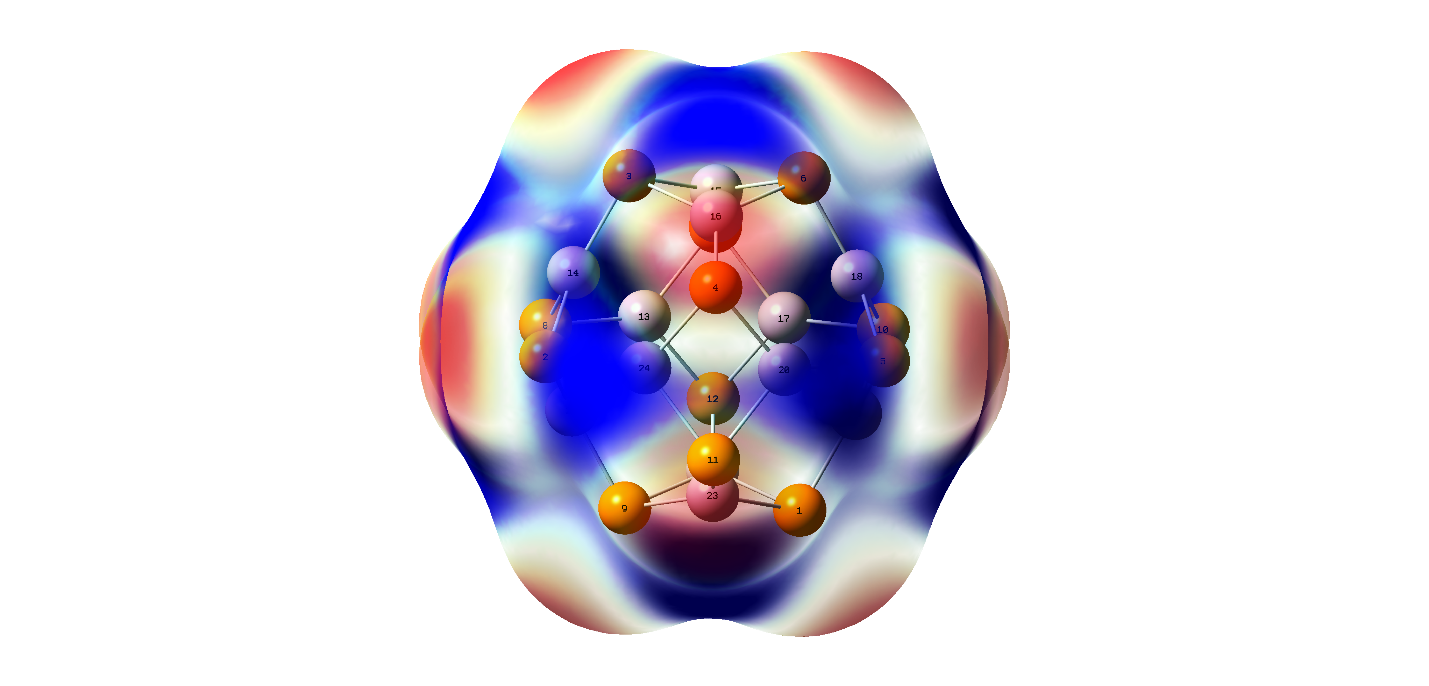 |
| 1. **Ti-AlN Nanocage** | | 1. **AlP Nanocage** | |
| 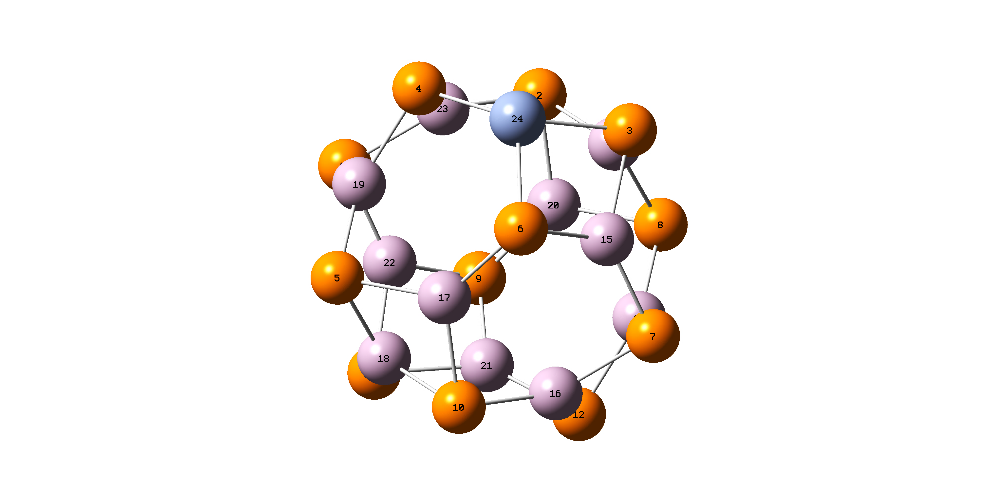 | 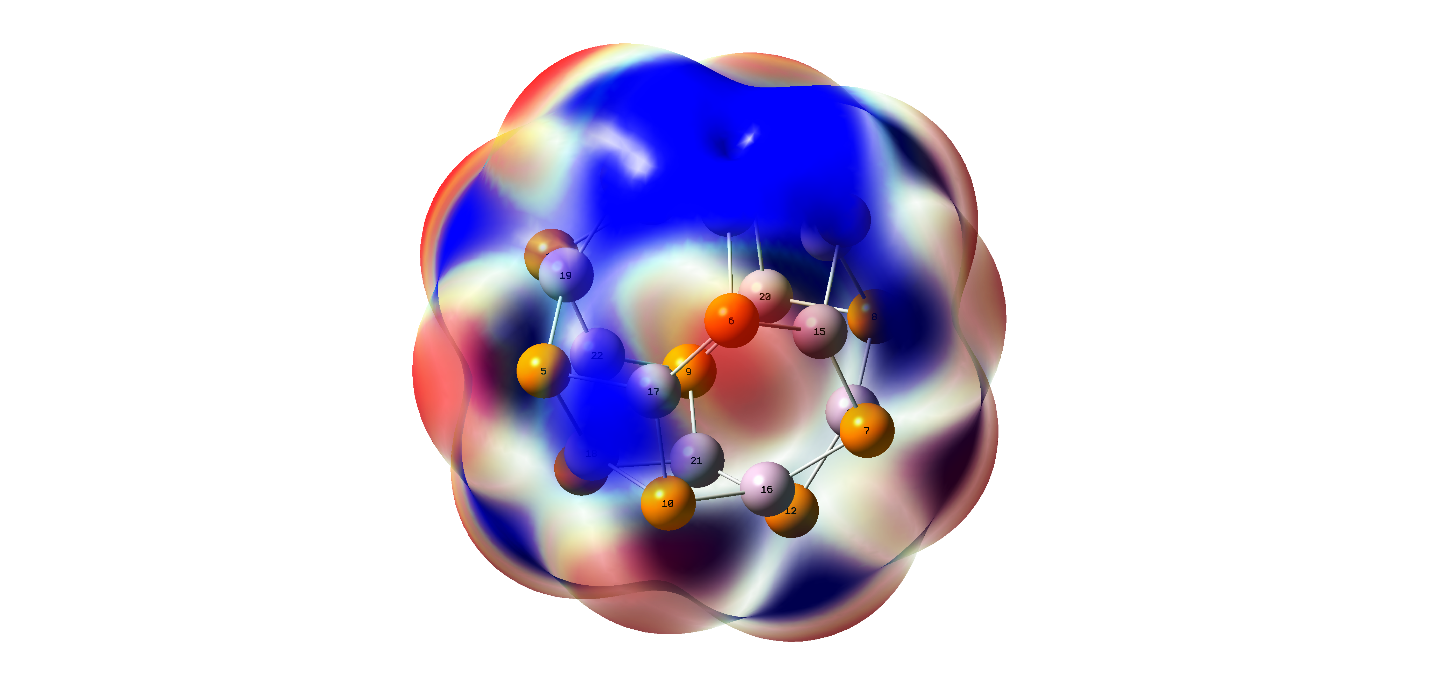 | 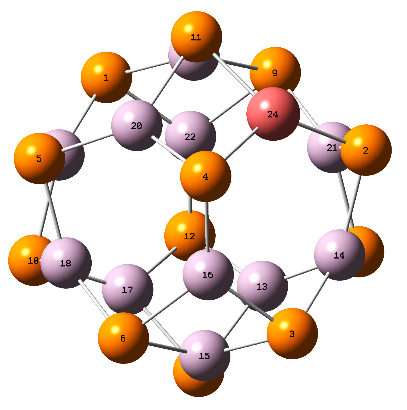 | 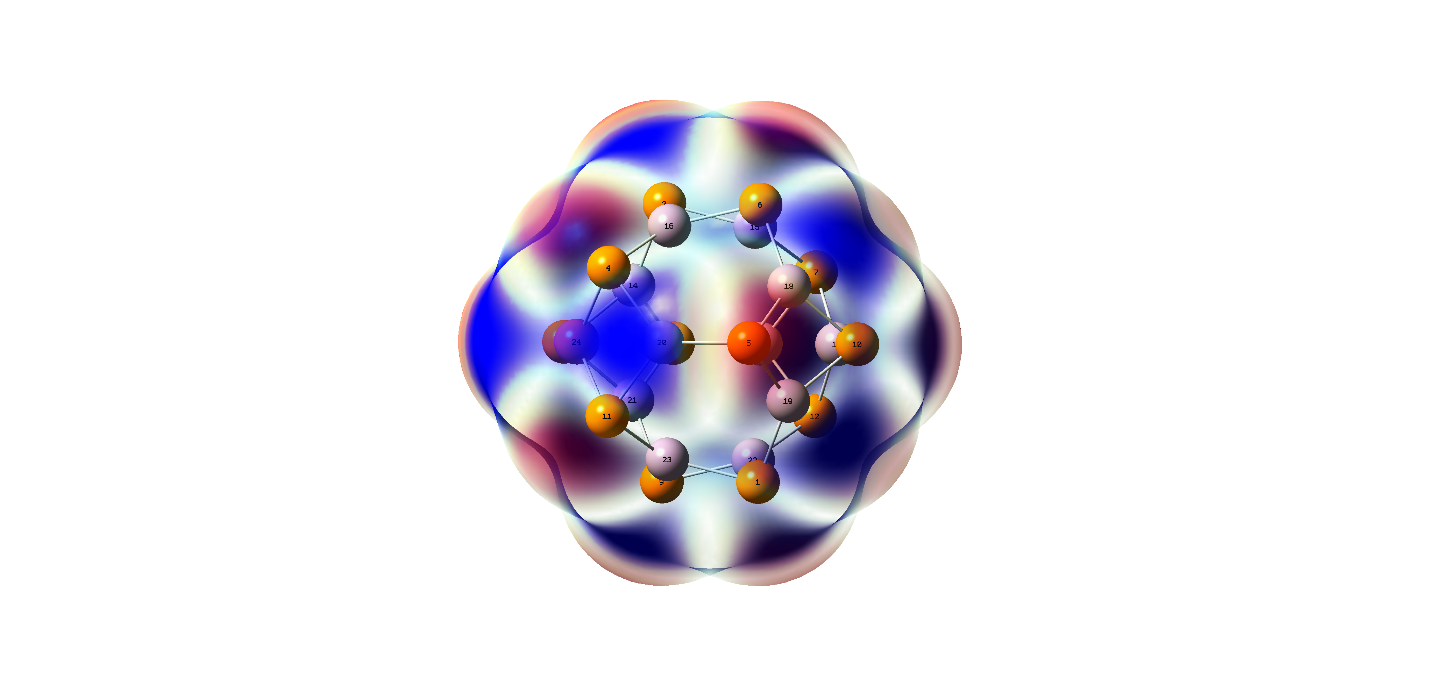 |
| 1. **Sc-AlP Nanocage** | | 1. **Ti-AlP Nanocage** | |

| **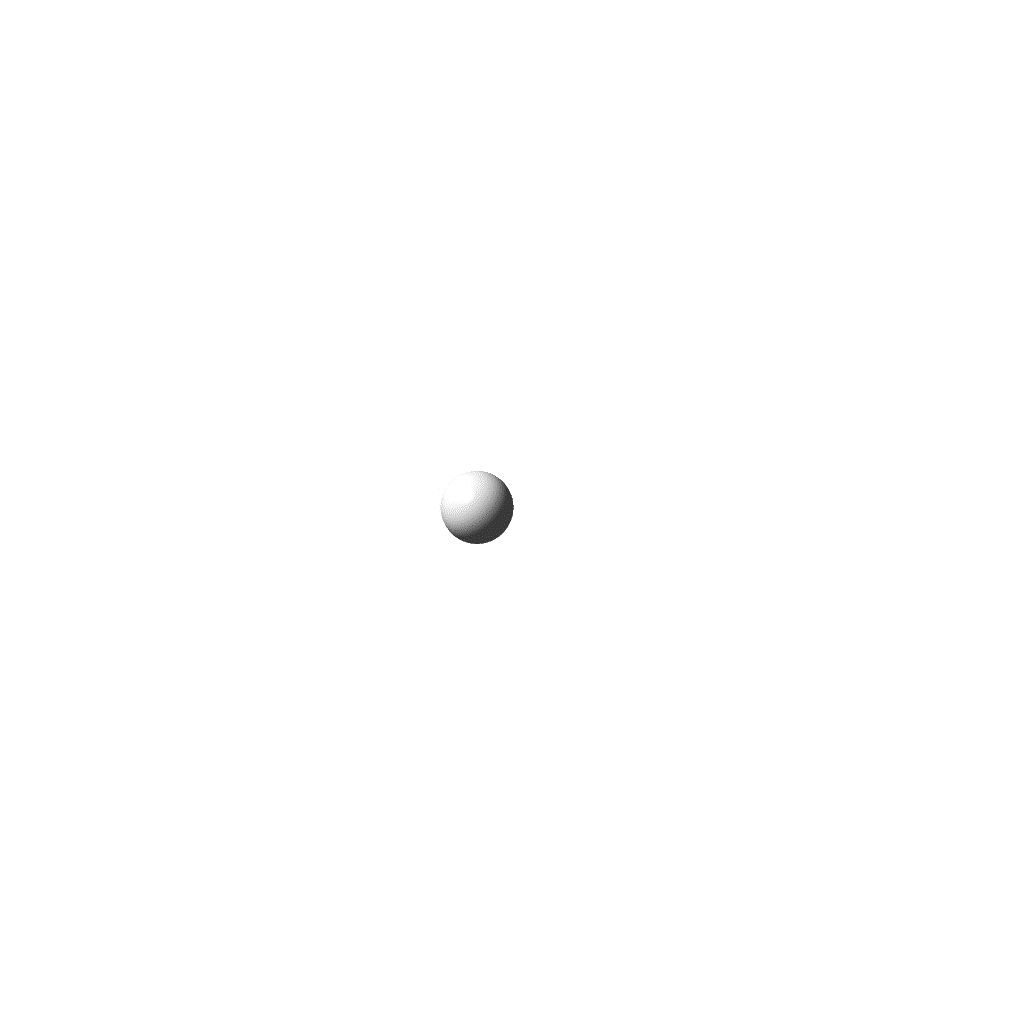** | **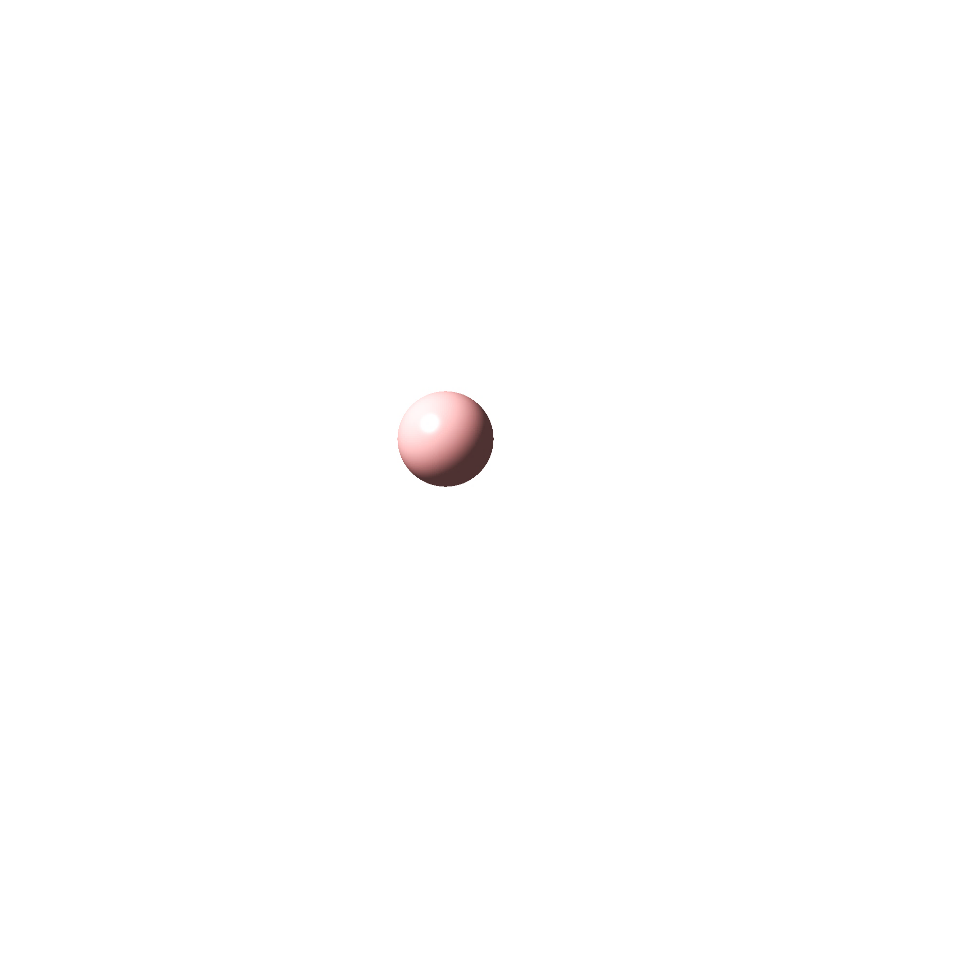** | **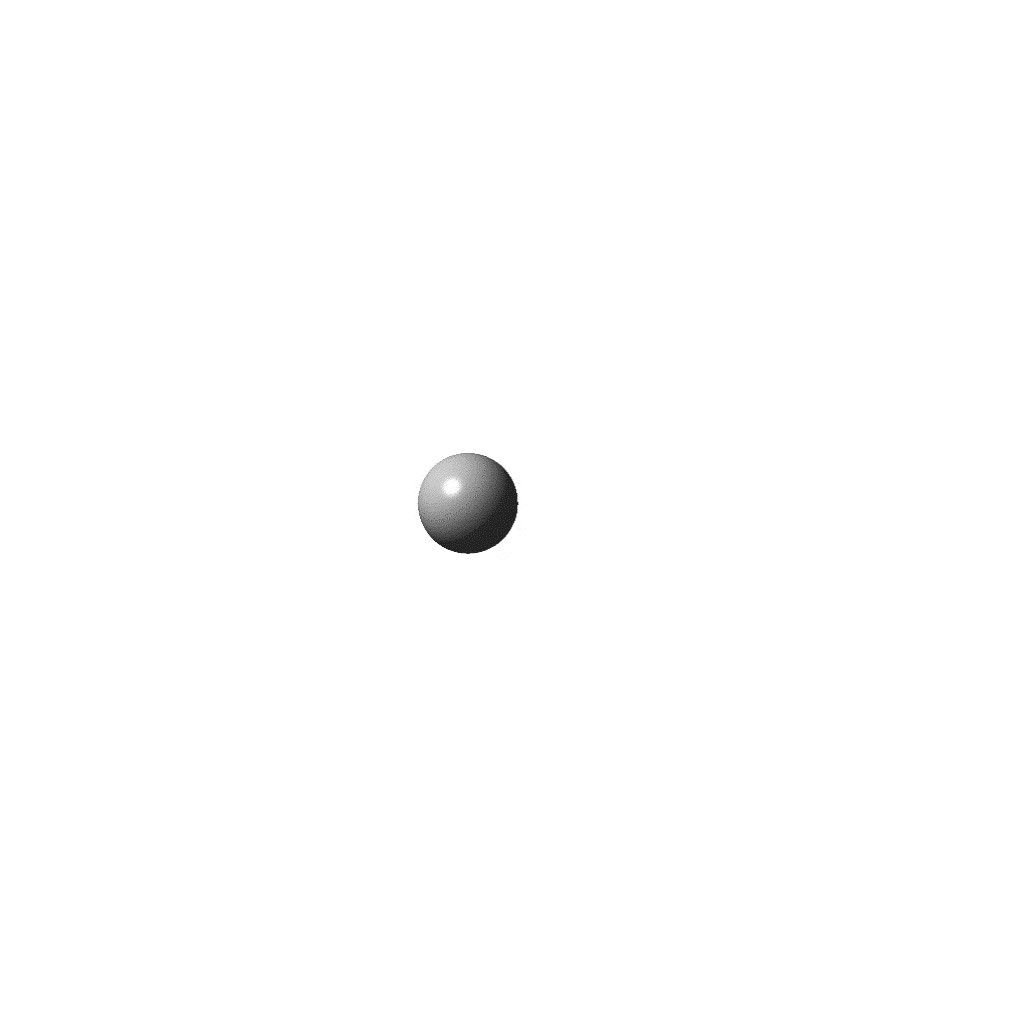** | **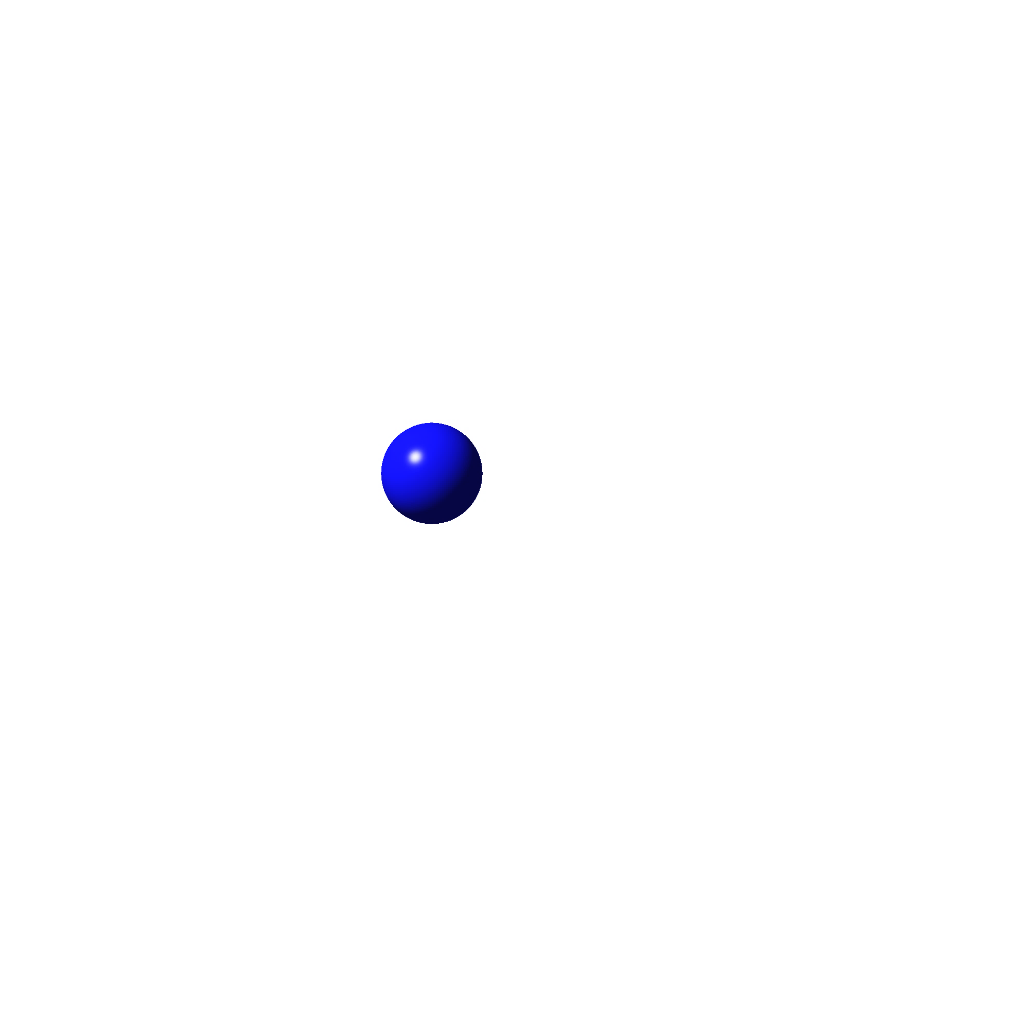** | **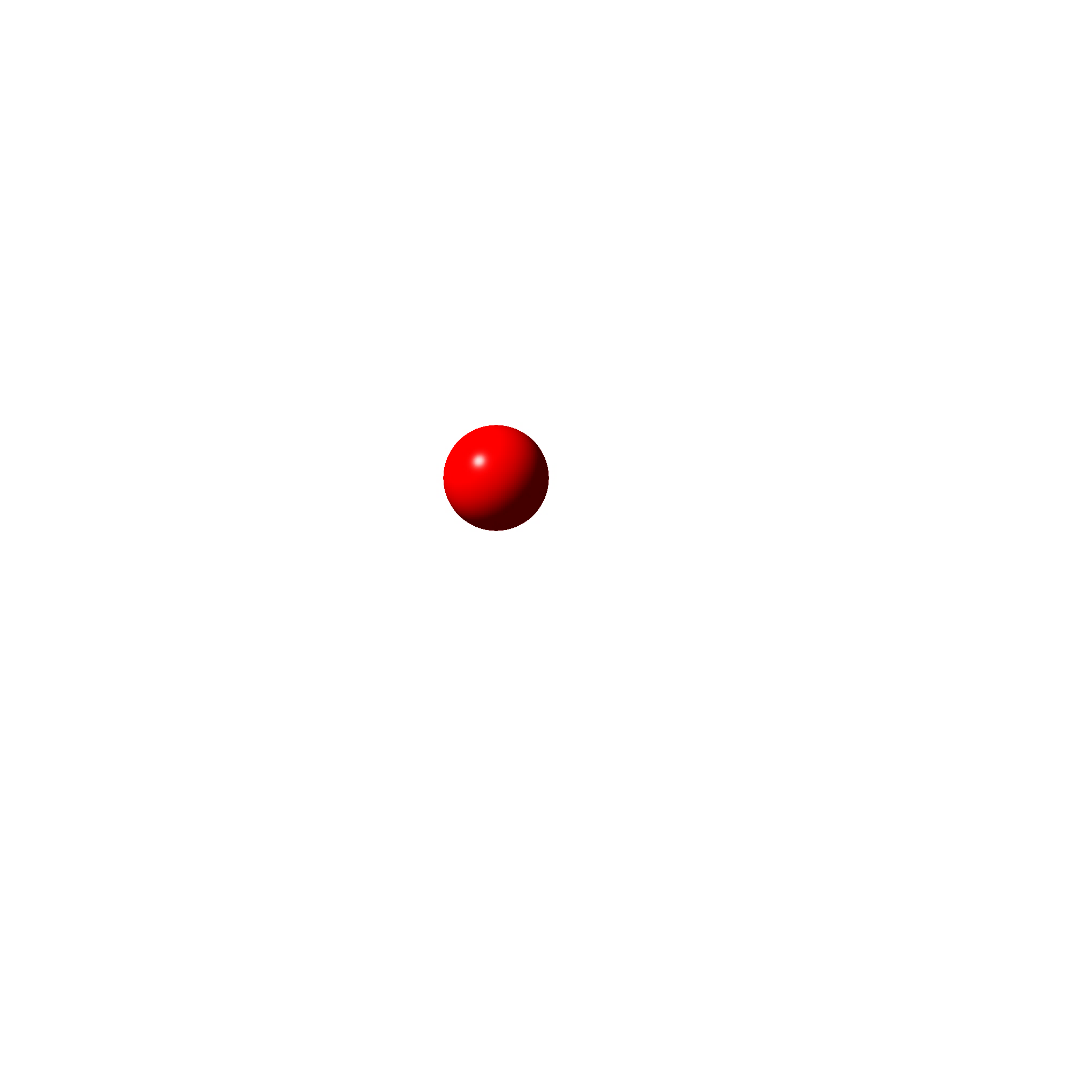** | **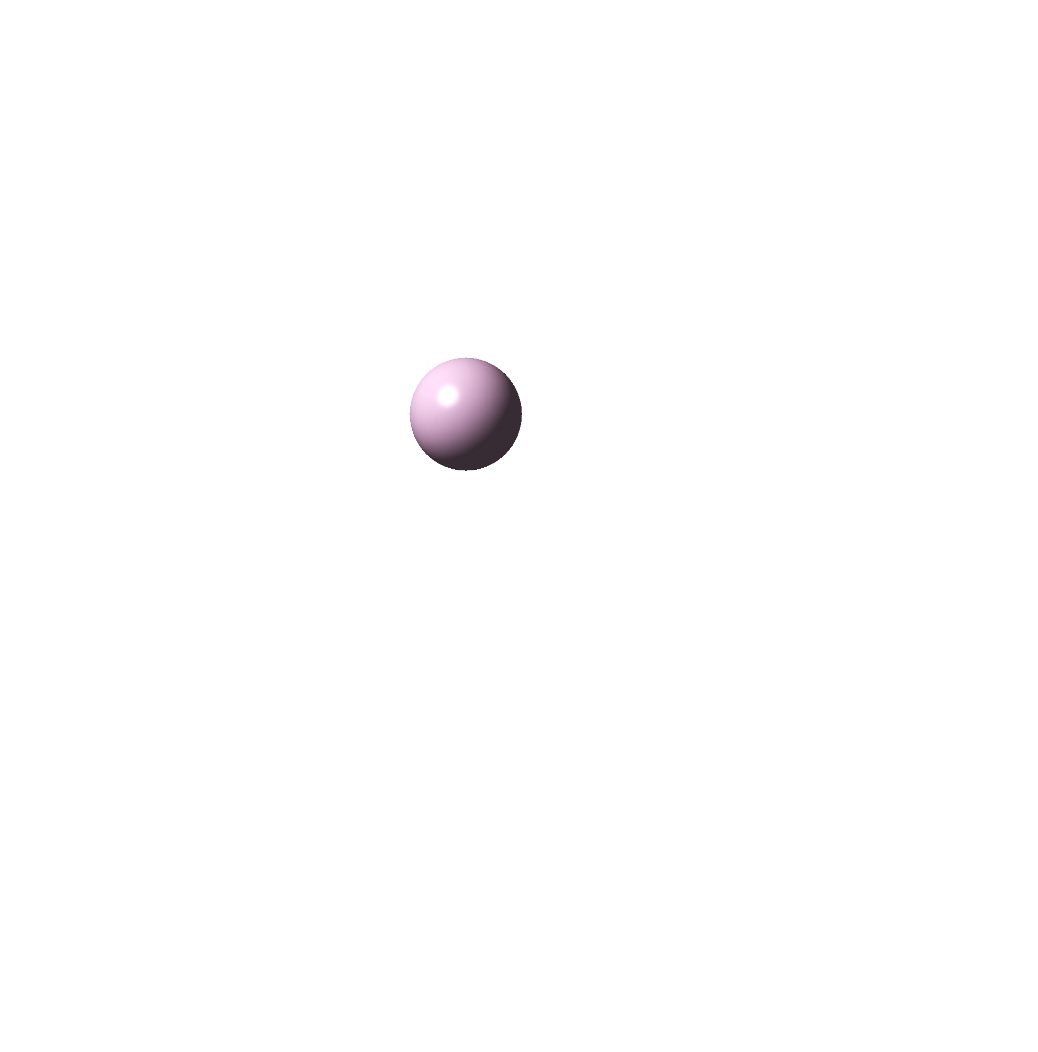** | **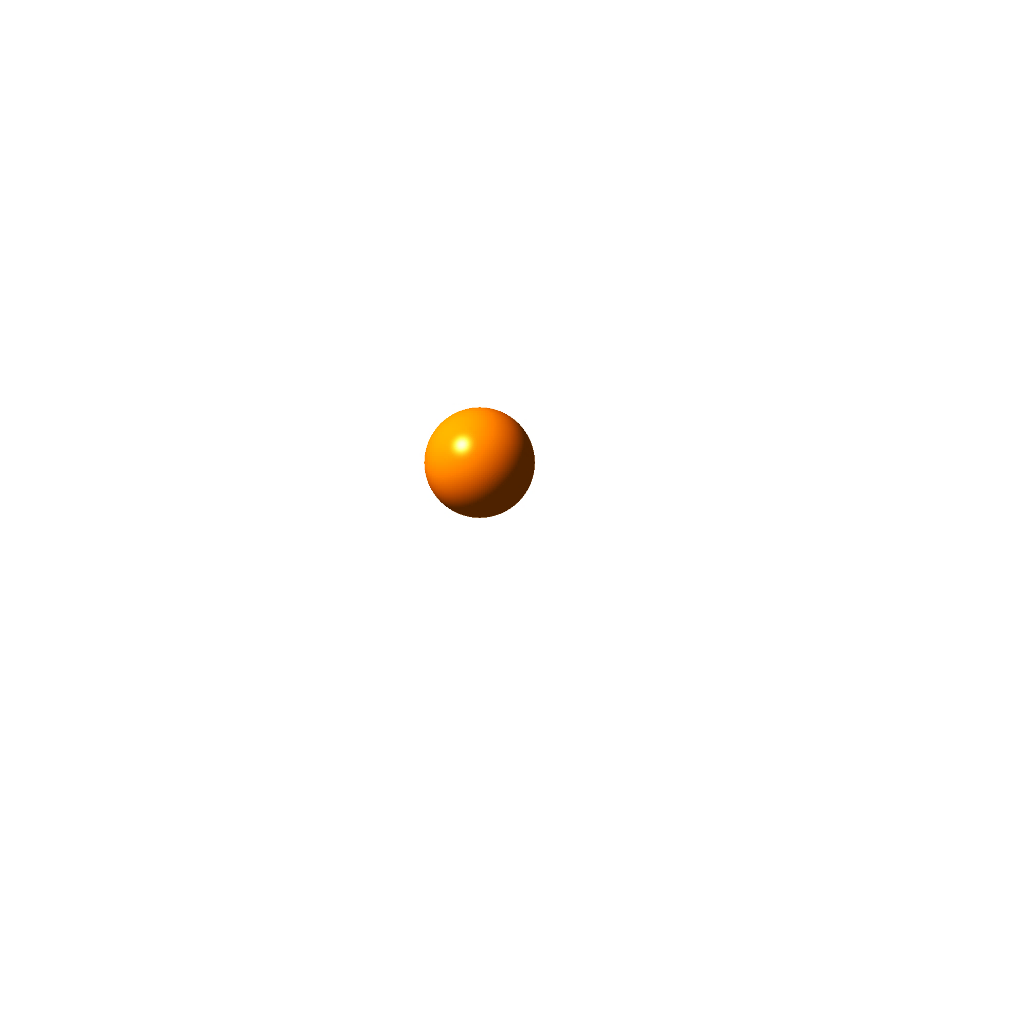** | 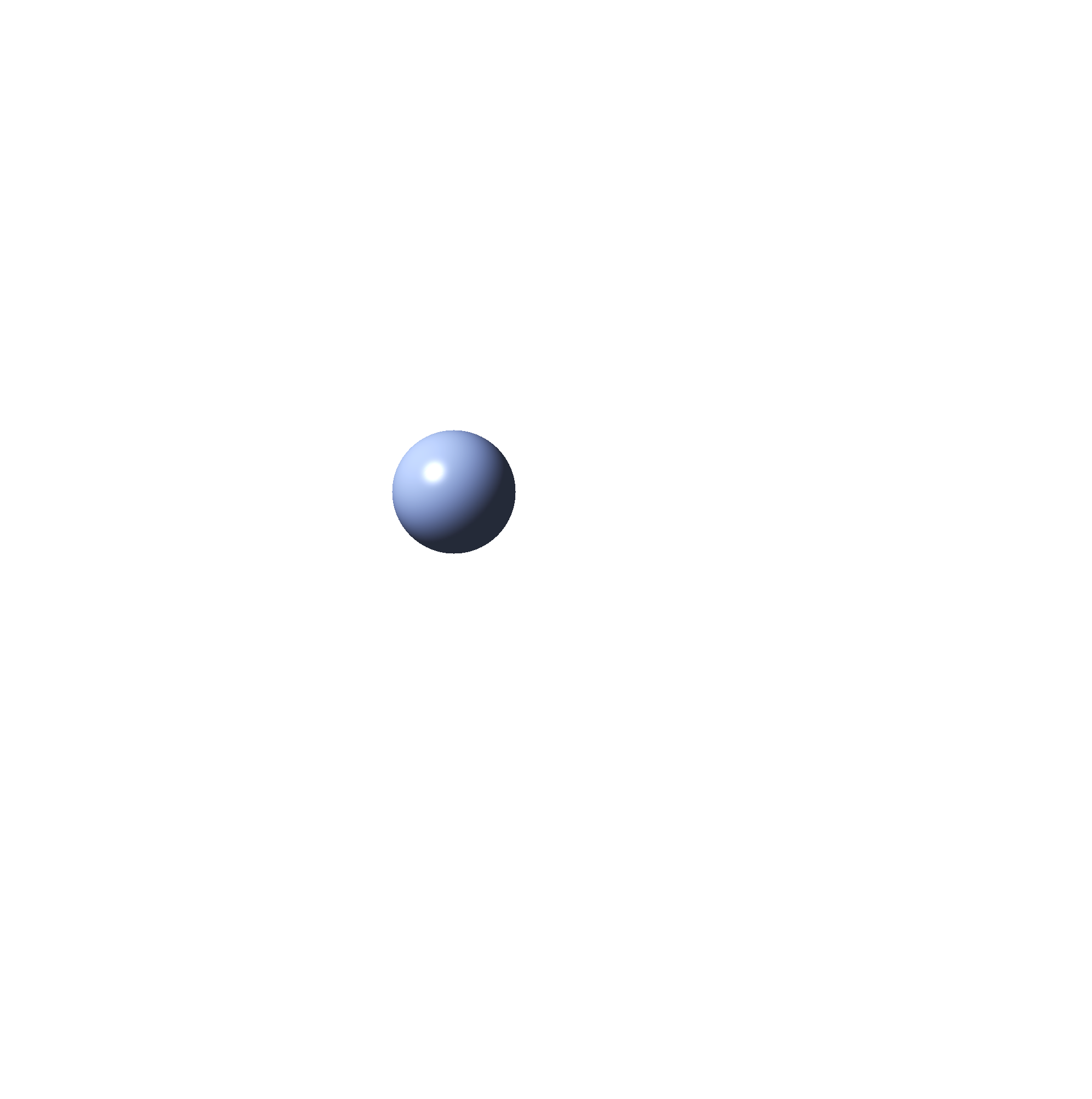 | **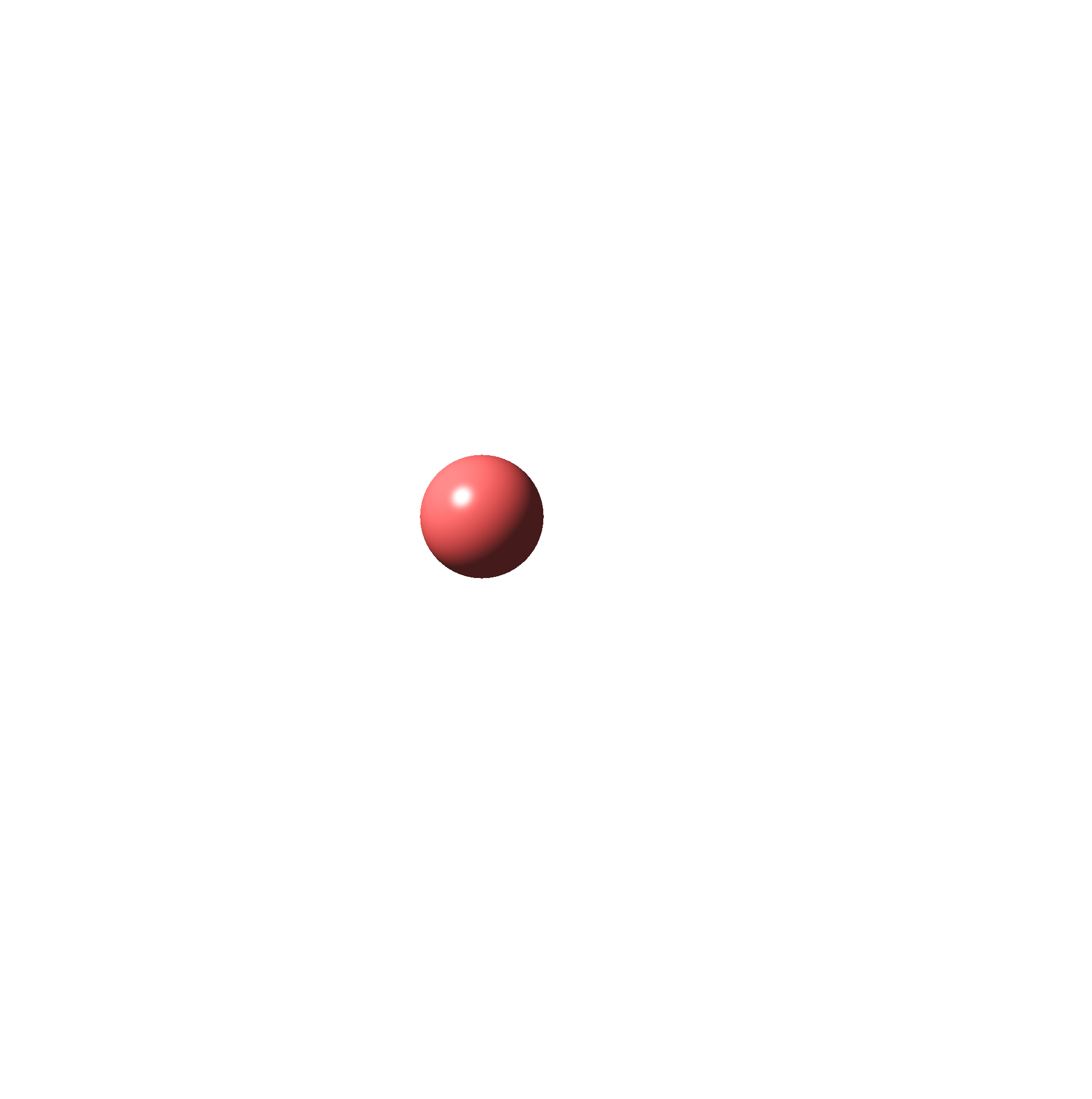** |
| --- | --- | --- | --- | --- | --- | --- | --- | --- |
| **Hydrogen** | **Boron** | **Carbon** | **Nitrogen** | **Oxygen** | **Aluminum** | **Phosphorus** | **Scandium** | **Titanium** |

**Figure S1:** The Optimized Geometry and Molecular Electrostatic Potential (MEP) figures of (a) Ethylene Oxide, (b) BN(B_12_N_12_), (c) Sc-BN(ScB_11_N_12_), (d) Ti-BN(TiB_11_N_12_), (e) AlN(Al_12_N_12_), (f) Sc-AlN(ScAl_11_N_12_), (g) Ti-AlN(TiAl_11_N_12_), (h) AlP(Al_12_P_12_), (i) Sc-AlP(ScAl_11_P_12_), and (j) Ti-AlP(TiAl_11_P_12_) adsorbent nanocages. The color scheme (Red to blue) for the MEP surface ranges from -0.01 a.u. to +0.01 a.u., indicating the electron-rich and electron-deficient regions of the surface, respectively in B3LYP/6-31G(d,p) method. The MEP surfaces are generated with 0.0004 electron/bohr^3^ iso-value.


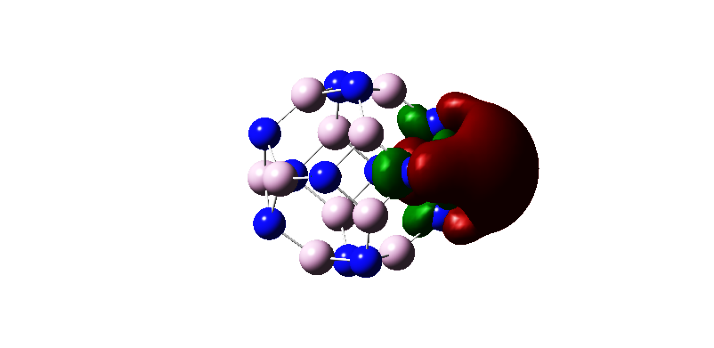


| **HOMO** | **LUMO** | **HOMO** | **LUMO** | **HOMO** | **LUMO** |
| --- | --- | --- | --- | --- | --- |
| 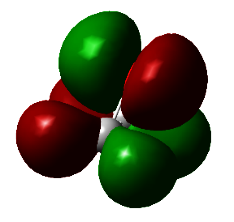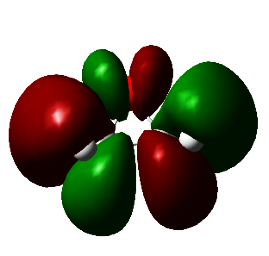 |  | 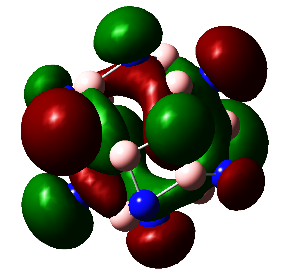 | 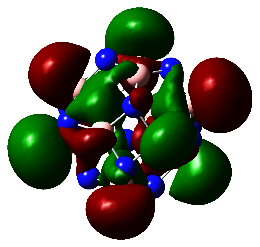 | 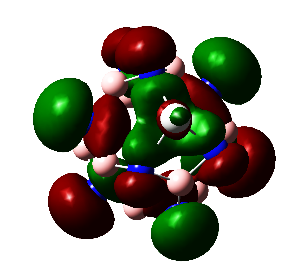 | 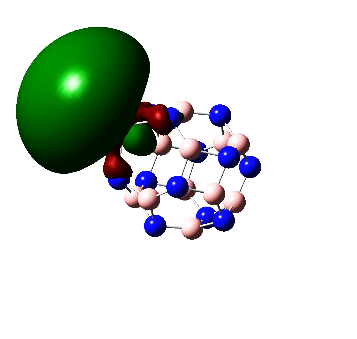 |
| 1. **Ethylene Oxide** | | 1. **BN Nanocage** | | 1. **Sc-BN Nanocage** | |
| 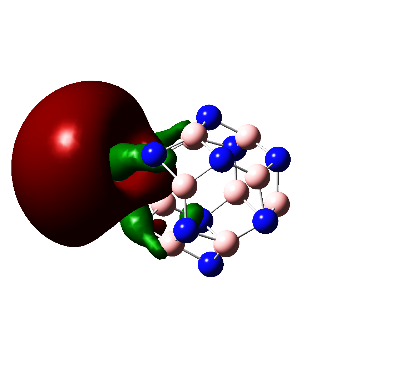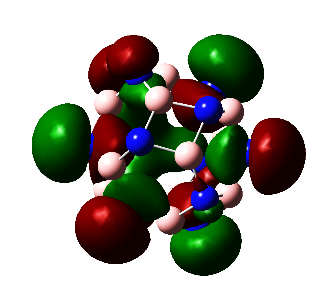 |  | 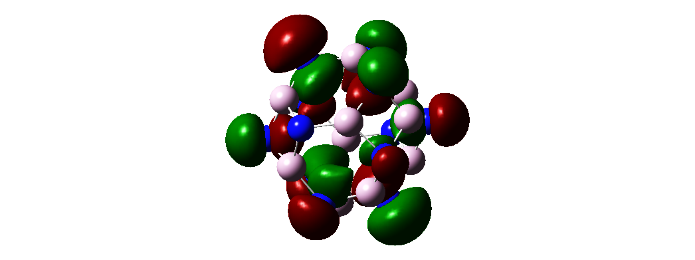 | 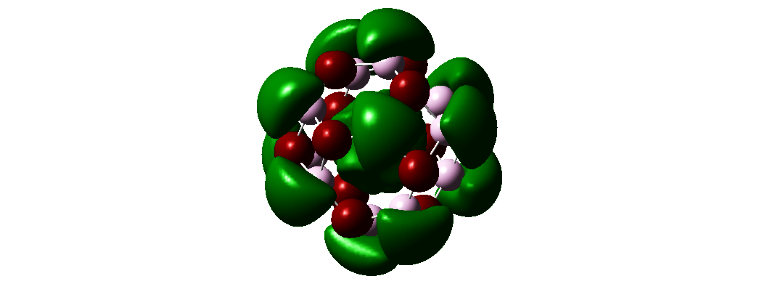 | 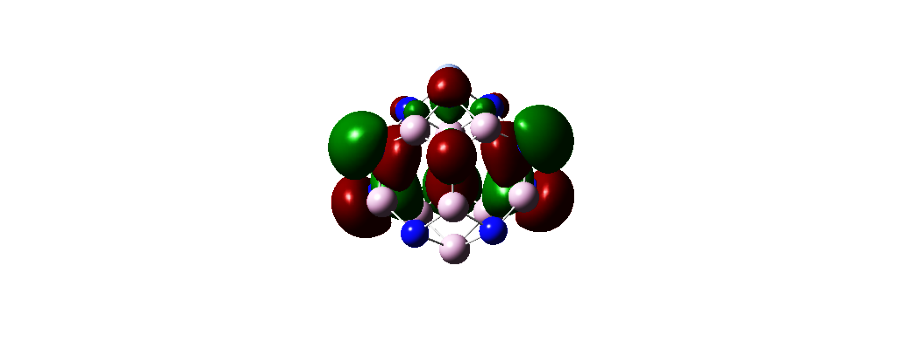 | 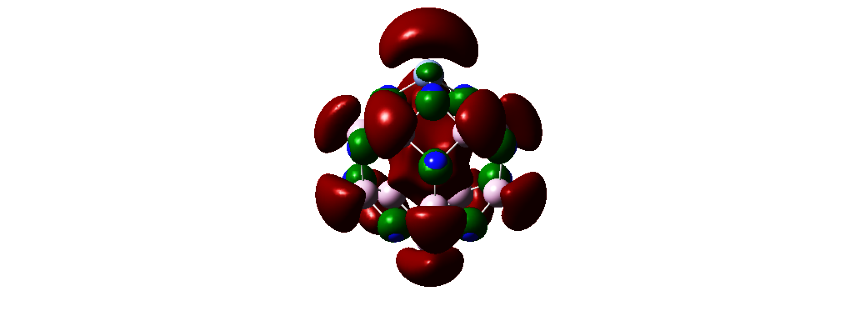 |
| 1. **Ti-BN Nanocage** | | 1. **AlN Nanocage** | | 1. **Sc-AlN Nanocage** | |
| 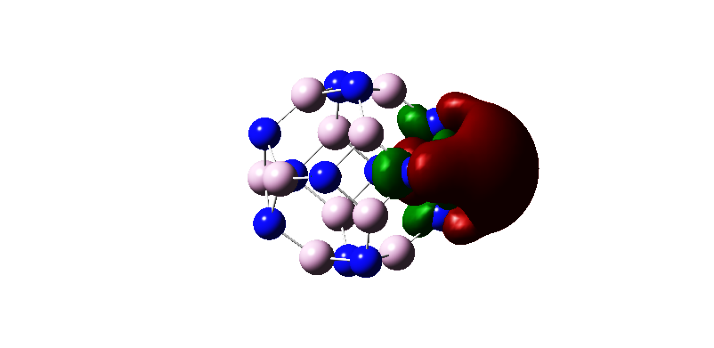 | 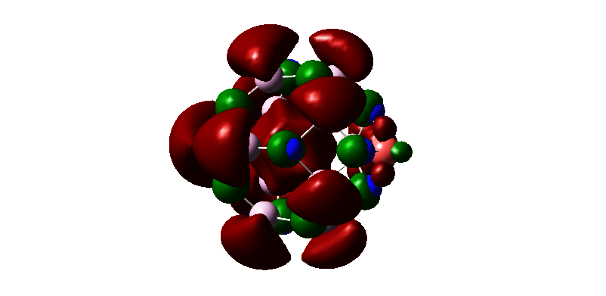 | 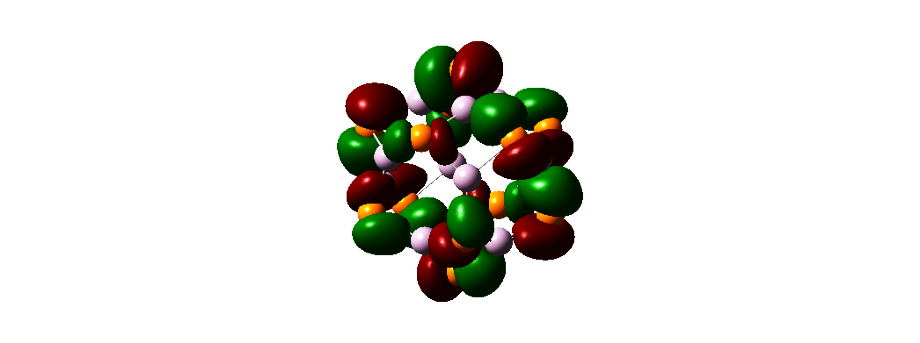 | 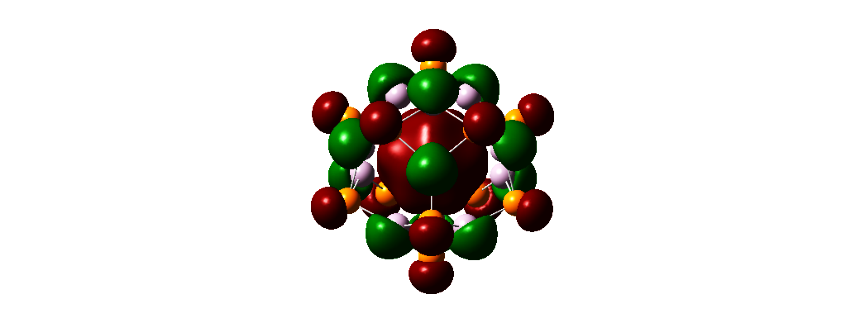 | 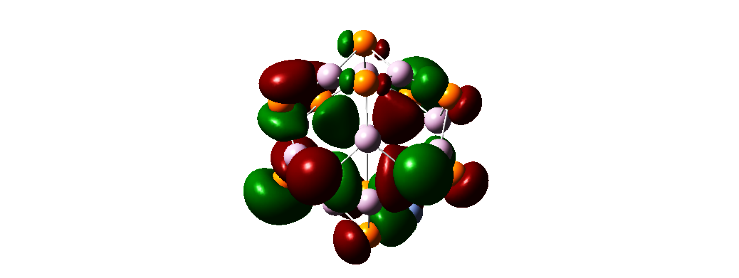 | 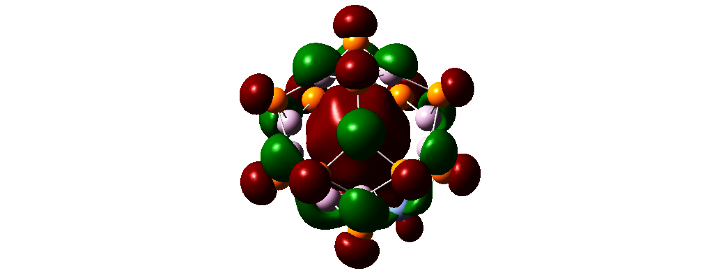 |
| 1. **Ti-AlN Nanocage** | | 1. **AlP Nanocage** | | 1. **Sc-AlP Nanocage** | |
| 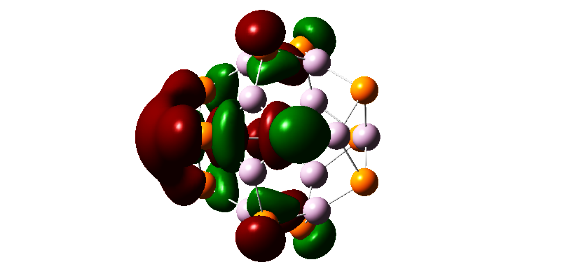 | 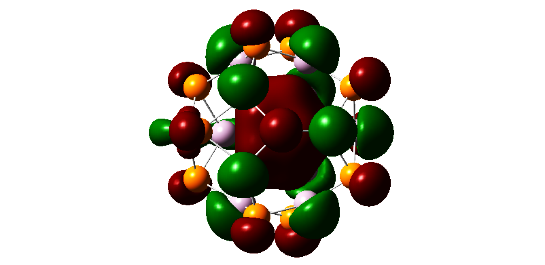 |  |  |  |  |
| 1. **Ti-AlP Nanocage** | |  | |  | |

**Figure S2:** The Frontier molecular orbitals (FMO) levels of (a) Ethylene Oxide, (b) BN(B_12_N_12_), (c) Sc-BN(ScB_11_N_12_), (d) Ti-BN(TiB_11_N_12_), (e) AlN(Al_12_N_12_), (f) Sc-AlN(ScAl_11_N_12_), (g) Ti-AlN(TiAl_11_N_12_), (h) AlP(Al_12_P_12_), (i) Sc-AlP(ScAl_11_P_12_), and (j) Ti-AlP(TiAl_11_P_12_) adsorbent nanocages in ωB97X-D/6-31G(d,p) method. The HOMO and LUMO figures are generated with the iso-value of 0.02 electron/bohr^3^.

| **HOMO** | **LUMO** | **HOMO** | **LUMO** | **HOMO** | **LUMO** |
| --- | --- | --- | --- | --- | --- |
| **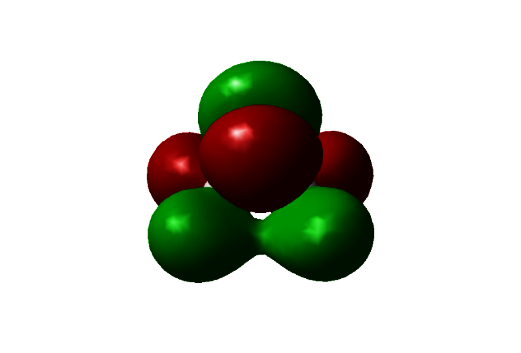** | **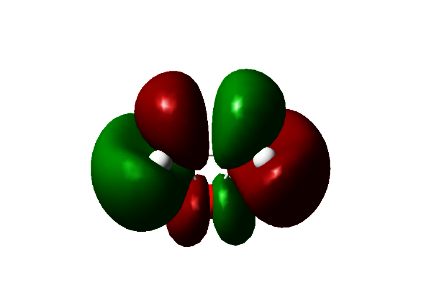** | **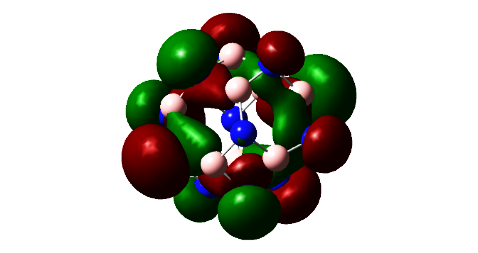** | 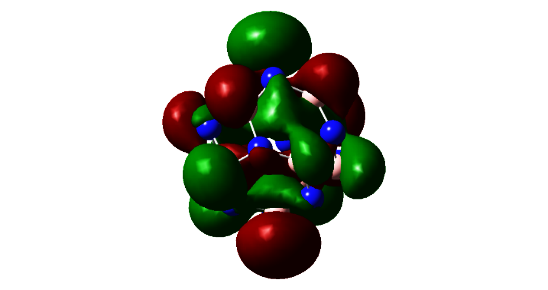 | 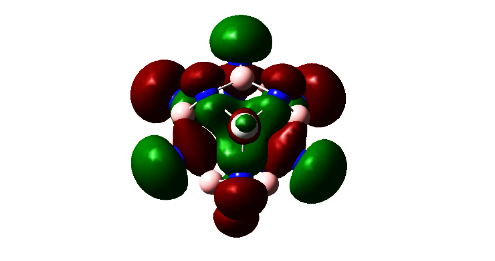 | 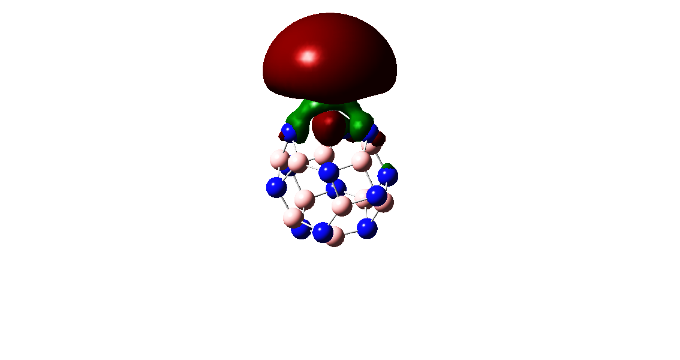 |
| 1. **Ethylene Oxide** | | 1. **BN Nanocage** | | 1. **Sc-BN Nanocage** | |
| 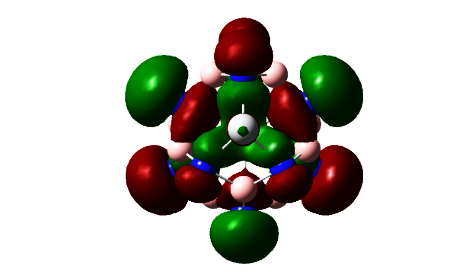 | 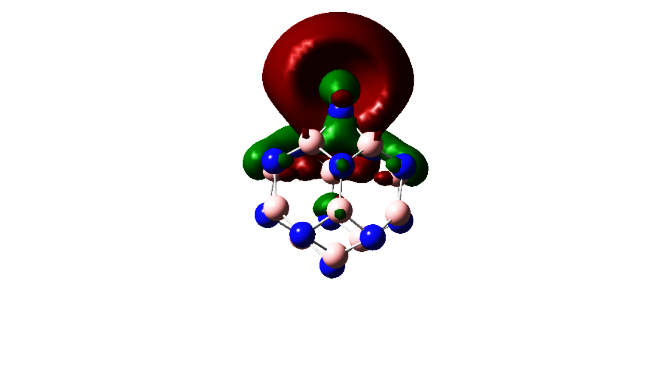 | 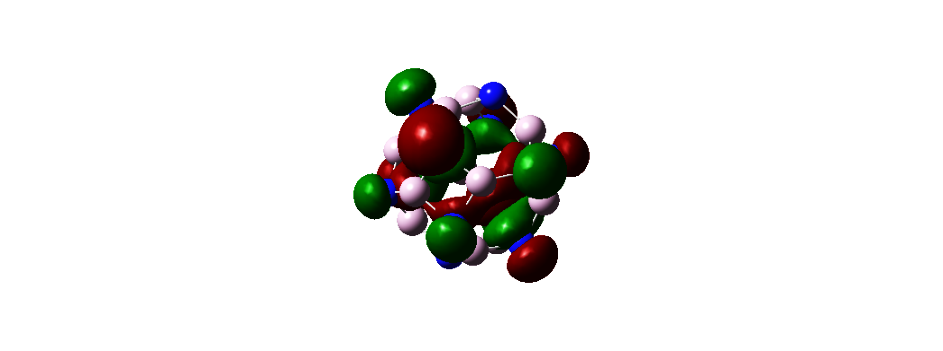 | 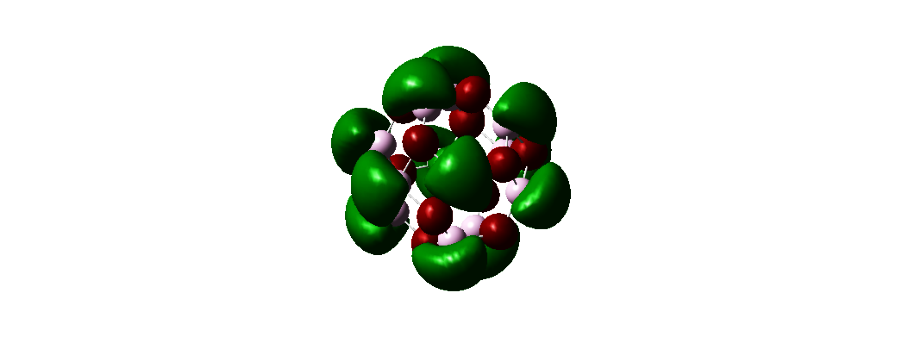 | 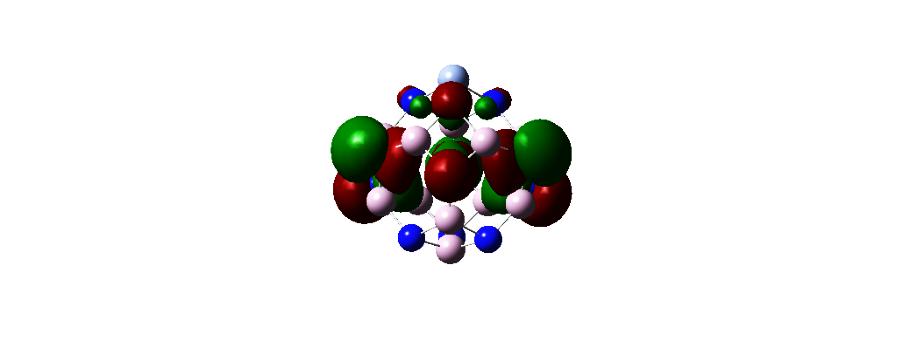 | 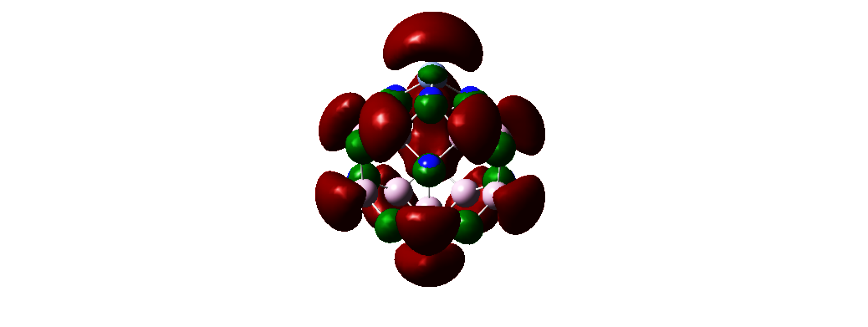 |
| 1. **Ti-BN Nanocage** | | 1. **AlN Nanocage** | | 1. **Sc-AlN Nanocage** | |
| 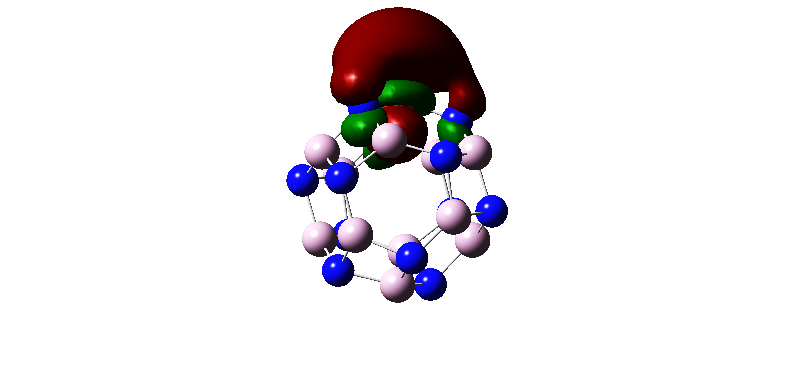 | 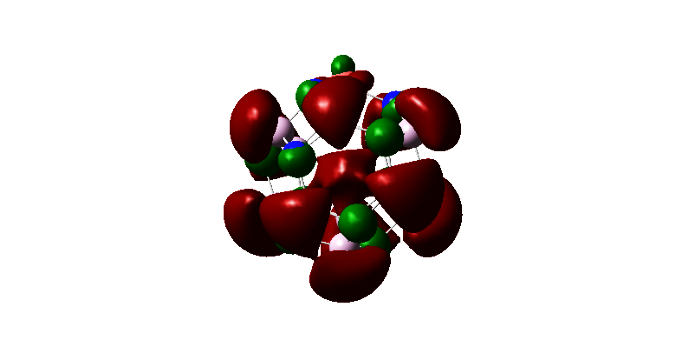 | 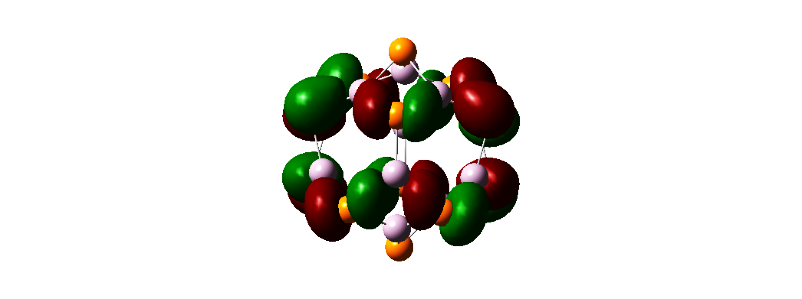 | 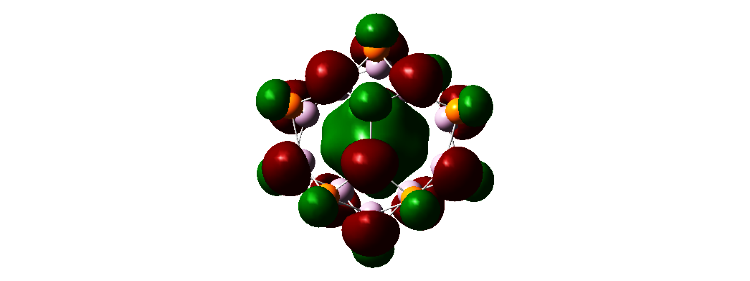 | 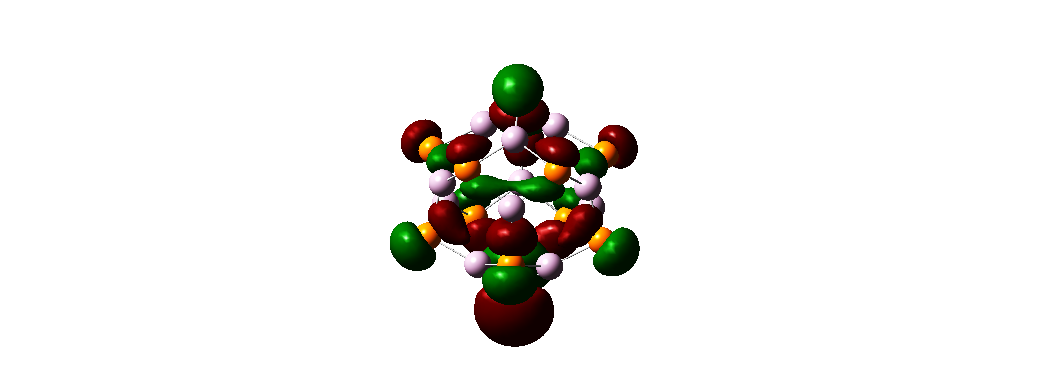 | 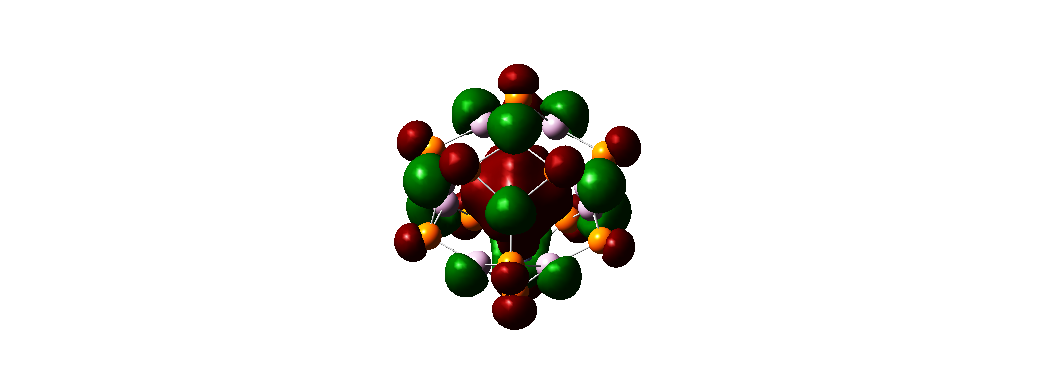 |
| 1. **Ti-AlN Nanocage** | | 1. **AlP Nanocage** | | 1. **Sc-AlP Nanocage** | |
| 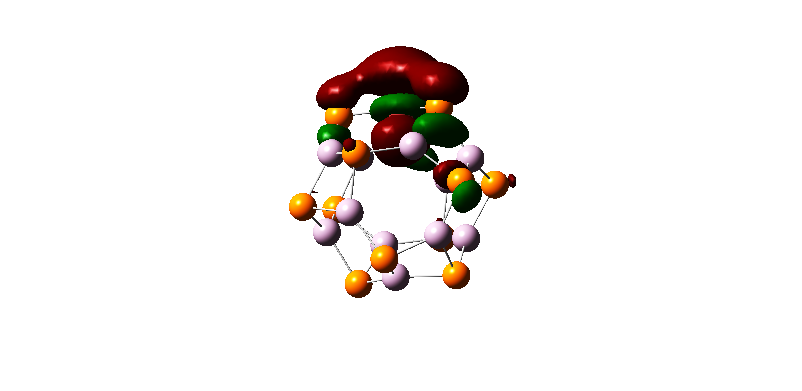 | 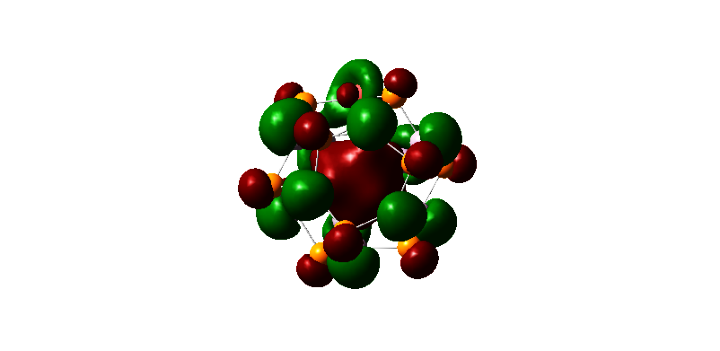 |  |  |  |  |
| 1. **Ti-AlP Nanocage** | |  | |  | |

**Figure S3:** The Frontier molecular orbitals (FMO) level of (a) Ethylene Oxide, (b) BN(B_12_N_12_), (c) Sc-BN(ScB_11_N_12_), (d) Ti-BN(TiB_11_N_12_), (e) AlN(Al_12_N_12_), (f) Sc-AlN(ScAl_11_N_12_), (g) Ti-AlN(TiAl_11_N_12_), (h) AlP(Al_12_P_12_), (i) Sc-AlP(ScAl_11_P_12_), and (j) Ti-AlP(TiAl_11_P_12_) adsorbent nanocages in B3LYP/6-31G(d,p) method. The HOMO and LUMO figures are generated with the iso-value of 0.02 electron/bohr^3^.

**Table S1:** Angles of Bonding and Dihedral Angles in B3LYP/6-31G(d,p) method, where R stands for B, Al atom; Q for N, P atom, and D for the doped atom (The four-membered ring and the six-membered ring, respectively, are represented by the numbers 4M and 6M).

| Angles | BN | Sc-BN | Ti-BN | AlN | Sc-AlN | Ti-AlN | AlP | Sc-AlP | Ti-AlP |
| --- | --- | --- | --- | --- | --- | --- | --- | --- | --- |
| Dihedral Angles(Degree) | | | | | | | | | |
| R-Q-R-Q(4M) | 12.19 | 11.86 | 19.05 | 10.65 | 11.14 | 10.59 | 26.82 | 26.81 | 26.93 |
| R-Q-R-Q(6M) | 25.18 | 32.66 | 25.74 | 18.24 | 19.20 | 18.43 | 45.43 | 46.28 | 45.94 |
| D-Q-R-Q(4M) | - | 1.46 | 0.99 | - | 2.72 | 5.36 | - | 17.12 | 23.34 |
| D-Q-R-Q(6M) | - | 13.34 | 10.99 | - | 10.98 | 13.41 | - | 38.78 | 43.23 |
| Bond Angles(Degree) | | | | | | | | | |
| R-Q-R(4M) | 80.50 | 77.91 | 78.33 | 84.52 | 84.25 | 84.44 | 74.73 | 74.75 | 74.61 |
| Q-R-Q(4M) | 98.20 | 98.77 | 98.61 | 94.49 | 94.64 | 95.57 | 98.87 | 99.03 | 98.95 |
| R-Q-R(6M) | 110.45 | 112.47 | 112.64 | 112.59 | 111.88 | 112.21 | 99.31 | 98.89 | 99.34 |
| Q-R-Q(6M) | 125.80 | 126.98 | 126.75 | 125.78 | 125.81 | 125.73 | 130.17 | 130.24 | 130.08 |
| Q-D-Q(4M) | - | 75.66 | 78.90 | - | 86.75 | 90.86 | - | 95.35 | 99.19 |
| Q-D-Q(6M) | - | 100.62 | 106.18 | - | 111.84 | 117.57 | - | 120.04 | 126.86 |

| **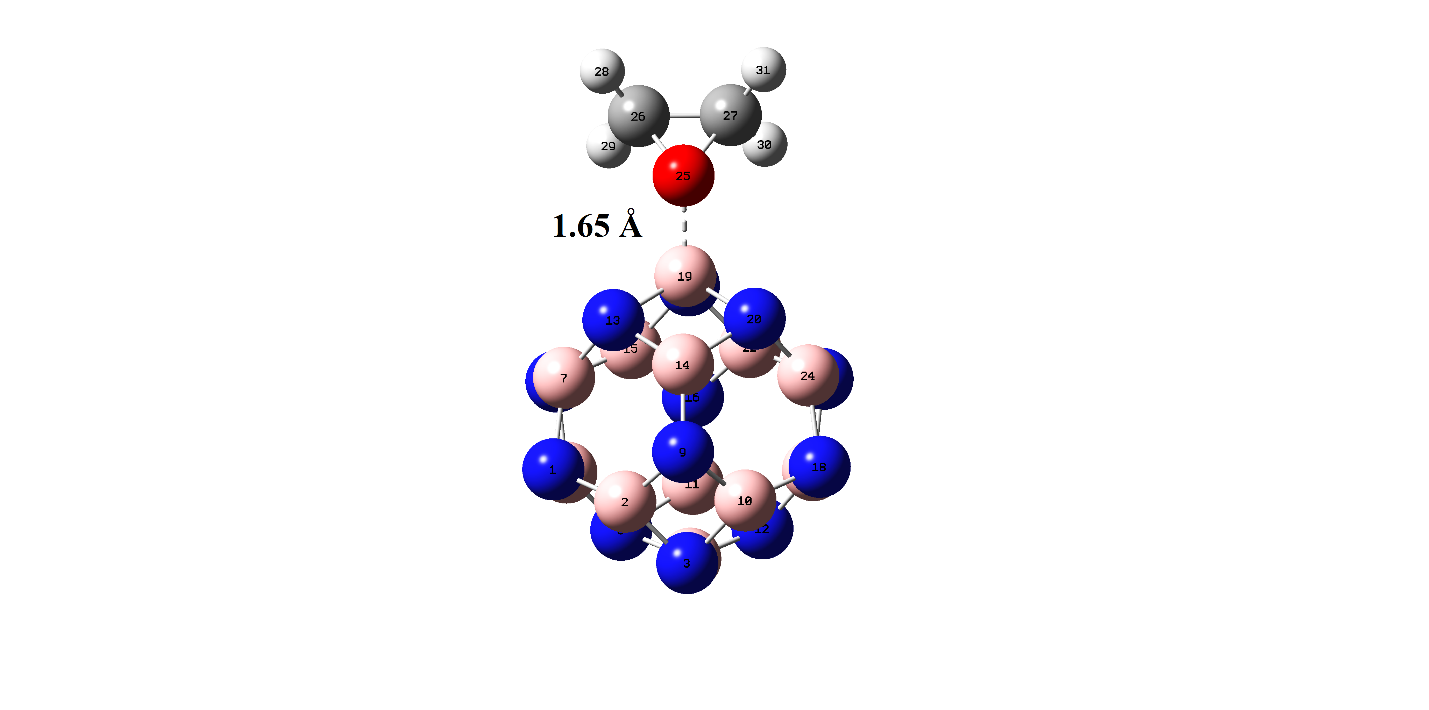** | **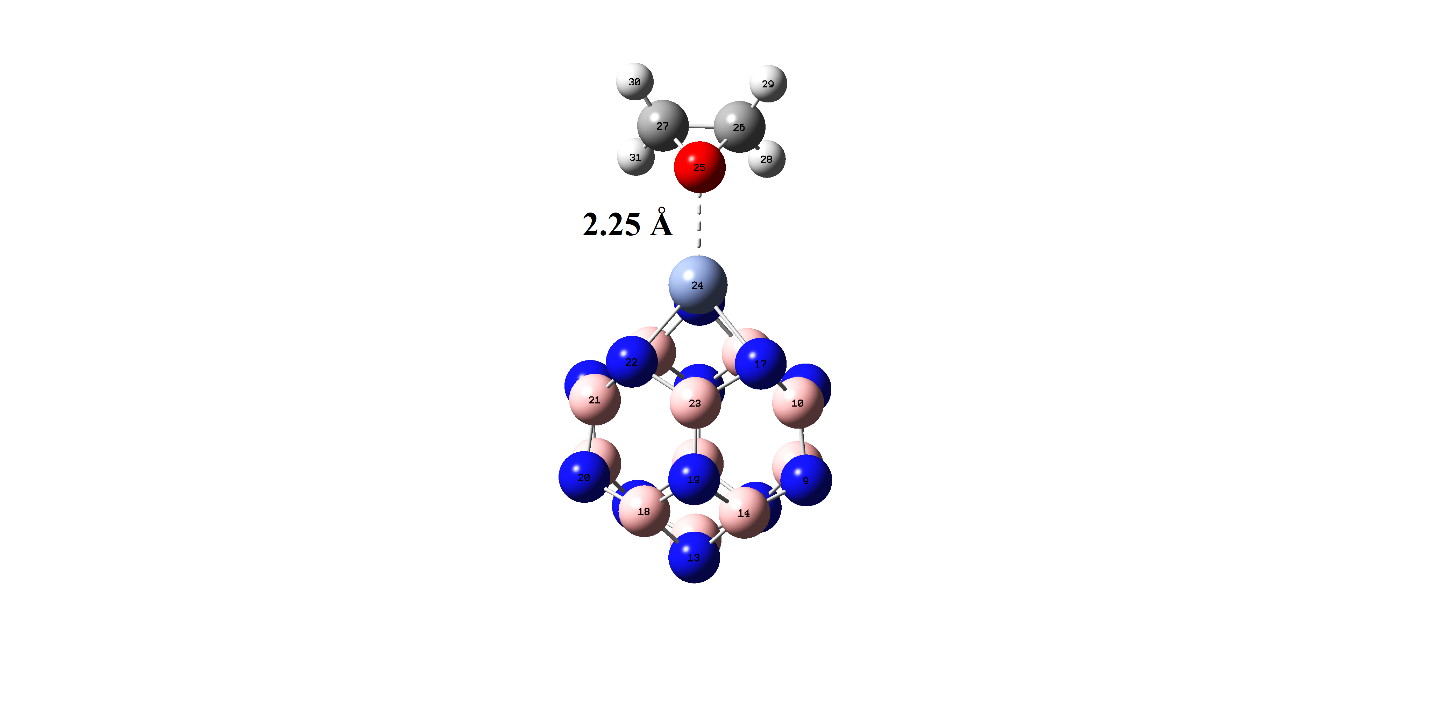** | **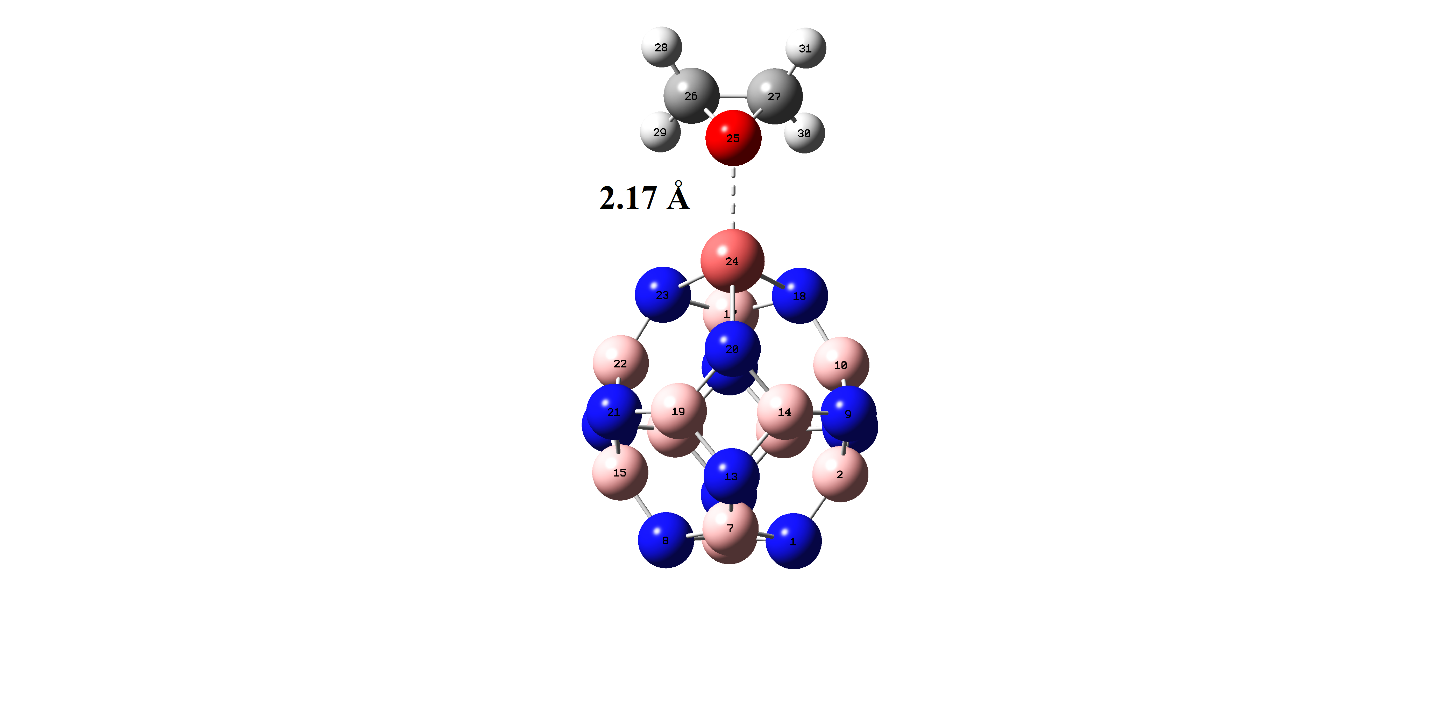** | **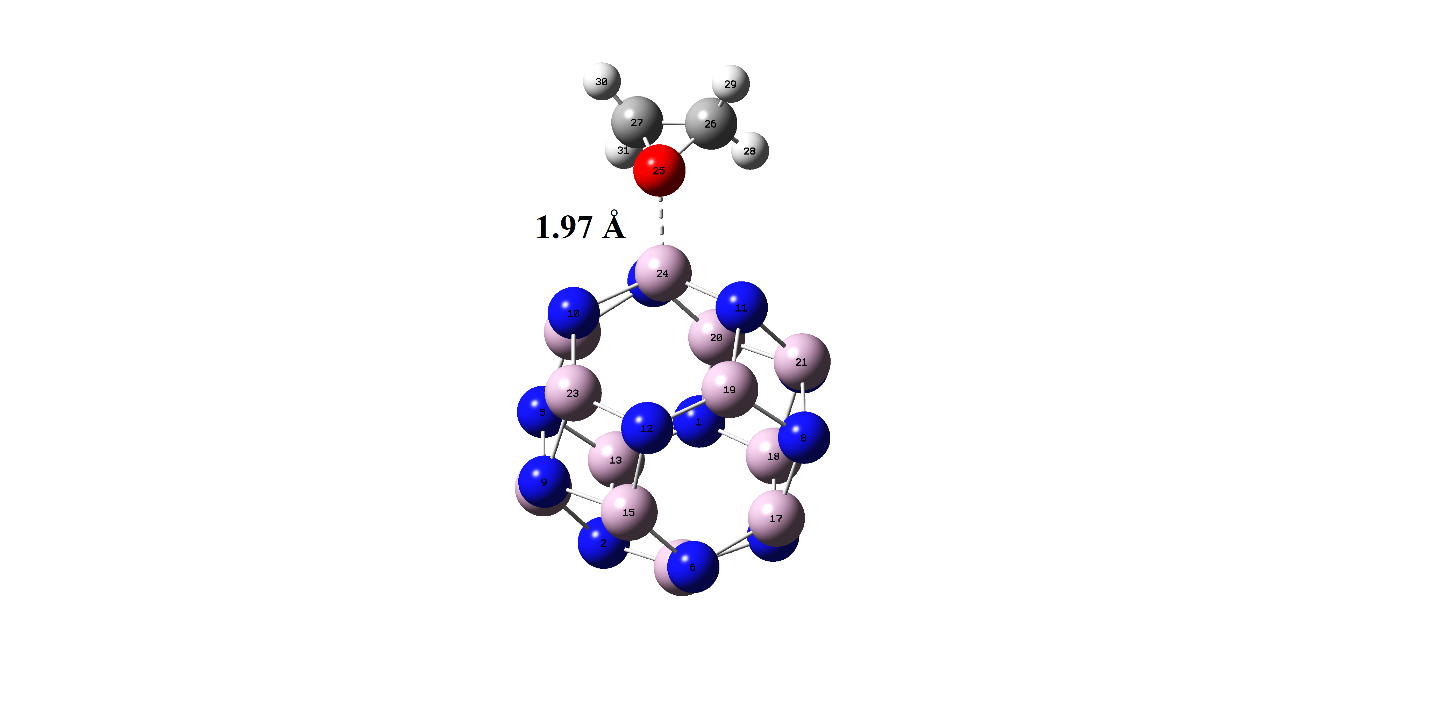** |
| --- | --- | --- | --- |
| 1. **Complex A** | 1. **Complex B** | 1. **Complex C** | 1. **Complex D** |
| **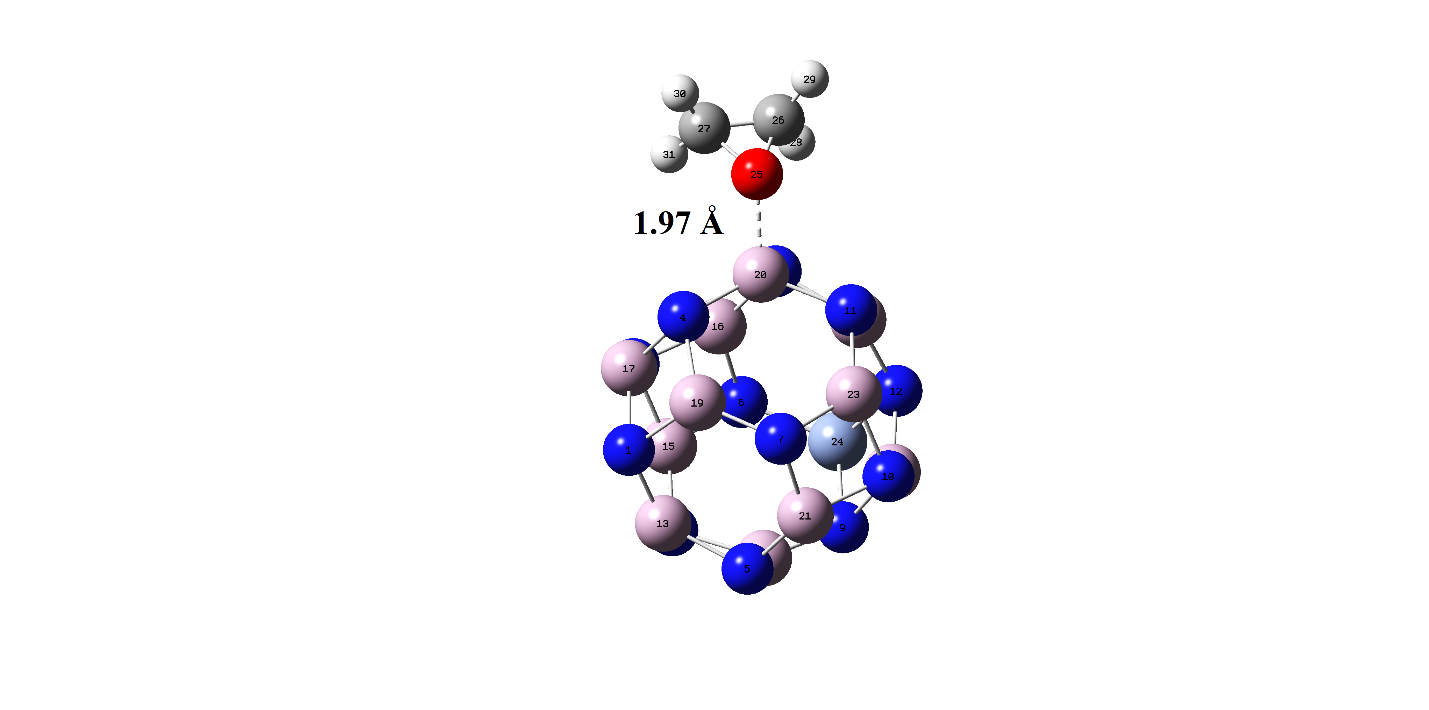** | **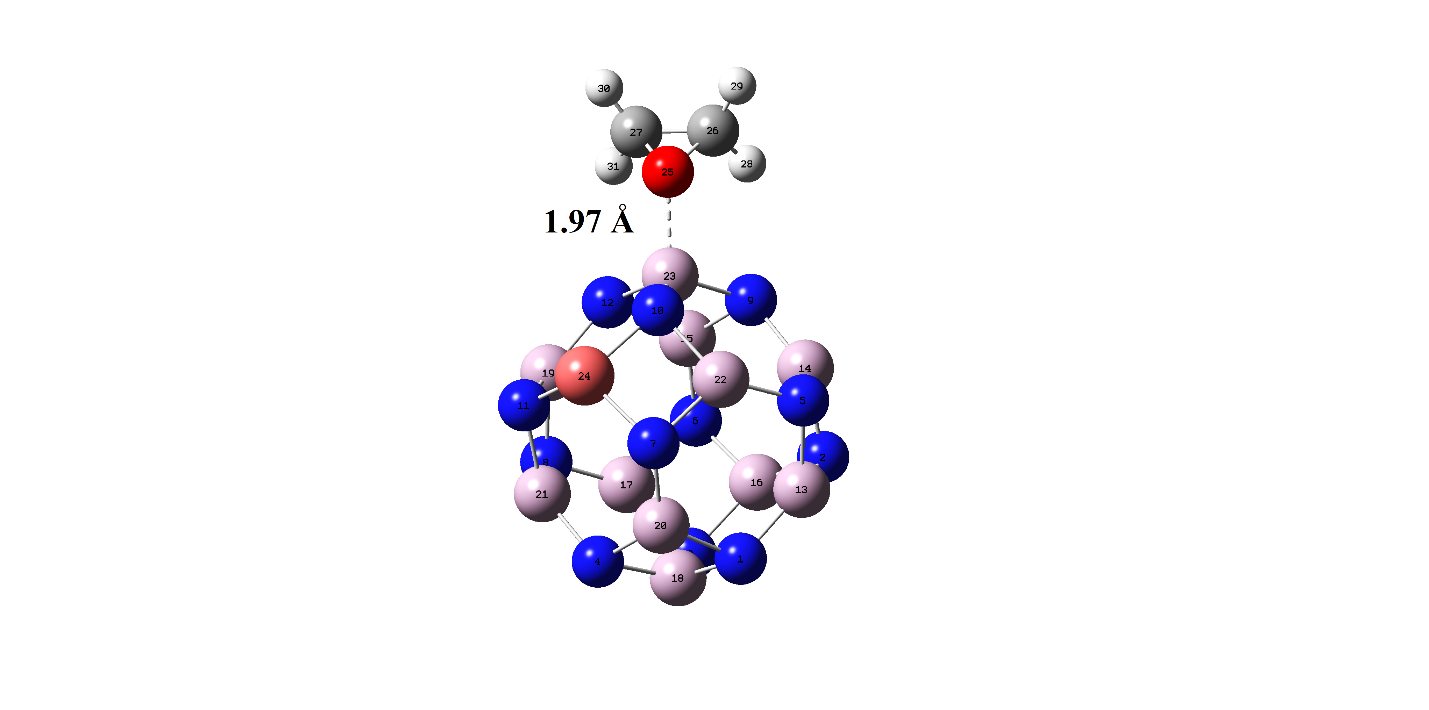** | **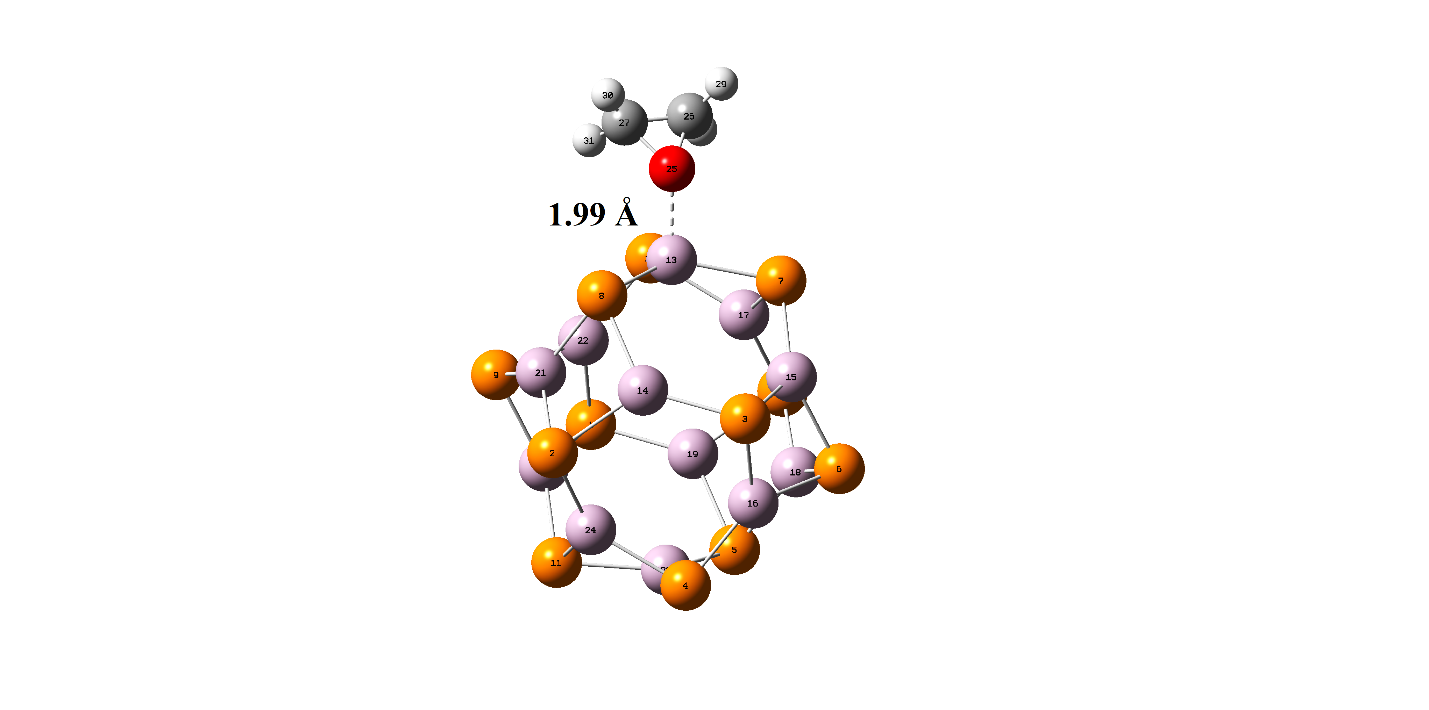** | **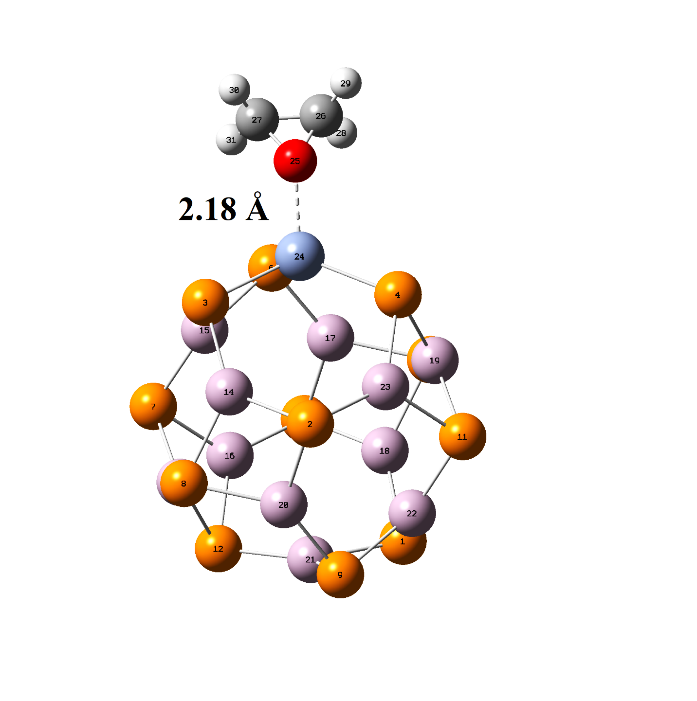** |
| 1. **Complex E** | 1. **Complex F** | 1. **Complex G** | 1. **Complex H** |
| **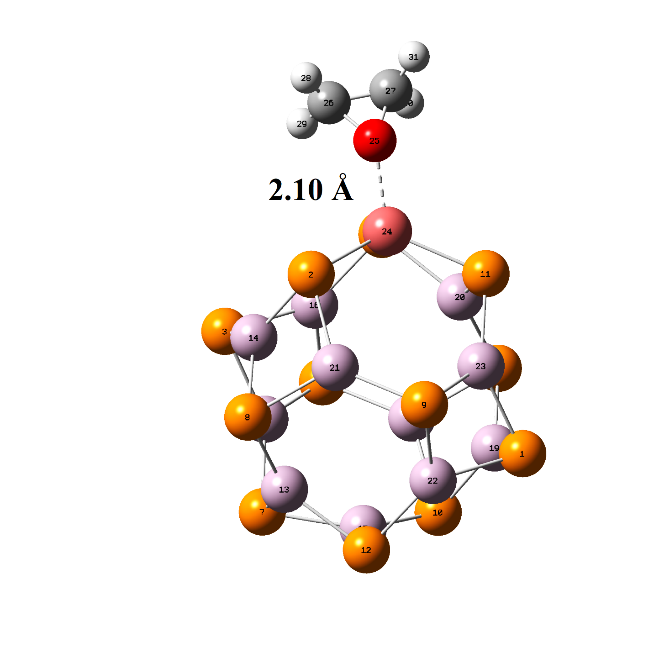** |  |  |  |
| 1. **Complex I** |  |  |  |

**Figure S4:** Optimized Geometry of EO adsorbed complexes i.e., (a) Complex A (b) Complex B, (c) Complex C, (d) Complex D, (e) Complex E, (f) Complex F, (g) Complex G, (h) Complex H, and (i) Complex I. The dashed line denotes the shortest distance between the adsorbate and adsorbents in the B3LYP/6-31G(d,p) method.

**Table S2:** Adsorption Energy, $E_{Ads.}$ in KJ/mole; BSSE corrected adsorption energy, $E_{Ad. CP}$ in KJ/mole; Dipole Moment, $\mu_{D}$ in Debye; Distance between adsorbate and adsorbent, d in angstrom (Å), minimum and maximum frequencies (ν_min_ & ν_max_  ) in cm^−1^, and charge transfer from adsorbate to the adsorbent $Q_{\mathrm{NBO}}$(e unit) in e unit in B3LYP/6-31G(d,p) method.

| Systems | d | $\boldsymbol{\mu}_{\mathbf{D}}$ | $\mathbf{E}_{\mathbf{Ads.}}$ | $\mathbf{E}_{\mathbf{Ad.CP}}$ | $\mathbf{Q}_{\mathbf{NBO}}$ | $\boldsymbol{\nu}_{\boldsymbol{min}}$ | $\boldsymbol{\nu}_{\boldsymbol{max}}$ |
| --- | --- | --- | --- | --- | --- | --- | --- |
| $\mathbf{C}_{\mathbf{2}}\mathbf{H}_{\mathbf{4}}\mathbf{O}$ | - | 1.95 | - | - | - | 818.46 | 3188.66 |
| Pristine BN | - | 0 | - | - | - | 325.51 | 1447.64 |
| Complex A | 1.65 | 7.51 | -70.21 | -51.96 | 0.265 | 30.39 | 3275.30 |
| Sc-BN | - | 7.19 | - | - | - | 199.49 | 1443.80 |
| Complex B | 2.25 | 11.49 | -126.44 | -108.49 | 0.118 | 8.12 | 3255.41 |
| Ti-BN | - | 3.19 | - | - | - | 189.94 | 1445.33 |
| Complex C | 2.17 | 10.59 | -113.49 | -92.76 | 0.133 | 18.12 | 3256.32 |
| Pristine AlN | - | 0 | - | - | - | 158.52 | 945.72 |
| Complex D | 1.97 | 6.42 | -117.11 | -96.44 | 0.120 | 30.59 | 3261.55 |
| Sc-AlN | - | 3.57 | - | - | - | 146.15 | 944.70 |
| Complex E | 1.97 | 6.83 | -113.76 | -92.78 | 0.119 | 38.41 | 3260.57 |
| Ti-AlN | - | 0.797 | - | - | - | 143.02 | 946.59 |
| Complex F | 1.97 | 6.44 | -119.05 | -97.72 | 0.126 | 32.85 | 3257.42 |
| Pristine AlP | - | 0 | - | - | - | 93.88 | 542.95 |
| Complex G | 1.99 | 7.80 | -95.75 | -77.43 | 0.122 | 31.70 | 3262.14 |
| Sc-AlP | - | 3.34 | - | - | - | 80.51 | 543.54 |
| Complex H | 2.18 | 9.77 | -136.43 | -118.79 | 0.145 | 24.31 | 3259.18 |
| Ti-AlP | - | 0.35 | - | - | - | 73.86 | 542.16 |
| Complex I | 2.10 | 8.88 | -100.35 | -79.99 | 0.159 | 18.90 | 3259.73 |

**Table S3:** Thermodynamic parameters; Sum of electronic and thermal Enthalpies, H in Hatree(atomic unit); Sum of electronic and thermal Free Energies, G in Hatree(atomic unit); Enthalpy Change, ΔH in kJ/mole; Gibbs Free Energy Change, ΔG in kJ/mole and Entropy Change, ΔS in kJ/mole.kelvin unit in B3LYP/6-31G(d,p) method.

| Systems | H | G | ΔH | ΔG | ΔS |
| --- | --- | --- | --- | --- | --- |
| $\mathbf{C}_{\mathbf{2}}\mathbf{H}_{\mathbf{4}}\mathbf{O}$ | -153.730405 | -153.758587 |  |  |  |
| Pristine BN | -956.005424 | -956.051298 |  |  |  |
| Complex A | -1109.759976 | -1109.814806 | -63.39 | -12.92 | -0.17 |
| Sc-BN | -1691.873613 | -1691.922491 | - |  |  |
| Complex B | -1845.649709 | -1845.712503 | -119.96 | -82.51 | -0.13 |
| Ti-BN | -1780.574776 | -1780.624485 |  |  |  |
| Complex C | -1934.345662 | -1934.407751 | -106.28 | -64.79 | -0.14 |
| Pristine AlN | -3567.143367 | -3567.209335 |  |  |  |
| Complex D | -3720.915734 | -3720.991932 | -110.17 | -63.04 | -0.16 |
| Sc-AlN | -4085.415729 | -4085.482892 |  |  |  |
| Complex E | -4239.186770 | -4239.263740 | -106.69 | -58.45 | -0.16 |
| Ti-AlN | -4174.127889 | -4174.195917 |  |  |  |
| Complex F | -4327.900992 | -4327.978851 | -112.10 | -63.92 | -0.16 |
| Pristine AlP | -7006.236890 | -7006.330708 |  |  |  |
| Complex G | -7160.001116 | -7160.105146 | -88.79 | -41.62 | -0.16 |
| Sc-AlP | -7524.482841 | -7524.578541 |  |  |  |
| Complex H | -7678.262628 | -7678.369823 | -129.65 | -85.84 | -0.15 |
| Ti-AlP | -7613.189335 | -7613.285934 |  |  |  |
| Complex I | -7766.955489 | -7767.063772 | -93.86 | -50.54 | -0.15 |

| **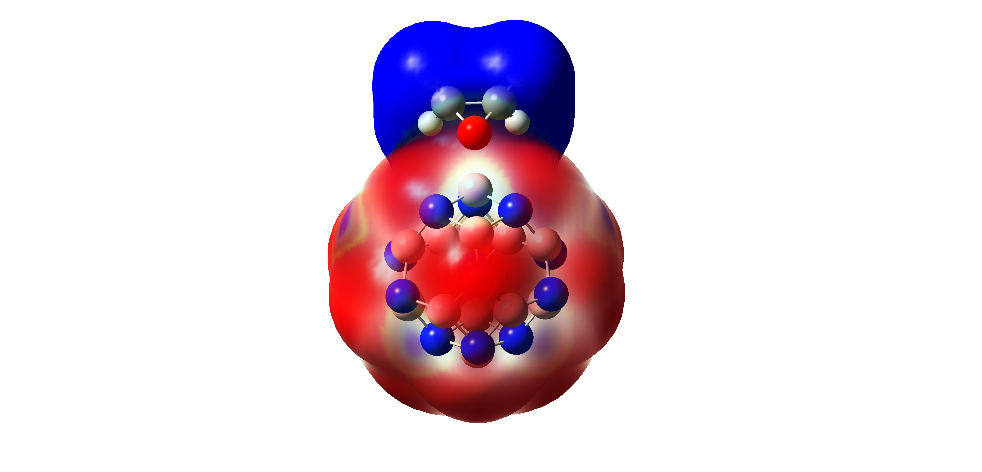** | **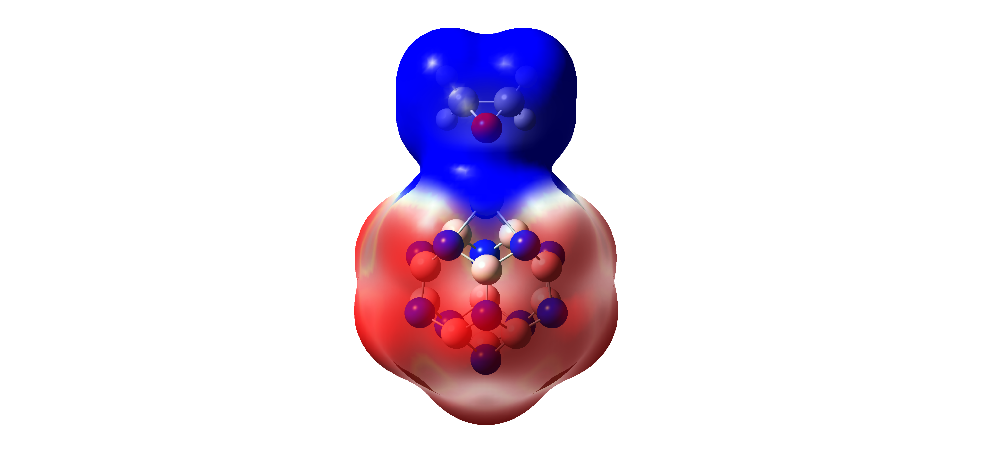** | **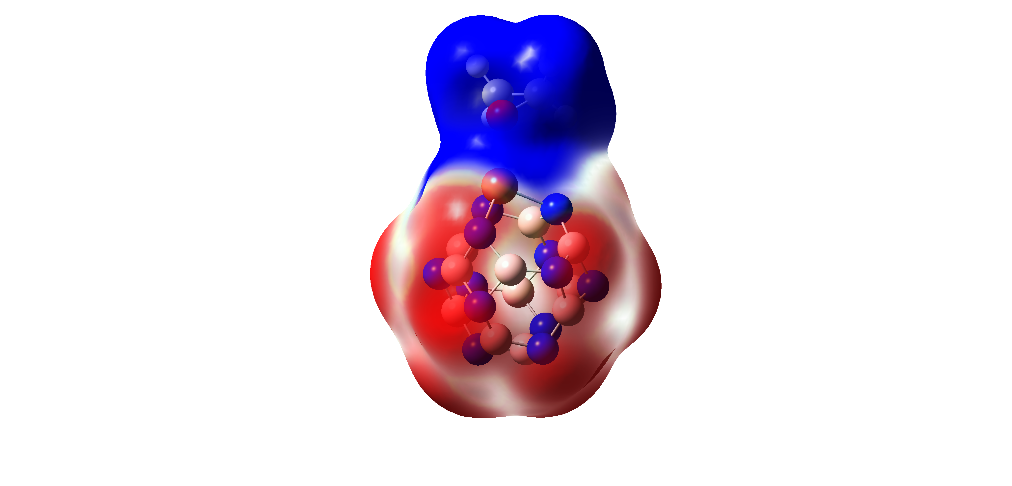** | **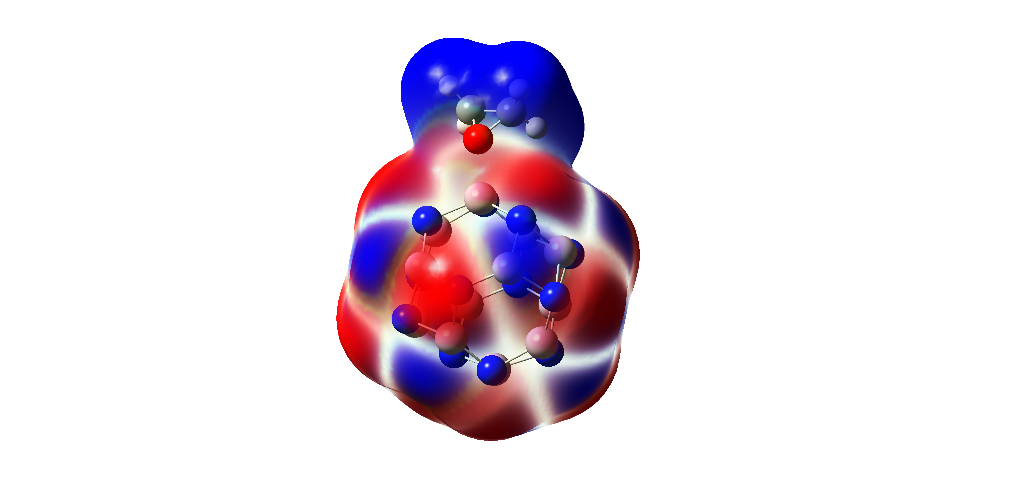** | **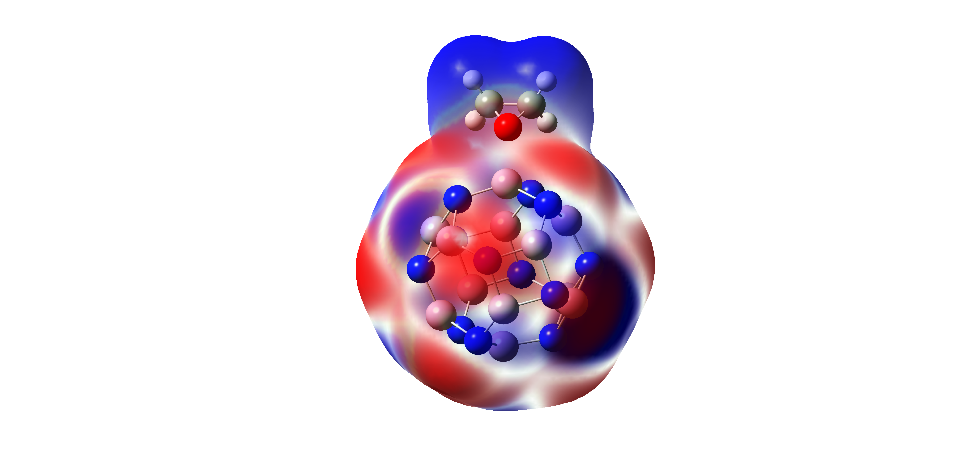** |
| --- | --- | --- | --- | --- |
| 1. **Complex A** | 1. **Complex B** | 1. **Complex C** | 1. **Complex D** | 1. **Complex E** |
| **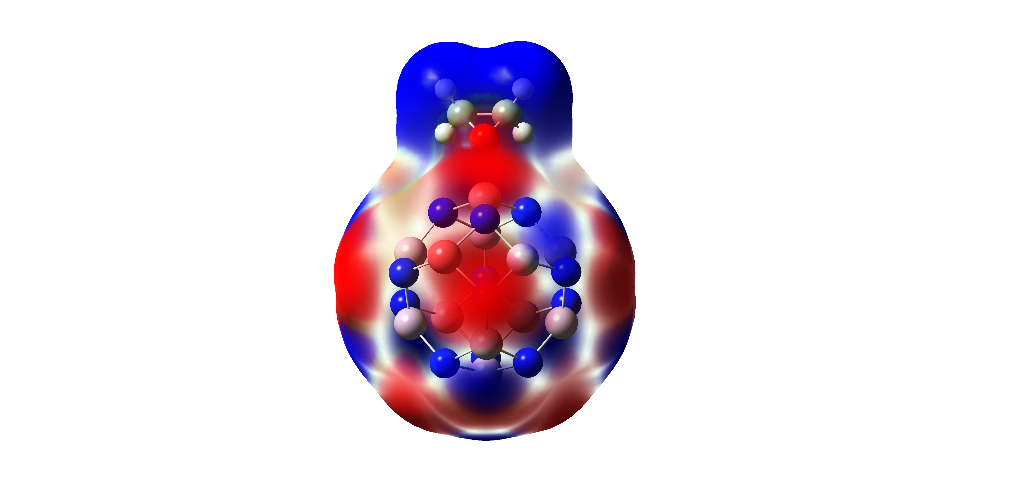** | **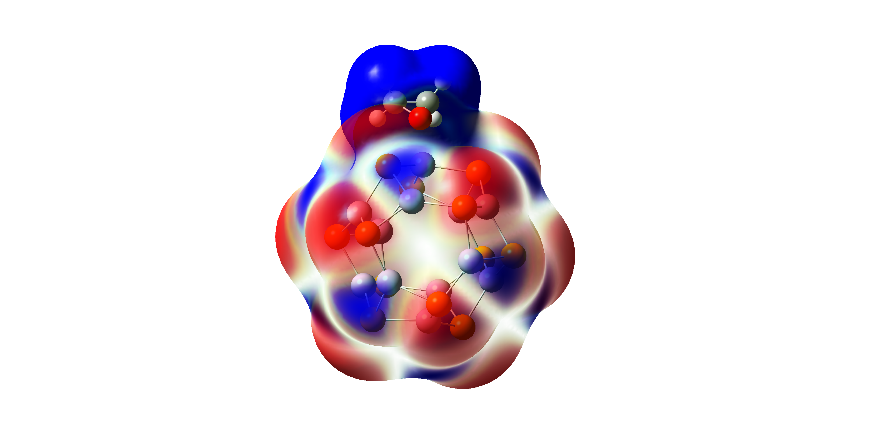** | **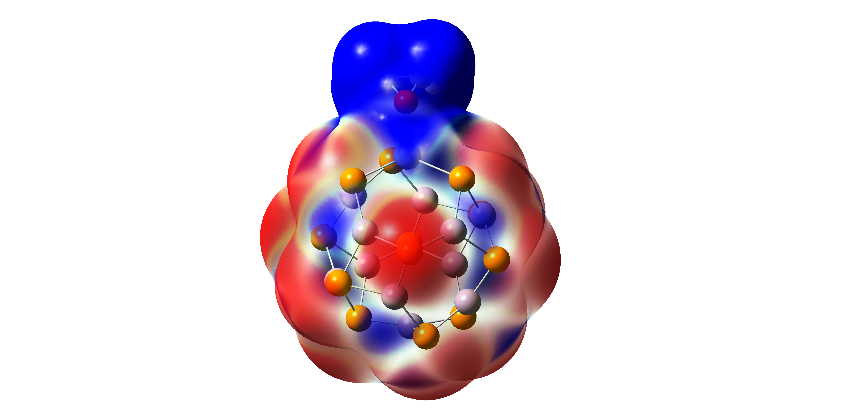** | **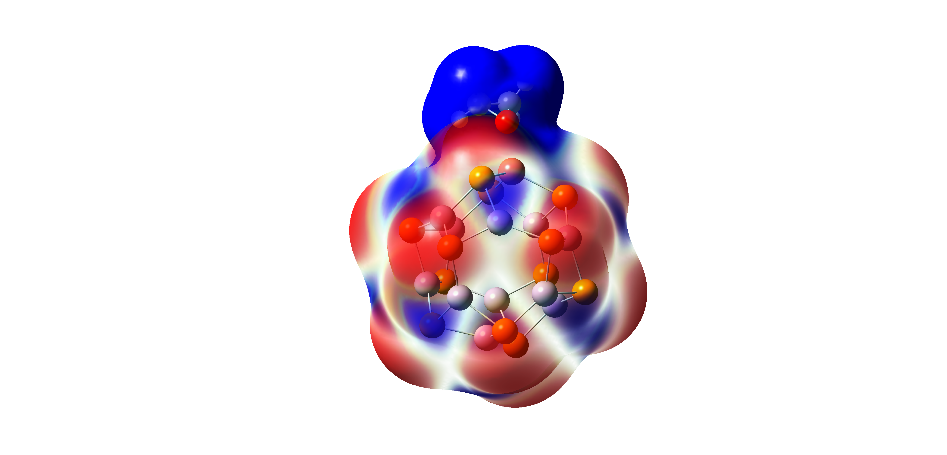** |  |
| 1. **Complex F** | 1. **Complex G** | 1. **Complex H** | 1. **Complex I** |  |

**Figure S5:** The Molecular electrostatic potential (MEP) maps of (a) Complex A, (b) Complex B, (c) Complex C, (d) Complex D, (e) Complex E, (f) Complex F, (g) Complex G, (h) Complex H, and (i) Complex I. The color scheme of the MEP surface (-0.01 a.u. to +0.01 a.u.) shows the electron-rich and electron-deficient areas, respectively in B3LYP/6-31G(d,p) method. The MEP surfaces are generated with 0.0004 electron/bohr^3^ iso-value.

| Systems | $\mathbf{E}_{\mathbf{H}}$ | $\mathbf{E}_{\mathbf{L}}$ | $\mathbf{E}_{\mathbf{g}}$ | %∆$\mathbf{E}_{\mathbf{g}}$ | $\mathbf{E}_{\mathbf{F}}$ | ɸ | %ɸ | μ | η | S | ω |
| --- | --- | --- | --- | --- | --- | --- | --- | --- | --- | --- | --- |
| $\mathbf{C}_{\mathbf{2}}\mathbf{H}_{\mathbf{4}}\mathbf{O}$ | -7.27 | 2.85 | 10.17 | - | -2.21 | 2.21 | - | -2.21 | 5.06 | 0.10 | 0.48 |
| Pristine BN | -7.71 | -0.87 | 6.84 | - | -4.29 | 4.29 | - | -4.29 | 3.42 | 0.15 | 2.69 |
| Complex A | -6.84 | -0.14 | 6.70 | 2.05 | -3.49 | 3.49 | 18.65 | -3.49 | 3.35 | 0.15 | 1.82 |
| Sc-BN | -6.84 | -2.58 | 4.26 | 37.79 | -4.71 | 4.71 | -9.79 | -4.71 | 2.13 | 0.23 | 5.21 |
| Complex B | -6.46 | -1.39 | 5.07 | 25.84 | -3.93 | 3.93 | 8.39 | -3.93 | 2.54 | 0.20 | 3.04 |
| Ti-BN | -5.46 | -2.39 | 3.07 | 55.16 | -3.92 | 3.92 | 8.62 | -3.92 | 1.53 | 0.33 | 5.02 |
| Complex C | -4.75 | -1.58 | 3.17 | 53.66 | -3.16 | 3.16 | 26.34 | -3.16 | 1.59 | 0.32 | 3.16 |
| Pristine AlN | -6.46 | -2.54 | 3.92 | - | -4.50 | 4.50 | - | -4.50 | 1.96 | 0.25 | 5.16 |
| Complex D | -6.01 | -2.13 | 3.88 | 1.09 | -4.07 | 4.07 | 9.55 | -4.07 | 1.94 | 0.26 | 4.26 |
| Sc-AlN | -6.23 | -2.32 | 3.91 | 0.38 | -4.28 | 4.28 | 4.88 | -4.28 | 1.95 | 0.26 | 4.68 |
| Complex E | -5.83 | -1.95 | 3.88 | 1.22 | -3.89 | 3.89 | 13.55 | -3.89 | 1.94 | 0.26 | 3.91 |
| Ti-AlN | -4.38 | -2.47 | 1.91 | 51.26 | -3.43 | 3.43 | 23.78 | -3.43 | 0.96 | 0.52 | 6.14 |
| Complex F | -3.93 | -2.08 | 1.85 | 52.84 | -3.00 | 3.00 | 33.33 | -3.00 | 0.93 | 0.54 | 4.87 |
| Pristine AlP | -6.74 | -3.36 | 3.38 | - | -5.05 | 5.05 | - | -5.05 | 1.69 | 0.296 | 7.55 |
| Complex G | -6.30 | -2.97 | 3.33 | 1.42 | -4.63 | 4.63 | 8.32 | -4.63 | 1.67 | 0.30 | 6.44 |
| Sc-AlP | -6.52 | -3.17 | 3.35 | 0.81 | -4.84 | 4.84 | 4.16 | -4.84 | 1.68 | 0.30 | 6.99 |
| Complex H | -6.19 | -2.87 | 3.32 | 1.80 | -4.53 | 4.53 | 10.30 | -4.53 | 1.66 | 0.30 | 6.19 |
| Ti-AlP | -6.24 | -3.22 | 3.02 | 10.77 | -4.73 | 4.73 | 6.34 | -4.73 | 1.51 | 0.33 | 7.41 |
| Complex I | -5.24 | -2.91 | 2.33 | 31.11 | -4.08 | 4.08 | 19.21 | -4.08 | 1.16 | 0.43 | 7.14 |

**Table S4:** HOMO energy, $E_{H}$ in eV; LUMO energy, $E_{L}$ in eV; Energy Gap, $E_{g}$ in eV; Change in energy gap, %∆$E_{g}$; Fermi Level energy,$E_{F}$ in eV, work function, ɸ in eV and Changes in work function, %ɸ; Chemical Potential, μ; Global Hardness, η; Softness, S; and Global Electrophilicity, ω in B3LYP/6-31G(d,p) method.

| **HOMO** | **LUMO** | **HUMO** | **LUMO** | **HOMO** | **LUMO** |
| --- | --- | --- | --- | --- | --- |
| **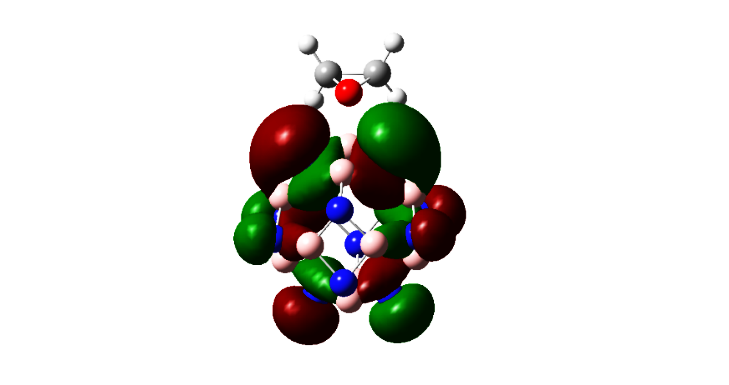** | **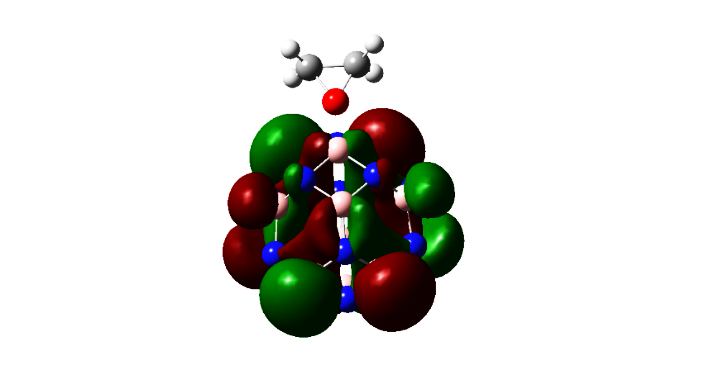** | **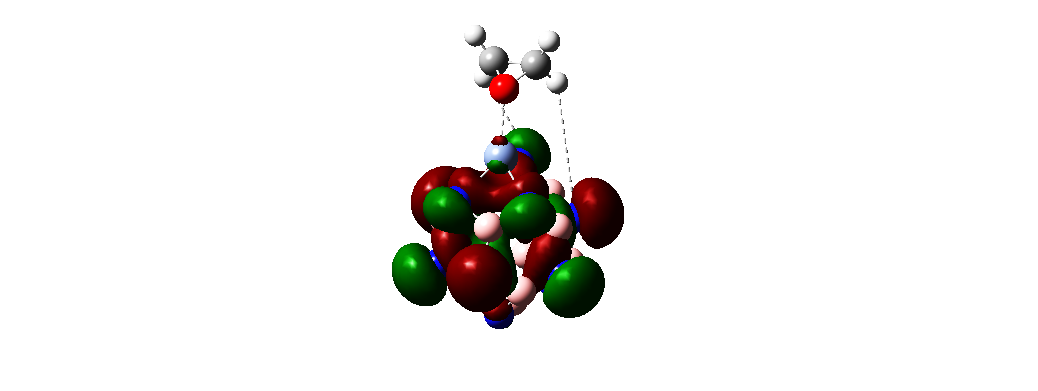** | **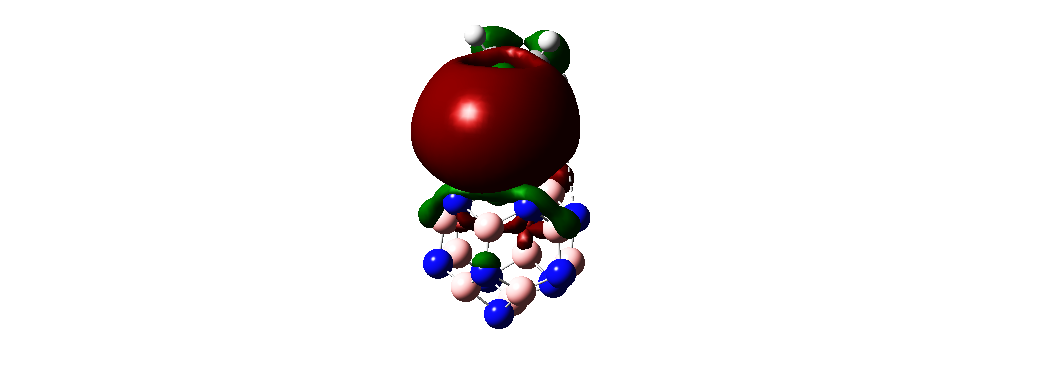** | **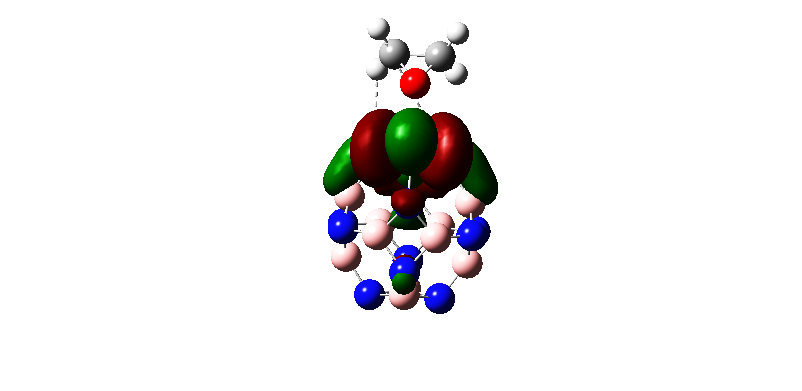** | **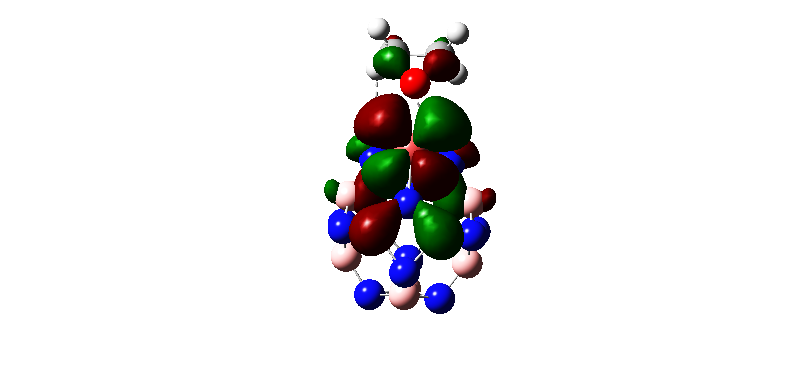** |
| 1. **Complex A** | | 1. **Complex B** | | 1. **Complex C** | |
| **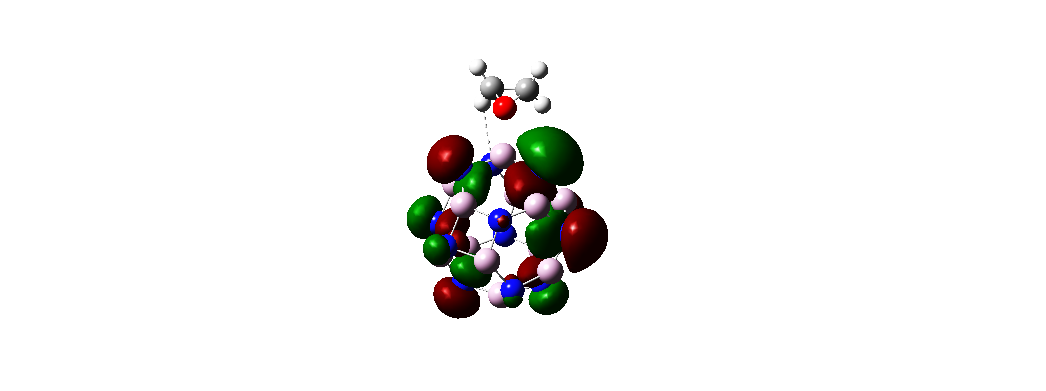** | **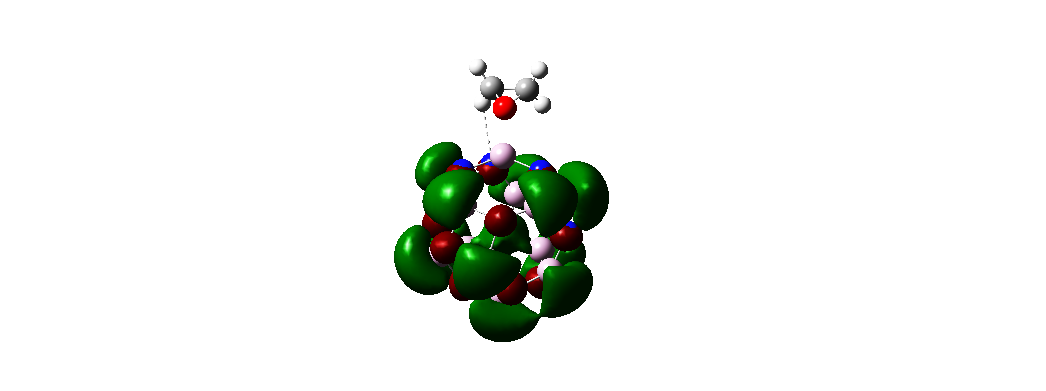** | **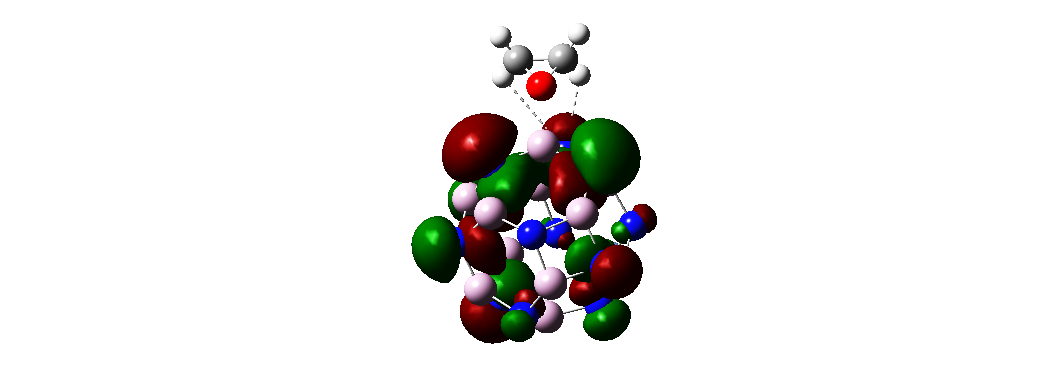** | **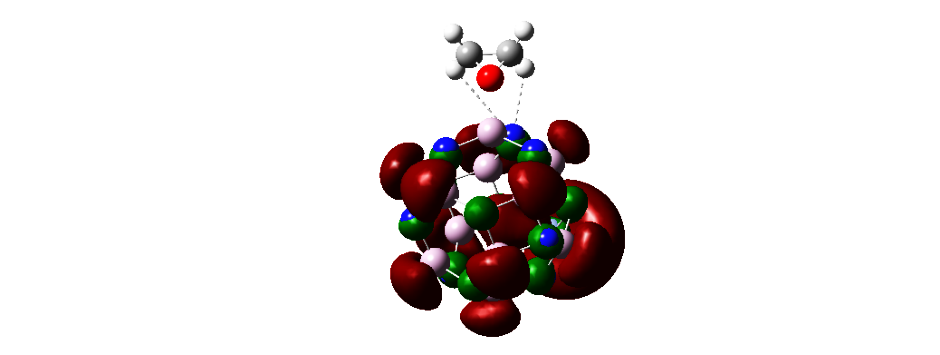** | **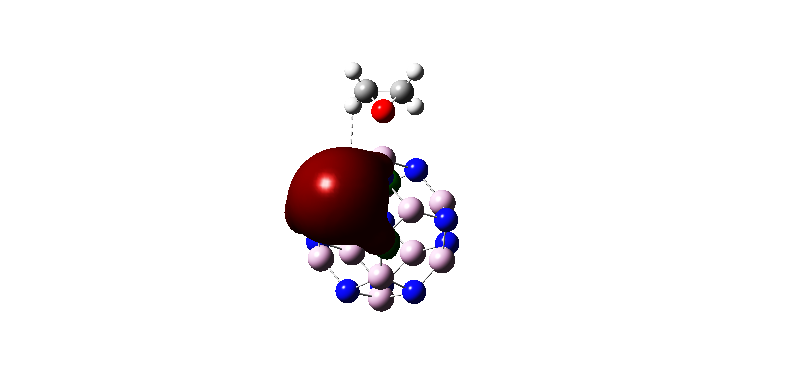** | **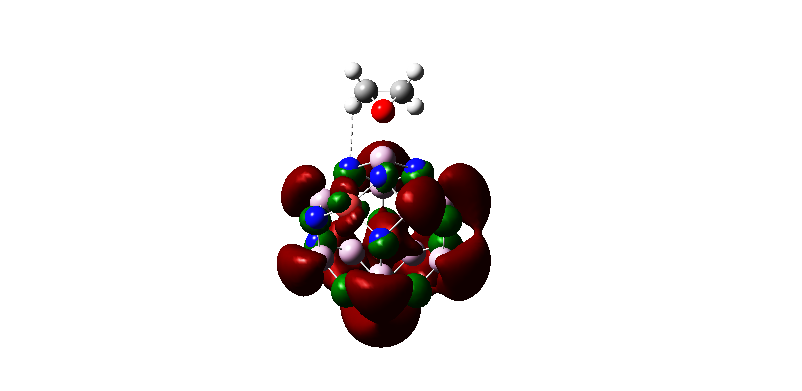** |
| 1. **Complex D** | | 1. **Complex E** | | 1. **Complex F** | |
|  |  |  |  |  |  |
| 1. **Complex G** | | 1. **Complex H** | | 1. **Complex I** | |

**Figure S6:** The HOMO and LUMO level distribution of (a) Complex A, (b) Complex B, (c) Complex C, (d) Complex D, (e) Complex E, (f) Complex F, (g) Complex G, (h) Complex H, and (i) Complex I in the B3LYP/6-31G(d,p) method. The HOMO and LUMO figures are generated with the iso-value of 0.02 electron/bohr^3^.

|  |  |  |
| --- | --- | --- |
| 1. **Complex A** | 1. **Complex B** | 1. **Complex C** |
|  |  |  |
| 1. **Complex D** | 1. **Complex E** | 1. **Complex F** |
|  |  |  |
| 1. **Complex G** | 1. **Complex H** | 1. **Complex I** |

**Figure S7:** Density of State spectrum illustration of (a) Complex A, (b) Complex B, (c) Complex C, (d) Complex D, (e) Complex E, (f) Complex F, (g) Complex G, (h) Complex H, and (i) Complex I in B3LYP/6-31G(d,p) method.

**Table S5:** Maximum adsorption wavelength ($\lambda_{\max}$) in nm, the energy associated with maximum adsorption wavelength ($E_{\lambda}$) in eV and absorbance of pristine and complex systems. This table has been taken from UV-Vis spectra in B3LYP/6-31G(d,p) method.

| System | $\boldsymbol{\lambda}_{\mathbf{max}}$ | $\mathbf{E}_{\boldsymbol{\lambda}}$ | Abs. |
| --- | --- | --- | --- |
| Pristine BN | 269.78 | 4.61 | 9958.61 |
| Complex A | 264.78 | 4.69 | 10458.83 |
| Sc-BN | 218.02 | 5.70 | 7270.76 |
| Complex B | 246.51 | 5.04 | 4260.77 |
| Ti-BN | 347.62 | 3.57 | 12419.53 |
| Complex C | 249.43 | 4.98 | 1453.84 |
| Pristine AlN | 319.78 | 3.89 | 9349.3 |
| Complex D | 306.71 | 4.05 | 5602.17 |
| Sc-AlN | 276.57 | 4.49 | 2860.34 |
| Complex E | 280.59 | 4.43 | 2680.01 |
| Ti-AlN | 348.67 | 3.56 | 12753.14 |
| Complex F | 352.31 | 3.53 | 13516.82 |
| Pristine AlP | 408.44 | 3.04 | 455.37 |
| Complex G | 370.14 | 3.36 | 497.95 |
| Sc-AlP | 369.28 | 3.36 | 2403.77 |
| Complex H | 410.57 | 3.03 | 1629.24 |
| Ti-AlP | 617.79 | 2.01 | 451.62 |
| Complex I | 494.07 | 2.51 | 564.31 |

|  |  |
| --- | --- |
| **(a)** | **(b)** |
|  |  |
| **(c)** |  |

**Figure S8:** TD-SCF DFT calculated ultraviolet-visible spectroscopy plots of (a) BN, Sc-BN, Ti-BN nanocages, and their associated complex structures altogether (b) AlN, Sc-AlN, Ti-AlN nanocages, and their associated complex structures (c) AlP, Sc-AlP, Ti-AlP nanocages, and their associated complex structures in the B3LYP/6-31G(d,p) method.

**Table S6:** QTAIM Analysis at the bond critical point (BCP), The topological variables computed include electron densities ($\rho_{b}$) and their laplacian ($\nabla^{2}\rho_{b}$), the local potential electron energy density ($V_{b}$), kinetic electron density ($G_{b}$), the negative ratio of potential and kinetic electron energy density ($-\frac{G_{b}}{V_{b}}$) and the total electron energy densities ($H_{b}$) in the atomic unit in B3LYP/6-31G(d,p) method.

| Systems | Contact Gas - Adsorbent | $\boldsymbol{\rho}_{\boldsymbol{b}}$ | $\boldsymbol{\nabla}^{\boldsymbol{2}}\boldsymbol{\rho}_{\boldsymbol{b}}$ | $\mathbf{G}_{\boldsymbol{b}}$ | $\mathbf{V}_{\boldsymbol{b}}$ | $\mathbf{H}_{\boldsymbol{b}}$ | $\mathbf{-}\frac{\mathbf{G}_{\mathbf{b}}}{\mathbf{V}_{\mathbf{b}}}$ |
| --- | --- | --- | --- | --- | --- | --- | --- |
| Complex A | B19 - O25 | 0.093224 | 0.28438 | 0.128654 | -0.186212 | -0.057558 | 0.690900694 |
| Complex B | Sc24 - O25 | 0.045156 | 0.238037 | 0.053483 | -0.047458 | 0.006025 | 1.12695436 |
| Complex C | N23 - H29 | 0.006014 | 0.020402 | 0.004112 | -0.003124 | 0.000988 | 1.316261204 |
|  | Ti24 - O25 | 0.053373 | 0.291252 | 0.065989 | -0.059165 | 0.006824 | 1.11533846 |
|  | N18 - H30 | 0.006005 | 0.020386 | 0.004108 | -0.00312 | 0.000988 | 1.316666667 |
| Complex D | Al24 - O25 | 0.048732 | 0.308898 | 0.072383 | -0.067541 | 0.004842 | 1.071689788 |
|  | N7 - H31 | 0.0115 | 0.036236 | 0.007935 | -0.006811 | 0.001124 | 1.165027162 |
| Complex E | N8 - H28 | 0.011757 | 0.036766 | 0.008086 | -0.006981 | 0.001105 | 1.158286778 |
|  | Al20 - O25 | 0.048227 | 0.304101 | 0.071308 | -0.06659 | 0.004718 | 1.070851479 |
| Complex F | N9 - H28 | 0.010304 | 0.033532 | 0.007191 | -0.005999 | 0.001192 | 1.198699783 |
|  | Al23 - O25 | 0.048709 | 0.305183 | 0.071752 | -0.067208 | 0.004544 | 1.067610999 |
|  | N12 - H31 | 0.010282 | 0.033623 | 0.0072 | -0.005995 | 0.001205 | 1.201000834 |
| Complex G | Al13 - O25 | 0.04628 | 0.271531 | 0.06513 | -0.062377 | 0.002753 | 1.044134857 |
| Complex H | Sc24 - O25 | 0.052316 | 0.281575 | 0.06358 | -0.056766 | 0.006814 | 1.120036642 |
| Complex I | Ti24 - O25 | 0.059196 | 0.354777 | 0.080586 | -0.072479 | 0.008107 | 1.111853088 |

|  |  |  |  |  |
| --- | --- | --- | --- | --- |
| 1. **Complex A** | 1. **Complex B** | 1. **Complex C** | 1. **Complex D** | 1. **Complex E** |
|  |  |  |  |  |
| 1. **Complex F** | **(g) Complex G** | **(h) Complex H** | **(i) Complex I** |  |

**Figure S9:** The molecular graphs of the (a) Complex A, (b) Complex B, (c) Complex C, (d) Complex D, (e) Complex E, (f) Complex F, (g) Complex G, (h) Complex H, and (i) Complex I at their bond critical sites in the B3LYP/6-31G(d,p) method.
